# Influence of the Fermented Feed and Vaccination and Their Interaction on Parameters of Large White/Norwegian Landrace Piglets

**DOI:** 10.3390/ani10071201

**Published:** 2020-07-15

**Authors:** Laurynas Vadopalas, Sarunas Badaras, Modestas Ruzauskas, Vita Lele, Vytaute Starkute, Paulina Zavistanaviciute, Egle Zokaityte, Vadims Bartkevics, Dovile Klupsaite, Erika Mozuriene, Agila Dauksiene, Sonata Sidlauskiene, Romas Gruzauskas, Elena Bartkiene

**Affiliations:** 1 Institute of Animal Rearing Technologies, Lithuanian University of Health Sciences, Tilžės g. 18, LT-47181 Kaunas, Lithuania; laurynas.vadopalas@lsmuni.lt (L.V.); sarunas.badaras@gmail.com (S.B.); vita.lele@lsmuni.lt (V.L.); vytaute.starkute@lsmuni.lt (V.S.); paulina.zavistanaviciute@lsmuni.lt (P.Z.); egle.zokaityte@lsmuni.lt (E.Z.); dovile.klupsaite@lsmuni.lt (D.K.); erika.mozuriene@lsmuni.lt (E.M.); agila.dauksiene@lsmuni.lt (A.D.); sonata.sidlauskiene@lsmuni.lt (S.S.); 2 Department of Physiology and Anatomy, Lithuanian University of Health Sciences, Tilžės g. 18, LT-47181 Kaunas, Lithuania; modestas.ruzauskas@lsmuni.lt; 3 Microbiology and Virology Institute, Lithuanian University of Health Sciences, Tilžės g. 18, LT-47181 Kaunas, Lithuania; 4 Department of Food Safety and Quality, Lithuanian University of Health Sciences, Tilžės g. 18, LT-47181 Kaunas, Lithuania; 5 University of Latvia Faculty of Chemistry, Jelgavas iela 1, LV-1004 Riga, Latvia; vadims.bartkevics@bior.gov.lv; 6 Institute of Food Safety, Animal Health and Environment BIOR, Lejupesiela 3, LV-1076 Riga, Latvia; 7 Department of Food Science and Technology, Kaunas University of Technology, Radvilenu str. 19, LT-50254 Kaunas, Lithuania; romas.gruzauskas@ktu.lt

**Keywords:** piglets, fermentation, antimicrobial properties, vaccination, microbiota, ammonia emission

## Abstract

**Simple Summary:**

Farm animals are constantly exposed to pathogenic microorganisms, which can lead to bacterial, as well as secondary infections caused by viruses. For this reason, vaccination is used. However, many discussions have been published about the effectiveness of this prophylactic measure. The aim of this study was to evaluate the influence of fermented feed on non-vaccinated (NV) and vaccinated with Circovac porcine circovirus type 2 vaccine piglets’ blood parameters, gut microbial composition, growth performance and ammonia emission. The 36-day experiment was conducted using 25-day-old Large White/Norwegian Landrace piglets, which were randomly divided into four groups each comprising 100 piglets (NV piglets fed with soya meal, vaccinated piglets fed with soya meal, NV piglets fed with fermented rapeseed meal and vaccinated piglets fed with fermented rapeseed meal). The results revealed that vaccination, as a separate factor, did not significantly influence piglets’ blood parameters. Finally, rapeseed meal fermented with the selected lactic acid bacteria strains can be used instead of expensive imported soya, because the fermented feed did not cause undesirable changes in piglets’ health and growth performance. Furthermore, the process is more sustainable.

**Abstract:**

The aim of this study was to evaluate the influence of fermented with a newly isolated lactic acid bacteria (LAB) strains combination (*Lactobacillus plantarum* LUHS122, *Lactobacillus casei* LUHS210, *Lactobacillus farraginis* LUHS206, *Pediococcus acidilactici* LUHS29, *Lactobacillus plantarum* LUHS135 and *Lactobacillus uvarum* LUHS245) feed on non-vaccinated (NV) and vaccinated with Circovac porcine circovirus type 2 vaccine (QI09AA07, CEVA-PHYLAXIA Co. Ltd. Szállás u. 5. 1107 Budapest, Hungary) piglets’ blood parameters, gut microbial composition, growth performance and ammonia emission. The 36-day experiment was conducted using 25-day-old Large White/Norwegian Landrace (LW/NL) piglets, which were randomly divided into four groups with 100 piglets each: S_nonV_—non-vaccinated piglets fed with control group compound feed; S_V_—vaccinated piglets fed with control group compound feed; RF_nonV_—non-vaccinated piglets fed with fermented compound feed; RF_V_—vaccinated piglets fed with fermented compound feed. Samples from 10 animals per group were collected at the beginning and end of the experiment. Metagenomic analysis showed that fermentation had a positive impact on the *Lactobacillus* prevalence during the post-weaning period of pigs, and vaccination had no negative impact on microbial communities. Although a higher amount of *Lactobacillus* was detected in vaccinated, compared with non-vaccinated groups. At the end of experiment, there was a significantly higher LAB count in the faeces of both vaccinated compared to non-vaccinated groups (26.6% for S_V_ and 17.2% for RF_V_), with the highest LAB count in the S_V_ group. At the end of experiment, the S_V_ faeces also had the highest total bacteria count (TBC). The RF_V_ group had a 13.2% increase in total enterobacteria count (TEC) at the end of experiment, and the S_V_ group showed a 31.2% higher yeast/mould (Y/M) count. There were no significant differences in the average daily gain (ADG) among the groups; however, there were significant differences in the feed conversion ratios (FCR) between several groups: S_V_ vs. S_nonV_ (11.5% lower in the S_V_ group), RF_V_ vs. RF_nonV_ (10.2% lower in the RFnonV group) and S_V_ vs. RF_V_ (21.6% lower in the S_V_ group). Furthermore, there was a significant, very strong positive correlation between FCR and TEC in piglets’ faeces (R = 0.919, *p* = 0.041). The lowest ammonia emission was in RF_V_ group section (58.2, 23.8, and 47.33% lower compared with the S_nonV_, S_V_ and RF_nonV_ groups, respectively). Notably, there was lower ammonia emission in vaccinated groups (45.2% lower in S_V_ vs. S_nonV_ and 47.33% lower in RF_V_ vs. RF_nonV_). There was also a significant, very strong positive correlation between ammonia emission and Y/M count in piglets’ faeces at the end of the experiment (R = 0.974; *p* = 0.013). Vaccination as a separate factor did not significantly influence piglets’ blood parameters. Overall, by changing from an extruded soya to cheaper rapeseed meal and applying the fermentation model with the selected LAB combination, it is possible to feed piglets without any undesirable changes in health and growth performance in a more sustainable manner. However, to evaluate the influence of vaccination and its interaction with other parameters (feed, piglets’ age, breed, etc.) on piglets’ parameters, additional studies should be performed and methods should be standardised to ensure the results may be compared.

## 1. Introduction

The challenges associated with livestock production become greater each year. This phenomenon is associated with population growth, as well as growing needs for animal-based products. Farms have become bigger, and animals are always exposed to opportunistic and pathogenic microorganisms. Most pathogen infections have a multifunctional influence on the host tissues and cells, and the response to the infection rather than the infection itself results in most of the damage and a general disturbance in physiological processes [1,2,3,4,5]. One consequence of infections on pig production is multisystemic wasting syndrome after weaning. Indeed, it is crucial to ensure piglets health at the earliest stage, because type 2 porcine circovirus (PCV2) leads to multisystemic wasting syndrome after weaning, and co-infection with other pathogens can be lethal [6,7]. Some published findings have indicated the potential participation of PPV2 in clinical diseases. PCV2 can occur as a coinfection with other pathogens in animals afflicted with respiratory disease [8], as well as in the lungs of animals with inflammatory lesions [9]. PCV2 infection leads to metabolic and neurological disorders; the latter cause multisystemic wasting syndrome [10,11,12]. To improve animal keeping conditions, understanding the interactions between desirable microorganisms and pathogens and reduce the risk of secondary infections is critical to develop preventive measures against various diseases. In this study, we hypothesised that, for vaccinated piglets, incorporating antimicrobial strains for feed fermentation can be a very attractive feeding option. Finally, incorporation and biomodification of local feedstock with antimicrobial lactic acid bacteria (LAB) strains can lead to higher value feedstock for farm animal nutrition and improve their conditions. This use can also increase the sustainability of pig farming and reduce dependence on imported soya stock. In this case, natural antimicrobial treatment, e.g., feed fermentation with antimicrobial LAB strains, is also desirable because the genetic diversity of PCV2 continuously increases, and novel subtypes are still emerging [13,14,15]. PCV2-related diseases are considered multifactorial in the swine industry [7], and they can promote co-infection with other viruses or bacteria [8,9,16]. In addition, co-infection of different viruses complicates the infection status and makes it more difficult to prevent and control the diseases [17]. Vaccines are used to prevent viral infections and antibiotics are used to treat bacterial diseases, but there is a lack of complex tools for prevention of diseases. Furthermore, viruses and bacteria can mutate very quickly, a phenomenon that remains a huge challenge for the livestock industry. Animals are always exposed to the environment, and thus changes in the microorganisms present in digestive tract should be studied to understand the mechanism of action, which can be associated with vaccination, as well as with the natural antimicrobials, as the microorganisms in the gut strongly influence the immune system and animals’ health.

The aim of this study was to evaluate the influence of fermented with a newly isolated LAB strains combination (*Lactobacillus plantarum* LUHS122, *Lactobacillus casei* LUHS210, *Lactobacillus farraginis* LUHS206, *Pediococcus acidilactici* LUHS29, *Lactobacillus plantarum* LUHS135 and *Lactobacillus uvarum* LUHS245) feed on non-vaccinated (NV) and vaccinated with Circovac porcine circovirus type 2 vaccine (QI09AA07, CEVA-PHYLAXIA Co. Ltd. Szállás u. 5. 1107 Budapest, Hungary) piglets’ blood parameters, gut microbial composition, growth performance and ammonia emission. This LAB starter combination, with antimicrobial characteristics, was used to ferment local feed stock (rapeseed meal), and the influence of changing from an extruded soya to biomodified rapeseed meal on the parameters of non-vaccinated piglets and those vaccinated with the Circovac PCV2 vaccine was evaluated.

## 2. Materials and Methods

### 2.1. LAB Strains Used for Feed Fermentation, Feed Fermentation and Fermented Feed Parameters

**Figure 1:**
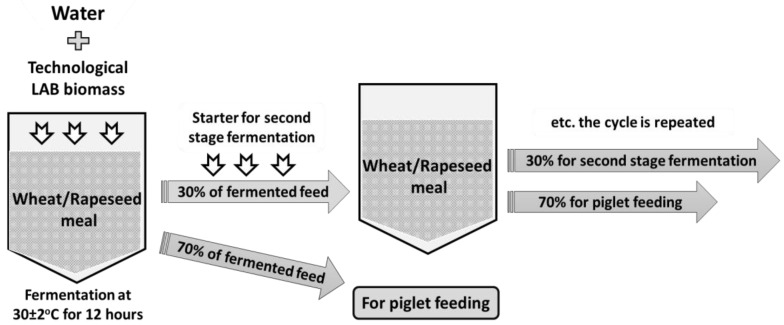
Scheme of fermented feed preparation. The technological lactic acid bacteria (LAB) biomass consisted of *Lactobacillus plantarum* LUHS122, *Lactobacillus casei* LUHS210, *Lactobacillus farraginis* LUHS206, *Pediococcus acidilactici* LUHS29, *Lactobacillus plantarum* LUHS135 and *Lactobacillus uvarum* LUHS245.

The *L. plantarum* LUHS122, *L. casei* LUHS210, *L. farraginis* LUHS206, *P. acidilactici* LUHS29, *L. plantarum* LUHS135 and *L. uvarum* LUHS245 strains were obtained from the Lithuanian University of Health Sciences collection (Kaunas, Lithuania). Our previous studies showed that the above-mentioned strains inhibit various pathogenic and opportunistic microorganisms and are suitable for fermentation of various cereal substrates [18,19,20,21,22], as well for rapeseed meal fermentation [23]. The rapeseed meal, water and LAB strains (equal parts of each strain by volume) suspension (3% from dry matter of feed mass, v/m), containing 8.9 log_10_ colony forming units CFU/mL, was fermented at 30 ± 2 °C for 12 h. The fermentation process involves wheat and rapeseed meal (1:1 ratio). The crude protein content of the rapeseed meal was 33.58%. Fermented feed was composed of 25% fermented rapeseed meal and 25% fermented wheat. This combination contained 23.24% crude protein. The compound feed for all groups contained 19% crude protein, crude fibre—3.15%, crude oil—6.51%, lysine—1.45%, methionine—0.55%, tryptophan—0.26%, threonine—0.93%, Ca—0.90%, total P—0.59% and Na—0.20%. The entire fermented wheat/rapeseed meal mass was divided in two parts: 70% (by mass) was used for piglet feeding, and 30% (by mass) was used as a starter for additional feed fermentation cycles (Figure 1). The detailed fermented feed technology and characteristics are described by Vadopalas et al. [23].

### 2.2. Animals and Housing

All animal procedures were conducted according to the European Union (EU) Directive of the European Parliament and Council, from 22 September, 2010 [24], on the protection of animals used for scientific purposes and the ‘Requirements for the Keeping, Maintenance and Use of Animals Intended for Science and Education Purposes’, approved by the order of the Lithuanian Director of the State Food and Veterinary Service [25]. The study was conducted at a pig farm in the Klaipeda district (Kontvainiai, Lithuania) and at the Institute of Animal Rearing Technologies, Lithuanian University of Health Sciences (Kaunas, Lithuania). A 36-day experiment was conducted using 25-day-old Large White/Norwegian Landrace (LW/NL) piglets with an initial body weight of 6.9–7.0 kg. Four groups were formed, each with 100 piglets: S_nonV_—non-vaccinated piglets fed with control group compound feed; S_V_—vaccinated piglets fed with control group compound feed; RF_nonV_—non-vaccinated piglets fed with fermented compound feed; RF_V_—vaccinated piglets fed with fermented compound feed. The weaner piglets were kept at the same conditions in a section with two climate zones. The first had a heated concrete floor (36 °C) and roof on it; the second had plastic piglet floors and optimum ventilated air and temperature for the active period. Drinking water and compound liquid feed were available ad libitum throughout the trial. Antibiotic treatment was not applied.

### 2.3. Experimental Design and Diets

Samples from 10 animals per group were collected at the beginning and end of experiment. Two groups (S_V_ and RF_V_) were vaccinated by intramuscular injection with Circovac PCV2 vaccine (inactivated) (QI09AA07, CEVA-PHYLAXIA Co. Ltd. Szállás u. 5. 1107 Budapest, Hungary), which is used to protect pigs against PCV2. The influence of two dietary treatments on vaccinated and non-vaccinated piglets’ parameters were compared. Fermented feed, comprising 500 g/kg of total feed, was included in the diet of treated group beginning at day 25 of life until day 61. Piglets’ growth performance was evaluated by testing all 100 piglets in each group. The basal feed was formulated according to the nutritional requirements prescribed in the Nutrient Requirements of Swine [26]. The feed composition and nutritional value are shown in Table 1. The nutritional value of compound feed was determined according to the analytic methods described by Association of Official Analytical Chemists (AOAC) [27].

### 2.4. Metagenomics and Microbial Profiling Analysis

Before the experiment, faeces from 25-day old piglets from each group (10 piglets per group) were collected. The same procedure, using 10 piglets per group, was performed at the end of the experiment (day 61 of the piglets’ life). Faecal samples from each of the group were pooled making 4 samples before and 4 samples after the experiment. Therefore, 8 samples representing 4 tested groups (before and after the experiment) were sequenced for microbial profiling. Specimens were kept in DNA/RNA Shield diluted 1:10 (R1100-250, Zymo Research, Irvine, CA USA) at −80 °C before DNA extraction. DNA was extracted with a faecal DNA MiniPrep kit (D6010, Zymo Research, Irvine, CA USA). Library preparation, metagenomic sequencing and taxonomic characterisation of reads was performed as previously described [28]. ZymoBIOMICS Microbial Community Standard (D6300, Zymo Research, Irvine, CA USA) was used as a microbiome profiling quality control. The results of taxonomic classification were visualised using the interactive online platform https://genome-explorer.com.

### 2.5. Microbiological Analysis of Faecal Samples

Piglets’ faecal samples were collected before and after the experiment, stored in vials (+4 °C) with a transport medium (Faecal Enteric Plus, Oxoid, Basingstoke, UK) and analysed on the same day. Evaluation of the microbiological parameters (LAB, total viable bacteria count [TVC], total enterobacteria count [TEC], and yeast and mould [Y/M] counts) was performed according to methods described by Zavistanaviciute et al. [29].

### 2.6. Blood Analysis

Piglets were bled from the jugular vein into vacuum blood tubes (BD Vacutainer, Wokingham, UK) before morning feeding. Tubes with clot activator were used for biochemical examination. Blood biochemical variables were evaluated before and after the experiment (on days 25 and 61 of the piglets’ life). The parameters included aspartate aminotransferase (AST), alanine aminotransferase (ALT), cholesterol (Chol), high density lipoprotein cholesterol (HDL-C), low-density lipoprotein cholesterol (LDL-C), triglycerides (TG), total protein (TP), albumin (ALB), phosphorus (IP), Mg, K, Na, triiodothyronine (T3), thyroxine (T4), immunoglobulin G (IgG), vitamin B12, albumin (ALB), Fe, glucose (GLU), Ca, creatinine analysed by the Jaffe method (CREA), alkaline phosphatase (AP), thyroid-stimulating hormone (TSH), total bilirubin and urea. Blood parameters were analysed with an automatic biochemistry analyser in the accredited laboratory ‘Anteja’ (Klaipeda, Lithuania).

### 2.7. Evaluation of Piglets’ Growth Performance and Cases of Mortality and Diarrhoea

Group body weight gain (BWG) was recorded on days 25, 32, 39, 46, 53, and 61 of age using an electronic weighing system (model type: IT1000, SysTec GmbH, Bergheim, Germany) and average daily gain (ADG) was calculated. The feed conversion ratio (FCR) was calculated from feed intake (87% of dry matter) and body weight gain (BWG). Feed consumption was measured using a WEDA (Dammann & Westerkamp GmbH, Goldenstedt, Germany) automated feeding system that has an electronic flowmeter and weighing system. Cases of mortality and diarrhoea were recorded in all tested groups throughout the experiment.

### 2.8. Analysis of Ammonia Emission

Analysis of ammonia emission was conducted according to the Environmental Protection Document LAND 88-2009 method, approved by the Nr. D1-862 order (31 December, 2009) of the Lithuanian Minister of Environment [30]. Ammonia concentration in the air was analysed by the accredited laboratory “Labtesta” (Kretinga, Lithuania). Air samples were taken on the first and the last day of experiment in four tested farm sectors: S_nonV_, S_V_, RF_nonV_, and RF_V_.

### 2.9. Statistical Analysis

Data were subjected to multivariate analysis of variance (ANOVA) using the statistical package SPSS for Windows (Ver.15.0, SPSS, Chicago, IL, USA). Baseline measurements were used as covariates to consider the experimental conditions. The mean values were compared using Duncan’s multiple range test with a significance level defined at *p* ≤ 0.05. In order to evaluate the influence of three different factors (piglets age related differences, the use of fermented feed, vaccination) and their interaction on piglets’ parameters, data were subjected to three-way ANOVA and the post hoc Tukey honest significant difference (HSD) test. In the tables, the results are presented as mean values (n = 10). Differences in bacterial genera between the groups at the end of the experiment were assessed using the Z-Test Calculator for two Population Proportions (Social Science Statistics, socscistatistics.com, 2019). The results were considered statistically significant at *p* ≤ 0.05.

## 3. Results and Discussion

### 3.1. Microbial Profiles of Pig Faeces

The number of bacterial reads in pig faeces before and after the experiment was quite similar: it varied between 35,000 and 40,000 reads among the groups. The number of species with a prevalence of at least 0.01% from the total amount of bacteria in different groups before the experiment was also similar: it varied from 400 in the RF_nonV_ group to 473 in the S_V_ group. The number of species with the same prevalence rate at the end of experiment varied from 340 to 387. Although the number of bacterial reads and bacterial composition among the groups was similar, we detected some distinct differences, particularly in the number of lactobacilli.

**Figure 2:**
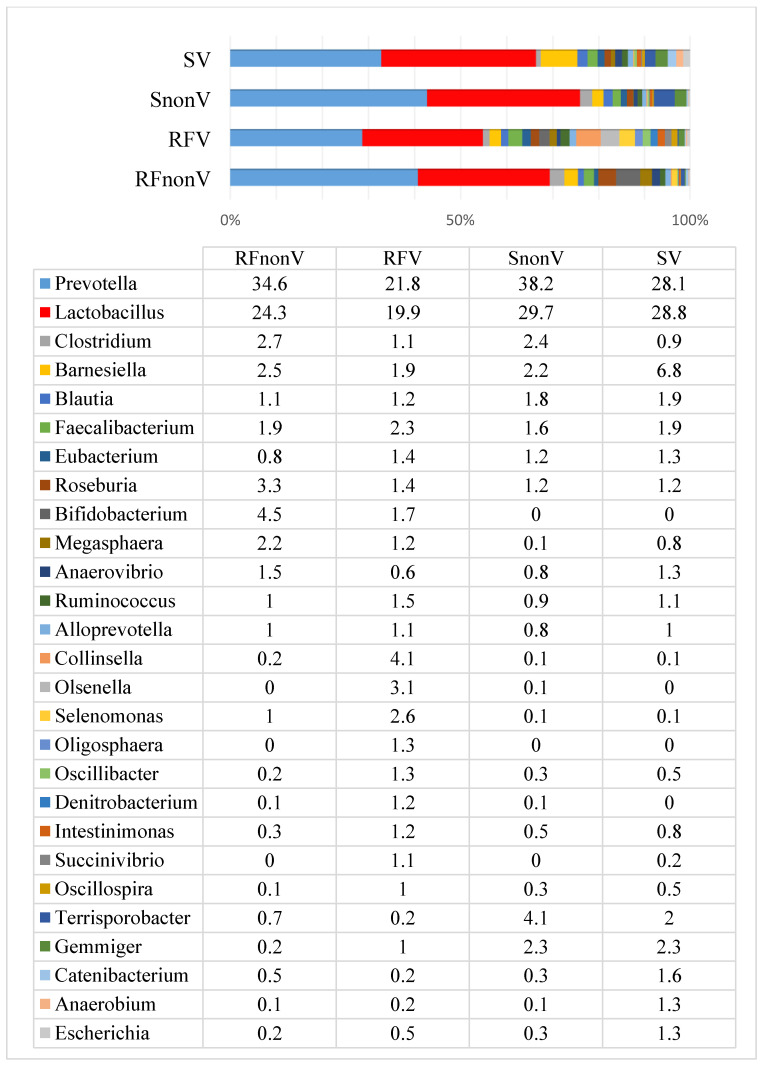
Microbial profiles (prevalence, %) at the genus level in pig faeces before the experiment.

Before experiment, two bacterial genera—*Prevotella* and *Lactobacillus*, had the highest prevalence rates in all groups of pigs; they accounted for more than 40% of the total amount of bacterial composition (Figure 2). The prevalence of *Prevotella* varied from 21.8% to 38.2%, while *Lactobacillus* prevalence ranged from 19.9% to 29.7%. The lowest *Lactobacillus* prevalence was in the RF_V_ group, while the highest was in S_nonV_ group. In all groups, the most prevalent species among *Lactobacillus* was *Lactobacillus amylovorus* (Tables S1–S4)). The other most prevalent genera included *Barnesiella*, *Clostridium*, *Blautia*, *Faecalibacterium*, *Roseburia* and *Eubacterium*, which ranged from 1.2% (*Eubacterium*) up to 3.4% (*Barnesiella*) in the faeces. Overall, the microbial profiles were similar among all the groups before the experiment (Figure 2), however, it should be mentioned that *Bifidobacterium* was detected only in two groups—RF_nonV_ and RF_V_ with the prevalence of 4.5% and 1.7% respectively.

**Figure 3:**
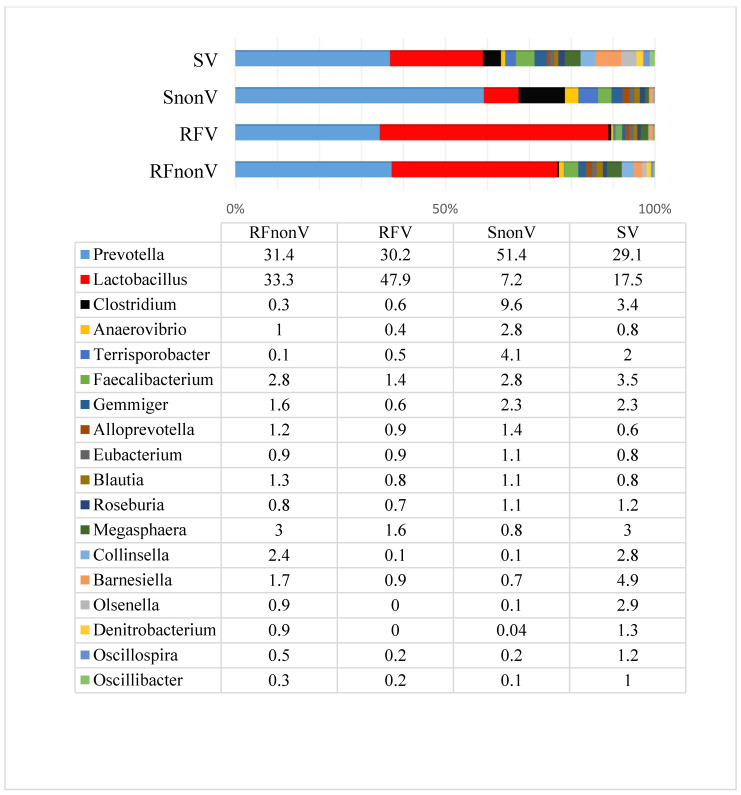
Microbial profiles (prevalence, %) at the genus level in pig faeces after experiment.

At the end of experiment, the microbial profiles had changed depending on the pig group (Figure 3). Overall, the main genera remained similar as before experiment, but there were obvious differences in the *Lactobacillus* prevalence. The lowest prevalence occurred in faeces from the S_nonV_ group, while the highest was in the RF_V_ group (*p* ≤ 0.05). In the latter group, lactobacilli were the most abundant bacteria: their prevalence reached 47.9% of the total bacteria, while in the rest of the groups the highest prevalence was *Prevotella*. When comparing groups with fermented vs. non-fermented feed, pigs that received fermented feed had a notably higher *Lactobacillus* prevalence compared to pigs fed non-fermented feed. The pigs from non-fermented feed groups had higher *Clostridium* and *Terrisporobacter* prevalence (*p* ≤ 0.05), while the prevalence of other bacterial genera was low overall in all groups.

When comparing non-vaccinated with vaccinated groups, the main differences were also associated with the *Lactobacillus* prevalence: it was higher in vaccinated compared to non-vaccinated groups (*p* ≤ 0.05). At the same time, the amount of *Prevotella* was higher in non-vaccinated groups, although the differences between RF_nonV_ and S_nonV_ groups were small (31.4% vs. 30.2%). The species prevalence and variety at the end of the experiment are presented in Tables S5–S8).

The microbial communities in the pig gut perform a variety of beneficial functions [31]. Previous studies of the gut microbial community have illustrated how populations of constituents are shaped by environmental exposure to microbes, diet, immunological pressures, host genetics and ecological forces within the ecosystem itself [32,33,34]. Understanding which microbial populations are influenced by fermented feed provides insight into how dietary changes in pigs shape the gut microbiome. Although there have been studies about microbial communities within the gut of healthy pigs, the microbial composition can differ depending on breed, age, place, feed, hygiene conditions and other factors [31,35,36]. This study demonstrated changes in microbial profiles using feed prepared by different technologies within similar groups of animals and the same farm. While the exact mechanism(s) by which vaccination can influence microbial changes within the gut of mammals remains unknown, this study suggests that vaccination has a certain influence, particularly on the number of *Lactobacillus* spp.

Before the experiment, the microbial composition in all groups was very similar, with the highest prevalence for *Prevotella* and *Lactobacillus*, both of which accounted for 56% of the bacterial count. These genera include abundant microorganisms in young weaned healthy pigs [37]. *Prevotella* spp. are usually dominant in pigs’ gut and gradually increase in number with age [38,39]. *Prevotella* spp. are key microbial members of the gastrointestinal tracts of adult animals; they are crucial for the degradation of starch and plant polysaccharides but also have a strong capacity for mucoprotein catabolism [40,41]. *Lactobacillus* spp. are common in both the proximal and distal regions of the porcine digestive tract; they colonise soon after birth [42]. This genus influences intestinal physiology, regulates the immune system and balances the intestinal ecology of the host [43]. In addition, *Lactobacillus* spp. have been known to metabolise carbohydrates, including oligosaccharides and starch, which are fermented in the large intestine to short chain fatty acids by lactobacilli for subsequent utilisation by the pigs [44]. Previous studies have indicated that stress greatly affects the gastrointestinal microbiota: it decreases total *Lactobacillus* populations and thus provides an opportunity for pathogen overgrowth [45]. Such stress usually occurs during the weaning period. According to the literature, when compared to diarrhoeic piglets, the gut microbiota of healthy piglets has a higher abundance of *Prevotellaceae*, *Lachnospiraceae*, *Ruminococcaceae* and *Lactobacillaceae* [46]. These data suggest that the gut microbial composition may be used as a biomarker to predict the health status of piglets. The present study demonstrated the positive impact of fermented feed on the porcine microbial composition compared with a conventional feeding regimen in healthy pigs. At the end of the experiment (day 61 of the piglets’ life), the *Lactobacillus* prevalence decreased on average 2.4 fold in the groups fed non-fermented feed, whereas the prevalence increased on average 1.8 fold in the groups fed fermented feed. Hence, providing fermented feed prevents the decrease in *Lactobacillus* prevalence after weaning. This effect may help prevent digestive disorders associated with microbial changes because this period is one of the most critical regarding different infections [46]. It is known that during this age, *Lactobacillus* spp. in pigs normally decrease in number [37]. The *Lactobacillus* prevalence was also higher in vaccinated compared with non-vaccinated groups irrespective of the feed type. However, the data do not indicate whether vaccination against PCV2 leads to an increase in *Lactobacillus* prevalence; more experiments are required in this area. Moreover, different types of vaccines and larger experiments should be performed to better understand the possible influence of vaccination on the microbial communities. According to this study, it may be assumed that at the very least vaccination against PCV2 does not negatively affect the microbial composition within the gastrointestinal tract of pigs.

### 3.2. LAB, TVC, TEC and M/Y Counts in Piglets’ Faeces

Microbiological parameters of the piglet faeces (from 25- and 61-day-old piglets) are shown in Table 2 (Table S9—Differences between microbiological parameters of the piglets’ faeces between all the tested groups). In most groups (except the S_V_ group), the LAB count was significantly lower at the end compared with the beginning of the experiment (20.1% lower in the S_nonV_ group; 37.9% lower in the RF_nonV_ group; and 25.3% lower in the RF_V_). The LAB count at the end of experiment was significantly higher in both vaccinated compared with non-vaccinated groups (26.6% for S_V_ and 17.2% for RF_V_). The LAB count was 26.3% higher in S_V_ compared to the RF_V_ group at the end of the experiment. In a previous study, the faeces of pigs receiving a diet with fermented feed contained significantly fewer total bacteria and fungi, as well as coliform bacteria (including Escherichia coli) and anaerobic *Clostridium perfringens* counts. This phenomenon is due in part to the reduction in pH, an increase in the amount of lactic acid and other volatile fatty acids in the intestinal contents and a reduction in the number of *Enterobacteriaceae* [47,48]. From birth until weaning and then during the post-weaning period, the gut microbiota is dynamic and undergoes major compositional changes that are driven by age, exposure to microbes, environmental conditions and diet [49]. Many authors have described the great impact of early-life events in mammals, and particularly in pigs, on their future health. These experiences shape immune system development through changes in the pattern of microbial intestinal colonisation [50,51]. Colonisation is initiated at birth and is shaped by consumption of the sow’s milk, which provides nutritional advantages to the LAB population, building a milk-oriented microbiome that includes *Bacteroidaceae* and *Lactobacillaceae*. This composition rapidly changes after weaning when a (largely) plant-based diet is introduced [35]. The rapidly varying microbiome of young piglets seems to increase in microbial diversity and richness in the suckling phase and gradually stabilises post-weaning [35,52,53]. Our results are in agreement with Bian et al. [54], namely that members of the *Lactobacillaceae* became predominant on days 7, 14 and 28, but had a lower relative abundance again on day 49.

When comparing the TVC in piglets’ faeces, in all but the S_V_ group it was significantly lower at the end of experiment compared with the beginning (9.5% reduction in the S_nonV_ group; 23.6% reduction in the RF_nonV_ group; 3.4% reduction in the RF_V_ group). TVC was significantly higher in the vaccinated piglets compared to the non-vaccinated piglet faeces at the end of the experiment (22.2% for S_V_ and 13.8% for RF_V_). Between the vaccinated groups, TVC was 10.0% higher in the S_V_ compared to the RF_V_ group. During weaning, there is gut microbiota dysbiosis, including a loss of microbial diversity. Studies have noted significant reductions in total bacterial number, including coliform bacteria and anaerobic *C. perfringens*, as well as an increase in *Acetivibrio*, *Dialister*, *Oribacterium*, *Prevotella* and *Proteobacteriaceae*, including *E. coli* [48,49,55,56]. A decrease in *Lactobacillus* spp. can lead to decrease in TVC. Zimmerman et al. [57] demonstrated that *Lactobacillus*, *Bifidobacterium* and *Lactobacillus* spp. have a positive immunomodulatory effect on vaccines in animals.

In all the groups, the TEC and Y/M counts were lower in piglets’ faeces at the end compared with the beginning of the experiment (11.9 and 5.5%, respectively, for the S_nonV_ group; 26.1 and 44.1%, respectively, for the S_V_ group; 7.2 and 7.9%, respectively, for the RF_nonV_ group; 6.1 and 5.6%, respectively, for the RF_V_ group). Both TEC and Y/M were significantly lower in the S_V_ compared to S_nonV_ group faeces at the end of experiment, but the opposite was true for the RF_V_ compared to the RF_nonV_ group. At the end of the experiment, the TEC and Y/M counts were higher in the RF_V_ compared to the S_V_ group (13.2 and 31.2%, respectively). Our results are in agreement with Dowarah et al. [58], who showed that fermented feed improved the number of beneficial microbes (LAB and bifidobacteria) and reduced *E. coli* and clostridia count, the pathogens responsible for diarrhoea, in faces. Arfken et al. [59] investigated the mutualistic relationship between yeast in the piglet gastrointestinal (GI) tract and *Lactobacillus* count. There was a significant reduction in the total number of fungi compared with the control group [48].

The ANOVA results indicated that there were significant treatment duration, fermented feed and vaccination main effects, as well as significant treatment duration × fermented feed, treatment duration × vaccination, fermented feed × vaccination and treatment duration × fermented feed × vaccination interactions on microbiological parameters of the pig faeces. Indeed, only the fermented feed × vaccination interaction was not significant for TVC in pig faeces.

Finally, microbiological parameters of the piglets’ faeces were varied and, in most of the cases, influenced by the analysed factors and their interaction. Considering that genera and species as well as the interaction between strains can lead changes of piglets’ growth performance, correlation between the above-mentioned parameters were calculated.

### 3.3. Piglet Blood Parameters

The piglets’ blood parameters are shown in Table 3 (Table S10—Differences between blood parameters of the piglets’ between all the tested groups). At the end of experiment, there were significantly lower ALT, T3, T4, IP, Mg, K, Ca and vitamin B12 concentrations in the S_V_ compared with the S_nonV_ group. However, in the S_V_ group, Chol, HDL-C, TP and GLU concentrations were significantly higher compared with the SnonV group. At the end of experiment, there were significantly lower ALT, HDL-C, LDL-C, TP, ALB and Fe blood concentrations in the RF_V_ compared to RF_nonV_ group. However, in the RF_V_ group blood, TG and total bilirubin concentrations were significantly higher compared with the RF_nonV_ group.

When comparing blood parameters between the vaccinated groups (S_V_ and RF_V_) at the end of experiment, ALT, TG, IgG, Mg, K, Fe and total bilirubin were significantly higher in the RF_V_ group. By contrast, HDL-C, LDL-C, TP, ALB, T3, T4 and vitamin B12 were significantly lower in the RF_V_ compared with the S_V_ group.

In general, blood biochemical indices reflect comprehensive functions of piglet’s nutritional metabolism, health and welfare [60]. The IgG level reflects immune status, plays important roles in the humoral immune response and controls bacterial infections in the body; it can also function to control diarrhoeal infections by binding multiple pathogenic antigens [46]. Lu et al. [59] reported that IgG and immunoglobulin M (IgM) concentrations were significantly greater and serum ALT and AST concentrations were decreased in pigs that received fermented feed (*p* ≤ 0.05). The activities of the hepatic enzymes ALT and AST are key indices that reflect the acute injury of liver. The ALT blood concentration increases when the liver cell membrane is damaged. The AST content significantly increases when the liver mitochondrial membrane is impaired [60]. The blood TP concentration reflects the relationship between protein absorption in vivo and humoral immunity [60]. The hydrolysis products of glucosinolates are known to depress iodine metabolism in the thyroid gland and inhibit the synthesis of thyroid hormones T3 and T4. When these compounds, especially thiocyanates, interfere with iodine uptake, hypothyroidism and thyroid gland enlargement may ensue [46]. However, in this study the utilised rapeseed meal contained very low concentrations of erucic acid and glucosinolates (7.5–11.2 μmol/g). Hence, the influence of the above-mentioned compounds on T3 and T4 should be minimal. The fermentation process can reduce dietary anti-nutrients (phytates, glucosinolates and trypsin inhibitors) and improve the absorption and use of nutrients, including amino acids and minerals (e.g., P, Ca, Zn, Cu and Fe) [48,61]. These changes can lead to haematological, biochemical and mineral profile variations in the blood [61,62]. However, it was published that the TP concentration was increased in the blood of piglets fed fermented feed [63]. In opposition to above-mentioned findings, Min [64] reported that the TP blood concentration was not affected by the fermented feed diets. In serum samples from piglets fed with fermented feed, there were higher ALP, TP, ALB and GLU concentrations [62,65,66]. Liu et al. [15] published that piglets fed fermented feed had lower levels of serum IgG, but there was no difference in the immunoglobulin IgA and IgM serum levels. Furthermore, the serum glucose level was decreased in piglets fed fermented feed; however, TP, Chol, TG, LDL-C, ALT, AST, Ca, P and Mg were not different among the treatments [67]. Satessa et al. [68] found that fermented rapeseed meal reduced the glucose, HDL-C and LDL-C concentrations, but TP, P, AST, ALT concentrations were increased, and significant changes in blood IgG were not established. According to Czech et al. [61], the blood concentrations of Chol and TG AST in piglets fed with fermented rapeseed meal was reduced, and there was better availability of minerals.

The influence of the analysed factors and their interaction on piglets’ blood parameters is shown in Table 4. Vaccination as a separate factor did not significantly influence piglets’ blood parameters. However, the treatment duration × fermented feed interaction was significant for T3 (*p* = 0.038), T4 (*p* = 0.041) and Fe (*p* = 0.008) concentrations in piglets’ blood. There was a significant treatment duration × vaccination interaction on T4 (*p* = 0.008) and K (*p* = 0.007) concentrations in piglets’ blood. Finally, there was a significant fermented feed × vaccination interaction for T4 (*p* = 0.033), vitamin B12 (*p* = 0.003) and urea (*p* = 0.014) concentrations in piglets’ blood. All three factors were significant with regard to the TP content in piglets’ blood (*p* = 0.041).

### 3.4. Piglets’ Growth Performance

The piglets’ average FCR, ADG and the influence of analysed factors and their interaction on piglets’ growth performance parameters from day 25 to 61 are shown in Table 5. For ADG, there were no significant differences among the groups. In addition, the analysed factors as well as their interactions did not exert significant effects on piglets’ ADG. However, there were significant differences in FCR between S_V_ and S_nonV_ (11.5% lower in the SV group), between RF_V_ and RF_nonV_ (10.2% lower in the RF_nonV_ group) and between S_V_ and RF_V_ (21.6% lower in the S_V_ group). There was a significant, very strong positive correlation between FCR and TEC in piglets’ faeces (R = 0.919, *p* = 0.041). There were no significant correlations between the other analysed faecal microbiological parameters (LAB, TBC and Y/M count) and FCR or ADG.

Mortality and diarrhoea cases were similar in all groups throughout the experiment. The mortality of piglets in the non-vaccinated groups (S_nonV_ and RF_nonV_) was 2%, while mortality in the vaccinated groups (S_V_ and RF_V_) was 2% and 3%, respectively. There was more intense diarrhoea in the S_nonV_ group at day 31 and 36. There were several diarrhoea cases in the S_V_, RF_nonV_ and RF_V_ groups from day 28 to 46.

The available published information about the influence of vaccination on piglets’ growth performance is varied. According to Oliver-Ferrando et al. [69], the optimal time for PCV2 vaccination is at either 3 or 6 weeks of age. In our study, piglets were vaccinated on day 25 of life. Duivon et al. [70] published that the added PCV2 valence in the vaccination protocol helps counter the negative impact of subclinical PCV2 infection on growth. Jeong et al. [71] indicated that ADG showed improvement in vaccinated compared with non-vaccinated animals. Da Silva et al. [72] compared the ADG between vaccinated and non-vaccinated animals from weaning to finishing for four commercial vaccine products. All products significantly increased ADG values. However, according to Woźniak et al. [73] data from four farms where vaccination was used, the results were similar to those from non-vaccinated farms. The authors suggested revising the vaccination protocol. The above-mentioned findings can be explained by a major PCV2 genotype shift from the predominant PCV2 genotype 2b towards 2d [74]. While the commercial vaccines that were first introduced to the US in 2006 have been highly effective in reducing clinical signs and improving production, recent studies have indicated a declining level of PCV2 prevalence and viraemia in the field. Hence, the efficiency of current vaccines against new and emerging strains, as well as new vaccine development, is crucial [75]. Vaccination against PCV2 is an important co-working agent that confers unintended benefits in the protection against the other agents [76]. Notably, the breed information is frequently omitted from experimental publications of vaccine studies, as well as environmental conditions (temperature, stocking density, etc.). These factors are very important for the further standardisation across studies before logical comparisons can be made among studies [77].

While we found differences in the ADG between different groups, the influence of vaccination on different feed groups was the opposite. Specifically, the FCR was lower in the S_V_ compared to the S_nonV_ group. However, the effect was the opposite in the rapeseed meal groups: FCR was lower in the R_nonV_ compared to the RF_V_ group. The lowest FCR (1.38) occurred in the S_V_ group. Notably, soya-based feed is much more expensive compared with the feed composed of local rapeseed meal. Overall, the data support the use of fermented local feedstock for piglet feeding because it provides similar growth parameters as imported soya.

### 3.5. Influence of Analysed Factors on Ammonia Emission

Table 6 presents the influence of the analysed factors on ammonia emission. At the end of experiment, the RF_V_ group had the lowest ammonia emission: 58.2, 23.8 and 47.33% lower than the S_nonV_, S_V_, and RF_nonV_ groups, respectively. There was lower ammonia emission in vaccinated groups at the end of the experiment: 45.2% lower in the S_V_ compared to S_nonV_ group and 47.33% lower in the RF_V_ compared to the RF_nonV_ group. ANOVA revealed a significant effect for all the analysed factors and their interaction on ammonia emission. Furthermore, there was a significant, very strong positive correlation between ammonia emission and Y/M count in piglets’ faeces at the end of experiment (R = 0.974; *p* = 0.013).

There has been no information published about the influence of vaccination on ammonia emission; most of the published studies have focussed on dietary aspects. Yi et al. [37] and Lee et al. [78] reported that protected organic acids used for weanling pigs reduce faecal ammonia. Nguyen et al. [79] concluded that diets with probiotics mixture decreased pigs’ ammonia emission. According to Wang et al. [51], diets with fermented feed ingredients tended to decrease ammonia emissions, but this effect was not statistically significant. According to Bindas et al. [80], ammonia in the faeces of experimental animals fed with fermented feed was significantly lower compared with the control animals. Reducing ammonia emissions by changing the dietary composition is considered economical and feasible because it can improve nitrogen utilisation and, consequently, reduce ammonia emissions [53]. Lactobacilli-based feed fermentation decreases the emissions of total organic carbon and ammonium [67]. Finally, many factors can contribute to ammonia emission on pig farms. Our data indicate that vaccination as a factor should be considered, as well as microbiological parameters of the piglets’ faeces. Indeed, we found a very strong correlation between ammonia emission and Y/M count in piglets’ faeces.

## 4. Conclusions

Fermented feed had a positive impact on the amount of *Lactobacillus* during the post-weaning period of pigs. In addition, vaccination had no negative impact on microbial communities. There was a higher *Lactobacillus* prevalence in vaccinated compared with non-vaccinated groups, as well as a higher viable LAB count in faeces from both vaccinated groups (S_V_ and RF_V_) at the end of experiment. There were no significant differences in ADG between piglets’ groups. There were differences in FCR: 11.5% lower in the S_V_ compared with the S_nonV_ group, 10.2% lower in the RF_nonV_ compared with the RF_V_ group, and 21.6% lower in the S_V_ compared with the RF_V_ group. There were a significant, very strong positive correlations between FCR and TEC in piglets’ faeces (R = 0.919, *p* = 0.041) and between ammonia emission and Y/M count in piglets’ faeces at the end of experiment (R = 0.974, *p* = 0.013). The lowest ammonia emission was in the RF_V_ group section. Vaccination as a separate factor did not significantly influence piglets’ blood parameters. Overall, by changing from an extruded soya to cheaper rapeseed meal and applying the fermentation model with the selected LAB combination, it is possible to feed piglets without any undesirable changes in health and growth performance, as well as in a more sustainable manner. However, to evaluate the influence of vaccination and its interaction with other parameters (feed, piglet age, breed, etc.) on piglets’ parameters, additional studies should be performed and methods should be standardised so that the results may be compared.

## Figures and Tables

**Table 1:** Diet composition (dry matter 87 %).

Ingredients (%)	Control Group	Treated Group
Barley	38.45	33.25
Rapeseed meal	-	25.00
Wheat	32.12	25.00
Soya beans (extruded)	9.30	-
Potato protein	5.00	2.00
Soybean protein concentrate	2.00	-
Whey powder	5.80	5.80
Sunflower oil	2.72	4.51
Limestone	1.48	1.1
NaCl	0.38	0.35
Monocalcium phosphate	0.33	0.41
L-Lysine sulphate	0.87	1.1
DL-Methionine	0.25	0.16
Acidal NC (formic and acetic acids)	0.30	0.30
^1^ Vitamins and trace elements (premix)	1.00	1.00
Nutritional value		
ME swine (MJ/kg)	13.86	13.95
Crude protein (%)	19.00	19.00
Crude fat (%)	6.51	6.51
Crude fibre (%)	3.15	5.14
Lysine (%)	1.45	1.45
Methionine (%)	0.55	0.55
Threonine (%)	0.93	0.94
Tryptophan (%)	0.26	0.25
Methionine + Cystine (%)	0.87	0.88
Ca (%)	0.90	0.90
Total P (%)	0.59	0.62
Available P (%)	0.37	0.38
Na (%)	0.20	0.21

ME—metabolisable energy. ^1^ Composition of premix per 1 kg of feed: Vitamin A—18,180 IU; vitamin D3—2040 IU; vitamin E—161 mg kg^−1^; vitamin K3—5.03 mg; thiamine—3.64 mg; riboflavin—9.16 mg; choline chloride—404 mg; pyridoxine—4.60 mg; vitamin B12—0.05 mg; niacin—41 mg; pantothenic acid—22.85 mg; folic acid—1.85 mg; biotin—0.21 mg; Fe—152 mg; Cu—100 mg; Zn—91 mg; Mn—80 mg; I—0.81 mg; Co—0.53 mg; Se—0.30 mg.

**Table 2:** Microbiological parameters in faeces from 25- and 61-day-old piglets.

Microbiological Parameters (log_10_ CFU/g)	Day		Treatments				
			S_nonV_	S_V_		RF_nonV_	RF_V_
LAB	Baseline		7.78 ± 0.03 ^A;a^	8.22 ± 0.04 ^A;b^		8.33 ± 0.05 ^A;a^	8.35 ± 0.06 ^A;a^
	61		6.22 ± 0.04^B;a^	8.47 ± 0.02 ^B;b^		5.17 ± 0.06 ^B;a^	*6.24 ± 0.07 ^B;b^*
TVC	Baseline		7.08 ± 0.02 ^A;a^	8.25 ± 0.05 ^A;b^		8.38 ± 0.07 ^A;b^	7.68 ± 0.07 ^A;a^
	61		6.41 ± 0.04. ^B;a^	8.24 ± 0.03. ^A;b^		6.40 ± 0.03. ^B;a^	7.42 ± 0.08 ^B;b^
TEC	Baseline		7.22 ± 0.06 ^A;a^	7.79 ± 0.06 ^A;b^		7.40 ± 0.08 ^A;a^	7.58 ± 0.04 ^A;b^
	61		6.36 ± 0.02 ^B;b^	6.18 ± 0.05 ^B;a^		6.87 ± 0.07 ^B;a^	7.12 ± 0.03 ^B;b^
Y/M	Baseline		6.73 ± 0.04 ^A;a^	7.71 ± 0.08 ^A;b^		6.21 ± 0.04 ^A;a^	6.63 ± 0.04 ^A;b^
	61		6.36 ± 0.08 ^B;b^	4.31 ± 0.06 ^B;a^		5.72±0.05 ^B;a^	6.26±0.04 ^B;b^
Tests of between-subject effects: influence of the analysed factors and their interactions on microbiological parameters in piglets’ faeces							
**Dependent Variable**	**Treatment Duration**	**Fermented Feed**	**Vaccination**	**Treatment Duration** **× Fermented Feed**	**Treatment Duration** **× Vaccination**	**Fermented Feed × Vaccination**	**Treatment Duration** **× Fermented Feed × Vaccination**
Significance of the differences between groups (*p*)							
LAB	0.0001	0.0001	0.0001	0.0001	0.0001	0.0001	0.0001
TBC	0.0001	0.0001	0.0001	0.0001	0.0001	0.246	0.0001
TVC	0.0001	0.0001	0.001	0.0001	0.021	0.0001	0.0001
Y/M	0.0001	0.0001	0.0001	0.0001	0.0001	0.0001	0.0001

S_nonV_—non-vaccinated piglets fed with control group compound feed; S_V_—vaccinated piglets fed with control group compound feed; RF_nonV_—non-vaccinated piglets fed with fermented compound feed; RF_V_—vaccinated piglets fed with fermented compound feed. CFU—colony forming units; LAB—lactic acid bacteria; TVC—total viable bacteria count; TEC—total enterobacteria count; Y/M—yeast/mould count. Measurements were made at baseline—day 25, before the start of the feeding experiment—and on day 61, the end of the experiment. ^A,B^ Different uppercase letters indicate significant treatment duration-related differences (*p* ≤ 0.05). ^a,b^ Different lowercase letters indicate differences among treatments, comparing S_nonV_ and S_V_; RF_nonV_ and RF_V_ (*p* ≤ 0.05). Data are presented as mean values ± standard error (n = 10 per group).

**Table 3:** Piglets’ blood parameters.

Blood Parameters	Day	Treatments			
		S_nonV_	S_V_	RF_nonV_	RF_V_
Aspartate aminotransferase (AST), U/L	Baseline	29.4 ^A;a^	42.67 ^A;a^	51.4 ^A;a^	61.8 ^A;a^
	61	34.0 ^B;a^	41.0. ^A;a^	44.0. ^A;a^	42.4. ^A;a^
Alanine aminotransferase (ALT), U/L	Baseline	48.4 ^A;a^	43.67 ^A;a^	53.2 ^A;b^	60.8 ^A;a^
	61	76.2. ^B;a^	69.6 ^B;b^	87.0 ^B;a^	80.8 ^A;b^
Cholesterol (Chol), mmol/L	Baseline	1.64 ^A;a^	1.64 ^A;a^	1.88 ^A;a^	2.04 ^A;a^
	61	2.06. ^B;a^	2.26. ^B;b^	2.34. ^A;a^	2.25. ^B;a^
High-density lipoprotein cholesterol (HDL-C), mmol/L	Baseline	0.714 ^A;a^	0.744 ^A;b^	0.898 ^A;a^	0.846 ^A;a^
	61	0.840 ^B;a^	0.944 ^B;b^	1.028 ^B;a^	0.872 ^A;b^
Low-density lipoprotein cholesterol (LDL-C), mmol/L	Baseline	0.758 ^A;a^	0.726 ^A;a^	0.814 ^A;a^	0.976 ^A; a^
	61	0.980 ^B;a^	1.102 ^B;a^	1.032 ^A;a^	0.860 ^A; b^
Triglycerides (TG), mmol/L	Baseline	0.360 ^A;a^	0.372 ^A;a^	0.366 ^A;a^	0.478 ^A;a^
	61	0.466 ^B;a^	0.466 ^A;a^	0.620 ^B;a^	0.650 ^B;b^
Total protein (TP), g/L	Baseline	46.2 ^A;a^	44.9 ^A;a^	44.2 ^A;a^	45.6 ^A;a^
	61	51.8 ^B;a^	56.3 ^B;b^	52.8 ^B;a^	49.5 ^B;b^
Albumin (ALB), g/L	Baseline	30.0 ^A;a^	29.0 ^A;a^	32.6 ^A;a^	32.8 ^A;a^
	61	35.8 ^A;a^	37.4 ^B;a^	36.2 ^A;a^	33.4 ^B;b^
Immunoglobulin IgG, g/L	Baseline	2.65 ^A;a^	2.85 ^A;a^	2.35 ^A;a^	2.96 ^A;a^
	61	3.74 ^B;a^	3.44 ^A;a^	3.05 ^B;a^	5.45 ^A;b^
Triiodothyronine (T3), nmol/L	Baseline	1.21 ^A;a^	1.14 ^A;b^	1.29 ^A;a^	1.22 ^A;a^
	61	2.14^B;a^	2.02 ^B;b^	1.59 ^A;a^	1.49 ^A;a^
Thyroxine (T4), µ d/L	Baseline	4.50 ^A;a^	4.82 ^A;a^	3.50 ^A;a^	5.22 ^A;a^
	61	4.84 ^A;a^	3.84 ^A;b^	2.92 ^B;a^	2.90 ^A;a^
Glucose (GLU), nmol/L	Baseline	5.84 ^A;a^	6.10 ^A;a^	6.12 ^A;a^	5.66 ^A;a^
	61	5.74 ^A;a^	6.22 ^A;b^	6.08 ^A;a^	6.18 ^B;a^
Phosphorus (IP), mmol/L	Baseline	2.94 ^A;a^	2.87 ^A;a^	2.61 ^A;a^	2.76 ^A;a^
	61	3.50 ^B;a^	3.39 ^A;b^	3.28 ^B;a^	3.39 ^B;a^
Magnesium (Mg), mmol/L	Baseline	1.02 ^A;a^	0.928 ^A;b^	0.996 ^A;a^	0.932 ^A;b^
	61	1.07 ^A;a^	0.936 ^A;b^	0.960 ^A;a^	0.966 ^A;b^
Potassium (K)	Baseline	4.96 ^A;a^	5.66 ^A;b^	4.65 ^A;a^	5.69 ^A;b^
	61	5.81 ^B;a^	5.34 ^B;b^	4.96 ^A;a^	4.74 ^B;a^
Sodium (Na)	Baseline	143.4 ^A;a^	141.0 ^A;a^	144.0 ^A;a^	142.0 ^A;b^
	61	147.2 ^A;a^	147.2 ^B; a^	146.6 ^B;a^	144.4 ^A;a^
Iron (Fe), µmol/L	Baseline	23.6 ^A;a^	23.8 ^A;a^	31.5 ^A;a^	23.5 ^A;b^
	61	28.1 ^A;a^	26.9 ^B;a^	47.1 ^A;a^	51.4 ^B;a^
Calcium (Ca), nmol/L	Baseline	2.60 ^A;a^	2.57 ^A;a^	2.71 ^A;a^	2.55 ^A;a^
	61	2.87 ^A;a^	2.78 ^B;b^	2.79 ^A;a^	2.78 ^B;a^
Vitamin B12, pmol/L	Baseline	142.2 ^A;a^	93.8 ^A;b^	78.2 ^A;a^	131.0 ^A;b^
	61	214.6 ^A;a^	122.2 ^B;b^	94.2 ^A; a^	98.2 ^B;a^
Creatinine (CREA), µmol/L	Baseline	64.2 ^A;a^	57.4 ^A;a^	78.8 ^A;a^	63.4 ^A;a^
	61	57.4 ^A;a^	54.8 ^A;a^	48.2 ^B;a^	49.4 ^A;a^
Alkaline phosphatase (AP), U/L	Baseline	336.2 ^A;a^	285.0 ^A;a^	408.6 ^A;a^	318.0 ^A;a^
	61	263.6 ^A;a^	220.4 ^A;a^	242.6 ^B;a^	245.4 ^A;a^
Urea, mmol/L	Baseline	2.36 ^A;a^	3.26 ^A;a^	2.64 ^A;a^	2.58 ^A;a^
	61	2.02 ^A;a^	2.38 ^A;a^	3.19 ^B;a^	2.63 ^B;a^
Thyroid-stimulating hormone (TSH)	Baseline	0.0200 ^A;a^	0.0190 ^A;a^	0.0208 ^A;a^	0.0236 ^A;a^
	61	0.0208 ^A;a^	0.0228 ^A;a^	0.0230 ^A;a^	0.0260 ^B;a^
Total bilirubin (pmol/L)	Baseline	1.88 ^A;a^	1.99 ^A;a^	1.99 ^A;a^	1.99 ^A;a^
	61	1.98 ^A;a^	1.99 ^A;a^	1.99 ^A;a^	2.65 ^A;b^

S_nonV_—non-vaccinated piglets fed with control group compound feed; S_V_—vaccinated piglets fed with control group compound feed; RF_nonV_—non-vaccinated piglets fed with fermented compound feed; RF_V_—vaccinated piglets fed with fermented compound feed. Measurements were made at baseline—day 25, before the start of the feeding experiment—and on day 61, the end of the experiment. ^A,B^ Different uppercase letters indicate significant treatment duration-related differences (*p* ≤ 0.05). ^a,b^ Different lowercase letters indicate differences among treatments: S_nonV_ and S_V_; RF_nonV_ and RF_V_ (*p* ≤ 0.05). Data are presented as mean values (n = 10 per group).

**Table 4:** Tests of between-subject effects: influence of the analysed factors and their interaction on piglets’ blood parameters.

Dependent Variable	TD	FF	V	TD × FF	TD × V	FF × V	TD × FF × V
Significance (*p*)							
AST, U/L	0.279	**0.025**	0.191	0.182	0.404	0.598	0.791
ALT, U/L	**0.0001**	**0.017**	0.557	0.997	0.358	0.453	0.481
Chol, mmol/L	**0.004**	0.088	0.608	0.467	0.907	0.809	0.396
HDL-C, mmol/L	**0.069**	0.123	0.768	0.501	0.905	0.185	0.482
LDL-C, mmol/L	**0.045**	0.723	0.807	0.143	0.584	0.760	0.149
TG, mmol/L	**0.002**	**0.018**	0.382	0.206	0.591	0.459	0.688
TP, g/L	**0.0001**	0.155	0.773	0.345	0.811	0.310	**0.041**
ALB, g/L	**0.002**	0.581	0.693	0.061	0.937	0.529	0.276
IgG, g/L	0.112	0.699	0.329	0.612	0.660	0.299	0.442
T3, nmol/L	**0.001**	0.120	0.522	0.038	0.909	0.994	0.949
T4, µ d/L	**0.003**	0.004	0.331	0.041	**0.008**	**0.033**	0.685
GLU, nmol/L	0.633	0.716	0.660	0.458	0.300	0.633	0.745
IP, mmol/L	**0.001**	0.179	0.878	0.658	0.851	0.349	0.993
Mg, mmol/L	0.675	0.487	**0.060**	0.655	0.867	0.250	0.422
K	0.885	**0.041**	0.203	0.153	**0.007**	0.462	0.902
Na	**0.0001**	0.548	**0.039**	0.108	0.464	0.548	0.388
Fe, µmol/L	**0.001**	**0.001**	0.694	**0.008**	0.370	0.830	0.267
Ca, nmol/L	**0.001**	0.985	0.176	0.379	0.657	0.816	0.313
B12, pmol/L	0.158	**0.008**	0.158	0.055	0.121	**0.003**	0.934
CREA, µmol/L	0.069	0.831	0.406	0.222	0.463	0.864	0.660
AP, U/L	**0.027**	0.490	0.256	0.521	0.521	0.966	0.588
Urea, mmol/L	0.164	0.797	0.899	0.286	0.075	**0.014**	0.456
TSH	0.604	0.523	0.736	0.854	0.951	0.854	0.691
Total bilirubin (pmol/L)	0.338	0.280	0.280	0.290	0.290	0.349	0.338

AST—Aspartate aminotransferase, ALT—Alanine aminotransferase, Chol—Cholesterol, HDL-C—High-density lipoprotein cholesterol, LDL-C—Low-density lipoprotein cholesterol, TG—Triglycerides, TP—Total protein, ALB—Albumin, T3—Triiodothyronine, T4—Thyroxine, GLU—Glucose, IP—Phosphorus, Mg—Magnesium, K—Potassium, Na—Sodium, Fe—Iron, Ca—Calcium, CREA—Creatinine, AP—Alkaline phosphatase, TSH—Thyroid-stimulating hormone. TD—treatment duration, FF—fermented feed, V—vaccination. Bold values indicate significant differences (*p* ≤ 0.05).

**Table 5:** Influence of the analysed factors and their interactions on piglets’ growth performance.

**Piglets Growth Performance**	Average from day 25 to 61	**Treatments**			
		**S_nonV_**	**S_V_**	**RF_nonV_**	**RF_V_**
FCR		1.56	1.38	1.58	1.76
ADG		0.395	0.392	0.397	0.399
Tests of between-subjects effects: influence of analysed factors and their interactions on FCR and ADG					
**Dependent Variable**	**Fermented Feed**	**Vaccination**		**Fermented Feed × Vaccination**	
Significance (*p*)					
FCR	0.154	1.000		0.194	
ADG	0.962	0.996		0.979	
Differences among treatments (*p* < 0.05)					
**S_V_** **vs.** **S_nonV_**	**RF_V_** **vs.** **RF_nonV_**	**S_V_** **vs.** **RF_V_**	**S_nonV_** **vs.** **RF_nonV_**	**S_nonV_** **vs.** **RF_V_**	**RF_nonV_** **vs.** **S_V_**
ADG					
0.901	0.783	0.55	0.943	0.91	0.286
FCR					
0.001	0.002	0.0001	0.773	0.91	0.074

S_nonV_—non-vaccinated piglets fed with control group compound feed; S_V_—vaccinated piglets fed with control group compound feed; RF_nonV_—non-vaccinated piglets fed with fermented compound feed; RF_V_—vaccinated piglets fed with fermented compound feed. FCR—*feed conversion ratio*; ADG—average daily gain. Measurements were made at baseline—on day 25, before the start of the feeding experiment—and on day 61, the end of the experiment. Bold values indicate significant differences (*p* ≤ 0.05). Data are presented as mean values (*n* = 100 per group).

**Table 6:** Influence of the analysed factors on ammonia emission.

**Ammonia Emission**	**Day**	**Treatments**				
		**S_nonV_**	**S_V_**	**RF_nonV_**		**RF_V_**
	Baseline	0.837 ^A;a^	1.172 ^A;b^	1.584 ^A;a^		1.252 ^A;b^
	61	1.938 ^B;a^	1.063 ^A;b^	1.538 ^A;a^		0.810 ^B;b^
Tests of between-subject effects: influence of the analysed factors and their interactions on ammonia emission						
**Treatment Duration**	**Fermented Feed**	**Vaccination**	**Treatment Duration** **× Fermented Feed**	**Treatment Duration** **× Vaccination**	**Fermented Feed × Vaccination**	**Treatment Duration** **× Fermented Feed × Vaccination**
Significance (*p*)						
**0.0001**	**0.003**	**0.0001**	**0.0001**	**0.0001**	**0.0001**	**0.0001**

S_nonV_—non-vaccinated piglets fed with soya meal; S_V_—vaccinated piglets fed with soya meal; RF_nonV_—non-vaccinated piglets fed with fermented rapeseed meal; RF_V_—vaccinated piglets fed with fermented rapeseed meal. Measurements were done at baseline, on day 25 before the start of the feeding experiment, and on day 61, the end of the experiment. ^A,B^ Different uppercase letters indicate significant treatment duration-related differences (*p* ≤ 0.05). ^a,b^ Different lowercase letters indicate differences among treatments (*p* ≤ 0.05). Bold values indicate significant differences (*p* ≤ 0.05). Data are presented as the mean values (*n* = 3).

## Supplementary Materials

The following are available online at https://www.mdpi.com/2076-2615/10/7/1201/s1, Table S1: Species S_nonV_ group before experiment; Table S2: Species S_V_ group before experiment Species S_V_ group before experiment; Table S3: Species RF_nonV_ group before experiment; Table S4: Species RFV group before experiment; Table S5: Species S_nonV_ group after experiment; Table S6: Species SV group after experiment; Table S7: Species RF_nonV_ group after experiment; Table S8: Species RFV group after experiment; Table S9: Differences between microbiological parameters of the piglets’ faeces between all the tested groups; Table S10: Differences between blood parameters of the piglets between all the tested groups.
