# Supplementary material for: Influence of the Fermented Feed and Vaccination and Their Interaction on Parameters of Large White/Norwegian Landrace Piglets
Source: Animals (Basel). 2020 Jul 15;10(7):1201. doi: 10.3390/ani10071201 (PMC7401620; doi:10.3390/ani10071201)
Supplement: Supplementary file 1 [file animals-10-01201-s001.zip › Table S6 Species SV group after experiment.pdf]

Species SV group after experiment	Number of reads	Relative abundance
Prevotella copri	8047	20.23%
Lactobacillus amylovorus	6223	15.65%
Barnesiella intestinihominis	1835	4.61%
Faecalibacterium prausnitzii	1391	3.5%
Megasphaera elsdenii	1183	2.97%
Collinsella aerofaciens	1060	2.67%
Prevotella brevis	995	2.5%
Clostridium cellulovorans	969	2.44%
Prevotella stercorea	962	2.42%
Unclassified	938	2.36%
Gemmiger formicilis	897	2.26%
Terrisporobacter glycolicus	797	2%
Olsenella scatoligenes	524	1.32%
Denitrobacterium detoxificans	511	1.28%
Eubacterium rectale	492	1.24%
Oscillospira guilliermondii	460	1.16%
Prevotella oris	446	1.12%
Roseburia faecis	422	1.06%
Oscillibacter ruminantium	382	0.96%
Prevotella maculosa	345	0.87%
Olsenella umbonata	337	0.85%
Enorma massiliensis	318	0.8%
Flintibacter butyricus	298	0.75%
Anaerovibrio lipolyticus	287	0.72%
Dialister succinatiphilus	274	0.69%
Enterorhabdus mucosicola	231	0.58%
Sporobacter termitidis	230	0.58%
Alloprevotella rava	224	0.56%
Olsenella uli	214	0.54%
Prevotella oralis	212	0.53%
Senegalimassilia anaerobia	206	0.52%
Butyricicoccus pullicaecorum	203	0.51%
Fournierella massiliensis	191	0.48%
Intestinimonas butyriciproducens	188	0.47%
Blautia wexlerae	180	0.45%
Lactobacillus reuteri	179	0.45%
Catenibacterium mitsuokai	168	0.42%
Desulfovibrio piger	157	0.39%
Clostridium celatum	150	0.38%
unclassified Planctomycetales	146	0.37%
Oligosphaera ethanolica	127	0.32%
Prevotella dentalis	123	0.31%
Selenomonas bovis	121	0.3%
Lactobacillus pontis	114	0.29%
Olivibacter sitiensis	105	0.26%
Treponema succinifaciens	103	0.26%
Bacteroidales oral	102	0.26%
unclassified Tannerella	99	0.25%
Parvibacter caecicola	95	0.24%

Gracilibacter thermotolerans	94	0.24%
Prevotella histicola	94	0.24%
Intestinibacter bartlettii	89	0.22%
Ruminiclostridium thermocellum	87	0.22%
Lactobacillus delbrueckii	87	0.22%
Succinivibrio dextrinosolvens	86	0.22%
Romboutsia sedimentorum	85	0.21%
unclassified Bacteroidales	82	0.21%
unclassified Erysipelotrichaceae	81	0.2%
Parabacteroides distasonis	80	0.2%
Lactobacillus kitasatonis	80	0.2%
unclassified Barnesiella	79	0.2%
Fibrobacter intestinalis	77	0.19%
Eubacterium ramulus	75	0.19%
Lactobacillus jensenii	73	0.18%
Intestinimonas timonensis	71	0.18%
Prevotella buccae	69	0.17%
Olsenella profusa	66	0.17%
Lactobacillus crispatus	65	0.16%
Eubacterium coprostanoligenes	62	0.16%
unclassified Prevotella	58	0.15%
Intestinimonas massiliensis	57	0.14%
Fusicatenibacter saccharivorans	57	0.14%
Acetanaerobacterium elongatum	54	0.14%
Prevotella salivae	53	0.13%
Holdemanella biformis	52	0.13%
Paraeggerthella hongkongensis	52	0.13%
Acidaminobacter hydrogenoformans	52	0.13%
Slackia isoflavoniconvertens	52	0.13%
Solobacterium moorei	50	0.13%
Paraprevotella clara	50	0.13%
Lactobacillus panis	50	0.13%
Clostridium phoceensis	47	0.12%
Sutterella stercoricanis	47	0.12%
Ruminococcus faecis	47	0.12%
Blautia obeum	47	0.12%
Coprococcus catus	46	0.12%
Turicibacter sanguinis	45	0.11%
Phascolarctobacterium succinatutens	44	0.11%
Eubacterium desmolans	43	0.11%
Coprococcus comes	40	0.1%
Ruthenibacterium lactatiformans	39	0.1%
Blautia massiliensis	38	0.1%
unclassified Prevotellaceae	38	0.1%
Enterorhabdus caecimuris	37	0.09%
Erysipelothrix inopinata	37	0.09%
Desulfovibrio fairfieldensis	37	0.09%
Mitsuokella jalaludinii	36	0.09%
Eubacterium hallii	35	0.09%
Ruminococcus bromii	34	0.09%

Christensenella minuta	32	0.08%
Eubacterium ruminantium	32	0.08%
Eubacterium eligens	30	0.08%
Selenomonas ruminantium	30	0.08%
Prevotella ruminicola	29	0.07%
Lactobacillus helveticus	29	0.07%
Prevotella genomosp.	29	0.07%
Brassicibacter thermophilus	29	0.07%
Butyrivibrio fibrisolvens	28	0.07%
Adlercreutzia equolifaciens	28	0.07%
Roseburia inulinivorans	27	0.07%
Collinsella intestinalis	27	0.07%
cyanobacterium enrichment	26	0.07%
Clostridium cellobioparum	26	0.07%
Ruminococcus bicirculans	26	0.07%
unclassified Rikenella	26	0.07%
Pseudoflavonifractor capillosus	26	0.07%
Candidatus Soleaferrea	25	0.06%
Lutispora thermophila	24	0.06%
Geosporobacter ferrireducens	24	0.06%
Ruminococcus flavefaciens	24	0.06%
Clostridium quinii	24	0.06%
unclassified Clostridium	24	0.06%
Papillibacter cinnamivorans	23	0.06%
unclassified Lachnospiraceae	23	0.06%
Oscillibacter valericigenes	22	0.06%
Barnesiella viscericola	21	0.05%
Asaccharospora irregularis	21	0.05%
Dorea formicigenerans	21	0.05%
Prevotella paludivivens	20	0.05%
Dorea longicatena	20	0.05%
Lactobacillus frumenti	20	0.05%
Lactobacillus acidophilus	19	0.05%
Saccharofermentans acetigenes	19	0.05%
Prevotella conceptionensis	19	0.05%
Slackia exigua	19	0.05%
Blautia faecis	19	0.05%
unclassified Deltaproteobacteria	19	0.05%
Anaerobacterium chartisolvens	19	0.05%
Murimonas intestini	18	0.05%
Hungatella hathewayi	18	0.05%
Bifidobacterium pseudolongum	17	0.04%
Flavonifractor plautii	17	0.04%
Vallitalea pronyensis	17	0.04%
Clostridium amylolyticum	17	0.04%
unclassified Ruminococcaceae	16	0.04%
Oribacterium sinus	16	0.04%
Campylobacter lanienae	15	0.04%
unclassified Porphyromonadaceae	15	0.04%
Natranaerovirga pectinivora	15	0.04%

Clostridium aldenense	15	0.04%
Clostridium bovipellis	14	0.04%
unclassified Olsenella	14	0.04%
Parasutterella secunda	14	0.04%
unclassified Anaerovibrio	14	0.04%
Clostridium methylpentosum	13	0.03%
Ruminococcus torques	13	0.03%
Roseburia intestinalis	13	0.03%
Collinsella massiliensis	12	0.03%
unclassified Clostridia	12	0.03%
Succiniclasticum ruminis	12	0.03%
Peptococcus simiae	12	0.03%
Clostridium chartatabidum	12	0.03%
Roseburia hominis	12	0.03%
Prevotella denticola	12	0.03%
Collinsella stercoris	12	0.03%
Anaeromassilibacillus senegalensis	12	0.03%
Desulfovibrio desulfuricans	12	0.03%
Acetivibrio cellulolyticus	12	0.03%
Clostridium polysaccharolyticum	11	0.03%
Gorbachella massiliensis	11	0.03%
Anaerotaenia torta	11	0.03%
Intestinimonas gabonensis	11	0.03%
unclassified Paludibacter	11	0.03%
unclassified Clostridiales	11	0.03%
actinobacterium enrichment	11	0.03%
unclassified Chlamydiia	11	0.03%
Candidatus Heliomonas	10	0.03%
Methylocystis rosea	10	0.03%
Eubacterium siraeum	10	0.03%
unclassified Mollicutes	10	0.03%
Caloramator fervidus	10	0.03%
Eisenbergiella tayi	10	0.03%
Megasphaera hominis	10	0.03%
Clostridium leptum	10	0.03%
Acidaminococcus fermentans	10	0.03%
Clostridium cadaveris	10	0.03%
Clostridium oroticum	10	0.03%
Eubacterium oxidoreducens	10	0.03%
Clostridium disporicum	9	0.02%
Subdoligranulum variabile	9	0.02%
Selenomonas sputigena	9	0.02%
Bifidobacteriaceae genomosp.	9	0.02%
Bacteroidales genomosp.	9	0.02%
unclassified Bacteroidia	9	0.02%
Anaerostipes hadrus	9	0.02%
Blautia stercoris	9	0.02%
Clostridium cellulolyticum	9	0.02%
Clostridium xylanolyticum	9	0.02%
Sphaerochaeta coccoides	8	0.02%

Falcatimonas natans	8	0.02%
Clostridium chauvoei	8	0.02%
Blautia luti	8	0.02%
Slackia piriformis	8	0.02%
Clostridium sartagoforme	8	0.02%
Blautia glucerasea	8	0.02%
unclassified Turicibacter	8	0.02%
Anaerovorax odorimutans	8	0.02%
Lachnospira pectinoschiza	7	0.02%
Helicobacter rodentium	7	0.02%
Garciella nitratireducens	7	0.02%
Eubacteriaceae oral	7	0.02%
Clostridium intestinale	7	0.02%
Holdemania filiformis	7	0.02%
Prevotella dentasini	7	0.02%
Herbinix luporum	7	0.02%
Desulfotomaculum halophilum	7	0.02%
Mucispirillum schaedleri	7	0.02%
Prevotella baroniae	7	0.02%
Ruminococcus gnavus	7	0.02%
Faecalicoccus acidiformans	7	0.02%
Acetivibrio ethanolgignens	7	0.02%
Ruminococcus callidus	7	0.02%
Clostridium aurantibutyricum	6	0.02%
Eubacterium rangiferina	6	0.02%
Clostridium saccharolyticum	6	0.02%
Zhizhongheella caldifontis	6	0.02%
Bacteroides heparinolyticus	6	0.02%
Pseudomonas fluorescens	6	0.02%
Clostridium hiranonis	6	0.02%
Porphyromonas catoniae	6	0.02%
Lactobacillus mucosae	6	0.02%
Propionispira arcuata	6	0.02%
unclassified Wautersiella	6	0.02%
Lactobacillus gallinarum	6	0.02%
Ethanoligenens harbinense	6	0.02%
Clostridium hveragerdense	6	0.02%
Treponema porcinum	6	0.02%
Clostridium clariflavum	6	0.02%
Mitsuokella multacida	5	0.01%
Abyssivirga alkaniphila	5	0.01%
unclassified Spirochaetia	5	0.01%
Eubacterium plexicaudatum	5	0.01%
Eubacterium infirmum	5	0.01%
Terrisporobacter petrolearius	5	0.01%
unclassified Eubacterium	5	0.01%
Clostridium tertium	5	0.01%
Prevotella loescheii	5	0.01%
Coprococcus eutactus	5	0.01%
Alloprevotella tannerae	5	0.01%

Ruminobacter amylophilus	5	0.01%
Elbe River	5	0.01%
unclassified Bulleidia	5	0.01%
Clostridium clostridioforme	5	0.01%
Propionispira paucivorans	5	0.01%
Bacteroides vulgatus	5	0.01%
Clostridium papyrosolvens	5	0.01%
unclassified Enterococcus	5	0.01%
Holdemania massiliensis	4	0.01%
Prevotella buccalis	4	0.01%
Parabacteroides chinchillae	4	0.01%
Asteroleplasma anaerobium	4	0.01%
Bacteroides zoogleoformans	4	0.01%
Clostridium hungatei	4	0.01%
Candidatus Dorea	4	0.01%
Parabacteroides merdae	4	0.01%
Clostridium viride	4	0.01%
Corynebacterium provencense	4	0.01%
Clostridium butyricum	4	0.01%
unclassified Oscillospira	4	0.01%
Ruminococcus champanellensis	4	0.01%
unclassified Actinobacteria	4	0.01%
Clostridium lavalense	4	0.01%
Clostridium aminobutyricum	4	0.01%
Faecalitalea cylindroides	4	0.01%
Mogibacterium diversum	4	0.01%
Prevotella fusca	4	0.01%
Acidaminococcus intestini	4	0.01%
Caminicella sporogenes	4	0.01%
Clostridium isatidis	4	0.01%
Catabacter hongkongensis	4	0.01%
Escherichia coli	4	0.01%
Lactobacillus amylolyticus	4	0.01%
Paeniclostridium sordellii	4	0.01%
unclassified Roseburia	4	0.01%
Dialister propionicifaciens	4	0.01%
Butyrivibrio crossotus	4	0.01%
Caproiciproducens galactitolivorans	4	0.01%
Ruminococcus albus	3	0.01%
Eggerthella lenta	3	0.01%
Treponema zioleckii	3	0.01%
Bacteroides ovatus	3	0.01%
Clostridium longisporum	3	0.01%
Prevotella timonensis	3	0.01%
unclassified Oscillibacter	3	0.01%
unclassified Candidatus Solibacter	3	0.01%
Allisonella histaminiformans	3	0.01%
Bacteroides nordii	3	0.01%
unclassified Faecalibacterium	3	0.01%
unclassified Coriobacteriaceae	3	0.01%

Agathobacter ruminis	3	0.01%
Prevotella shahii	3	0.01%
Clostridium fusiformis	3	0.01%
Sutterella parvirubra	3	0.01%
Lactobacillus vaginalis	3	0.01%
Lactonifactor longoviformis	3	0.01%
Hespellia porcina	3	0.01%
unclassified Dialister	3	0.01%
Eubacterium contortum	3	0.01%
Sphaerochaeta pleomorpha	3	0.01%
Bacteroides caecicola	3	0.01%
Clostridium populeti	3	0.01%
Clostridium fimetarium	3	0.01%
Robinsoniella peoriensis	3	0.01%
Desulfotomaculum tongense	3	0.01%
Lactobacillus psittaci	3	0.01%
Clostridium colicanis	3	0.01%
Bifidobacterium boum	3	0.01%
Streptococcus gallolyticus	3	0.01%
Bacteroides uniformis	3	0.01%
Bacteroides helcogenes	3	0.01%
unclassified Candidatus Glomeribacter	3	0.01%
Oceanirhabdus sediminicola	3	0.01%
Clostridium moniliforme	3	0.01%
Lactobacillus johnsonii	3	0.01%
alpha proteobacterium	3	0.01%
Natronincola histidinovorans	3	0.01%
Clostridium celerecrescens	2	0.01%
Porphyromonas cangingivalis	2	0.01%
Paludibacter jiangxiensis	2	0.01%
Lentzea violacea	2	0.01%
metal-contaminated soil	2	0.01%
unclassified Bacteroides	2	0.01%
Pseudomonas lurida	2	0.01%
Bifidobacterium kashiwanohense	2	0.01%
Clostridioides difficile	2	0.01%
Coriobacterium glomerans	2	0.01%
Bifidobacterium choerinum	2	0.01%
Bacteroides clarus	2	0.01%
Clostridium glycyrrhizinilyticum	2	0.01%
unclassified Collinsella	2	0.01%
Actinomyces denticolens	2	0.01%
Prevotella jejuni	2	0.01%
Prevotella corporis	2	0.01%
Clostridium stercorarium	2	0.01%
Eubacterium tenue	2	0.01%
Bifidobacterium breve	2	0.01%
Blautia schinkii	2	0.01%
Lactobacillus hamsteri	2	0.01%
Lactobacillus fermentum	2	0.01%

Moorella humiferrea	2	0.01%
Bacteroides cellulosilyticus	2	0.01%
Anaerosporobacter mobilis	2	0.01%
unclassified Clostridiaceae	2	0.01%
Thermotalea metallivorans	2	0.01%
Collinsella tanakaei	2	0.01%
Bacteroides stercoris	2	0.01%
Terrisporobacter mayombei	2	0.01%
Cellulosibacter alkalithermophilus	2	0.01%
Geosporobacter subterraneus	2	0.01%
Mageeibacillus indolicus	2	0.01%
unclassified Solobacterium	2	0.01%
Clostridium aminophilum	2	0.01%
Prevotella bryantii	2	0.01%
Anaerostipes butyraticus	2	0.01%
Laceyella putida	2	0.01%
Hallella seregens	2	0.01%
gamma proteobacterium	2	0.01%
unclassified Acetivibrio	2	0.01%
Bacteroides barnesiae	2	0.01%
Lachnoanaerobaculum umeaense	2	0.01%
Campylobacter jejuni	2	0.01%
Bifidobacterium animalis	2	0.01%
Eisenbergiella massiliensis	2	0.01%
Prevotella bivia	2	0.01%
Treponema brennaborense	2	0.01%
Bacteroides fragilis	2	0.01%
Clostridium straminisolvens	2	0.01%
unclassified Acidobacterium	2	0.01%
unclassified Treponema	2	0.01%
Clostridium asparagiforme	2	0.01%
unclassified Veillonellaceae	2	0.01%
unclassified Sutterella	2	0.01%
Marvinbryantia formatexigens	2	0.01%
Clostridium tarantellae	2	0.01%
Defluviitalea raffinosedens	2	0.01%
Anaerocolumna cellulosilytica	2	0.01%
unclassified Lactobacillus	2	0.01%
Blautia hansenii	1	0%
Prevotella albensis	1	0%
Methylocystis echinoides	1	0%
Lachnospiraceae oral	1	0%
unclassified Subdoligranulum	1	0%
Nakamurella panacisegetis	1	0%
Roseburia cecicola	1	0%
Leifsonia kafniensis	1	0%
Anaerocolumna aminovalerica	1	0%
Slackia faecicanis	1	0%
Clostridium taeniosporum	1	0%
Gordonibacter urolithinfaciens	1	0%

Megasphaera genomosp.	1	0%
Gottschalkia acidurici	1	0%
Atopobium vaginae	1	0%
Bacteroides faecichinchillae	1	0%
Eubacterium pyruvativorans	1	0%
Pleomorphochaeta multiformis	1	0%
Tyzzerella nexilis	1	0%
Nostocoida limicola	1	0%
Catenisphaera adipataccumulans	1	0%
Parabacteroides johnsonii	1	0%
Blautia producta	1	0%
Sphingopyxis panaciterrae	1	0%
Streptococcus danieliae	1	0%
Bacteroides caecigallinarum	1	0%
Mycobacterium tuberculosis	1	0%
proteobacterium enrichment	1	0%
Treponema berlinense	1	0%
Ercella succinigenes	1	0%
Peptoclostridium litorale	1	0%
Oligotropha carboxidovorans	1	0%
unclassified Spirochaeta	1	0%
Hespellia stercorisuis	1	0%
Thermanaerovibrio velox	1	0%
Streptomyces scabrisporus	1	0%
Bacteroides dorei	1	0%
Prevotella oulorum	1	0%
Butyrivibrio cf.	1	0%
Prevotella amnii	1	0%
Slackia equolifaciens	1	0%
Byssovorax cruenta	1	0%
Lactobacillus rodentium	1	0%
Tindallia magadiensis	1	0%
Parasporobacterium paucivorans	1	0%
methanogenic archaeon	1	0%
Megasphaera indica	1	0%
Prevotella scopos	1	0%
unclassified Victivallaceae	1	0%
Bacteroides timonensis	1	0%
unclassified Catonella	1	0%
unclassified Beggiatoa	1	0%
Bacteroides salanitronis	1	0%
Paludibaculum fermentans	1	0%
Pseudobutyrivibrio ruminis	1	0%
Clostridium indolis	1	0%
Bacteroides salyersiae	1	0%
Corynebacterium variabile	1	0%
Bacillus pumilus	1	0%
Clostridium polyendosporum	1	0%
unclassified Staphylococcus	1	0%
Clostridium amygdalinum	1	0%

unclassified Candidatus Rhabdochlamydia	1	0%
Bacteroides coprophilus	1	0%
unclassified Rhodoferax	1	0%
Pedosphaera parvula	1	0%
Desulfotomaculum nigrificans	1	0%
Prevotella multiformis	1	0%
Desulfovibrio multispirans	1	0%
Lactobacillus gastricus	1	0%
Bacteroides luti	1	0%
Clostridium botulinum	1	0%
Streptococcus macedonicus	1	0%
Bifidobacterium longum	1	0%
Stomatobaculum longum	1	0%
Nitrospirillum amazonense	1	0%
Clostridium sufflavum	1	0%
unclassified Alloprevotella	1	0%
Mobilitalea sibirica	1	0%
Marinilabilia salmonicolor	1	0%
Anaeroplasma bactoclasticum	1	0%
Clostridium elmenteitii	1	0%
Clostridium tyrobutyricum	1	0%
Anaerofilum pentosovorans	1	0%
Ruminococcus gauvreauii	1	0%
beta proteobacterium	1	0%
Novosphingobium sediminicola	1	0%
Caloramator australicus	1	0%
CFB group	1	0%
Stenotrophobacter terrae	1	0%
unclassified Achromobacter	1	0%
Lactobacillus camelliae	1	0%
Aeriscardovia aeriphila	1	0%
Eubacterium cellulosolvens	1	0%
Casaltella massiliensis	1	0%
Janthinobacterium agaricidamnosum	1	0%
Rhodoplanes roseus	1	0%
Schlegelella aquatica	1	0%
Anaerotruncus colihominis	1	0%
Desulfomonas oviles	1	0%
Streptococcus lutetiensis	1	0%
unclassified Bifidobacterium	1	0%
Hathewaya histolytica	1	0%
Actinocorallia cavernae	1	0%
Clostridium frigoriphilum	1	0%
unclassified Gordonibacter	1	0%
Bulleidia extructa	1	0%
Clostridium symbiosum	1	0%
unclassified Alphaproteobacteria	1	0%
Clostridium bornimense	1	0%
Lactobacillus secaliphilus	1	0%
Lachnospira multipara	1	0%

Alistipes senegalensis	1	0%
Prevotella micans	1	0%
unclassified Kopriimonas	1	0%
Bifidobacterium saguini	1	0%
Eubacterium limosum	1	0%
Ruminococcus lactaris	1	0%
Methanosphaera cuniculi	1	0%
Breznakia pachnodae	1	0%
Acetatifactor muris	1	0%
Clostridium lactatifermentans	1	0%
Tannerella forsythia	1	0%
Bacteroides acidifaciens	1	0%
Tetracoccus cechii	1	0%
Propionispira arboris	1	0%
Eubacterium saphenum	1	0%
Conexibacter woesei	1	0%
Vitis hybrid	1	0%
unclassified Desulfovibrio	1	0%
Eggerthella sinensis	1	0%
Catonella morbi	1	0%
Pseudomonas putida	1	0%
Actinomyces naeslundii	1	0%
Clostridium sulfidigenes	1	0%
Bariatricus massiliensis	1	0%
unclassified Sphingobacterium	1	0%
Methyloceanibacter caenitepidi	1	0%
Rarimicrobium hominis	1	0%
Lachnoanaerobaculum cf.	1	0%
Parabacteroides faecis	1	0%
Eubacterium xylanophilum	1	0%
Paraclostridium benzoelyticum	1	0%
unclassified Erysipelotrichia	1	0%
unclassified Sporobacter	1	0%
Centipeda periodontii	1	0%
Methylosinus sporium	1	0%
Pediococcus inopinatus	1	0%
Lactobacillus gigeriorum	1	0%
Clostridium alkalicellulosi	1	0%
Rhodanobacter spathiphylli	1	0%
Helicobacter canadensis	1	0%
Clostridium septicum	1	0%
Parabacteroides goldsteinii	1	0%
