# Supplementary material for: Influence of the Fermented Feed and Vaccination and Their Interaction on Parameters of Large White/Norwegian Landrace Piglets
Source: Animals (Basel). 2020 Jul 15;10(7):1201. doi: 10.3390/ani10071201 (PMC7401620; doi:10.3390/ani10071201)
Supplement: Supplementary file 1 [file animals-10-01201-s001.zip › Table S7 Species RFnonV group after experiment.pdf]

| Species RFnonV group after experiment | Number of reads | Relative abundance |
|---|---|---|
| Lactobacillus amylovorus | 11170 | 29.45% |
| Prevotella copri | 9558 | 25.2% |
| Megasphaera elsdenii | 1121 | 2.96% |
| Faecalibacterium prausnitzii | 1069 | 2.82% |
| Unclassified | 989 | 2.61% |
| Prevotella stercorea | 877 | 2.31% |
| Collinsella aerofaciens | 841 | 2.22% |
| Gemmiger formicilis | 614 | 1.62% |
| Barnesiella intestinihominis | 570 | 1.5% |
| Alloprevotella rava | 412 | 1.09% |
| Lactobacillus crispatus | 402 | 1.06% |
| Prevotella oris | 363 | 0.96% |
| Anaerovibrio lipolyticus | 351 | 0.93% |
| Denitrobacterium detoxificans | 347 | 0.91% |
| Enorma massiliensis | 311 | 0.82% |
| Enterorhabdus mucosicola | 304 | 0.8% |
| Prevotella oralis | 304 | 0.8% |
| Lactobacillus panis | 261 | 0.69% |
| Prevotella brevis | 249 | 0.66% |
| Lactobacillus kitasatonis | 231 | 0.61% |
| Olsenella scatoligenes | 226 | 0.6% |
| Roseburia faecis | 215 | 0.57% |
| Oscillospira guilliermondii | 202 | 0.53% |
| Blautia wexlerae | 189 | 0.5% |
| Lactobacillus pontis | 174 | 0.46% |
| Bacteroides caccae | 170 | 0.45% |
| Flintibacter butyricus | 159 | 0.42% |
| Sporobacter termitidis | 155 | 0.41% |
| unclassified Bacteroidales | 155 | 0.41% |
| Escherichia coli | 149 | 0.39% |
| Phascolarctobacterium succinatutens | 148 | 0.39% |
| Eubacterium rectale | 117 | 0.31% |
| Prevotella dentalis | 115 | 0.3% |
| Oscillibacter ruminantium | 112 | 0.3% |
| Lactobacillus delbrueckii | 104 | 0.27% |
| Fusicatenibacter saccharivorans | 102 | 0.27% |
| Butyricicoccus pullicaecorum | 98 | 0.26% |
| Eubacterium ramulus | 86 | 0.23% |
| Paraprevotella clara | 85 | 0.22% |
| Intestinimonas butyriciproducens | 83 | 0.22% |
| Catenibacterium mitsuokai | 81 | 0.21% |
| Bacteroidales oral | 79 | 0.21% |
| Parabacteroides distasonis | 76 | 0.2% |
| Lactobacillus jensenii | 74 | 0.2% |
| Coprococcus catus | 73 | 0.19% |
| Holdemanella biformis | 69 | 0.18% |
| Eubacterium desmolans | 68 | 0.18% |
| Blautia massiliensis | 66 | 0.17% |
| Olsenella uli | 66 | 0.17% |

| | | |
|---|---|---|
| Prevotella salivae | 64 | 0.17% |
| Desulfovibrio fairfieldensis | 64 | 0.17% |
| Prevotella buccae | 62 | 0.16% |
| Eubacterium coprostanoligenes | 60 | 0.16% |
| Treponema succinifaciens | 60 | 0.16% |
| Oligosphaera ethanolica | 60 | 0.16% |
| Blautia producta | 55 | 0.15% |
| unclassified Barnesiella | 55 | 0.15% |
| Ruminococcus flavefaciens | 54 | 0.14% |
| Prevotella genomosp. | 54 | 0.14% |
| Sutterella stercoricanis | 54 | 0.14% |
| Lactobacillus helveticus | 53 | 0.14% |
| Acidaminobacter hydrogenoformans | 52 | 0.14% |
| Parvibacter caecicola | 50 | 0.13% |
| unclassified Prevotella | 48 | 0.13% |
| Prevotella conceptionensis | 48 | 0.13% |
| Fournierella massiliensis | 45 | 0.12% |
| Blautia obeum | 44 | 0.12% |
| Olsenella umbonata | 44 | 0.12% |
| Enterorhabdus caecimuris | 43 | 0.11% |
| Clostridium phoceensis | 41 | 0.11% |
| Roseburia inulinivorans | 41 | 0.11% |
| Intestinibacter bartlettii | 41 | 0.11% |
| Ruminococcus faecis | 41 | 0.11% |
| Lactobacillus frumenti | 41 | 0.11% |
| Lactobacillus acidophilus | 40 | 0.11% |
| Solobacterium moorei | 40 | 0.11% |
| unclassified Tannerella | 40 | 0.11% |
| Coprococcus comes | 39 | 0.1% |
| Gracilibacter thermotolerans | 39 | 0.1% |
| Caloramator fervidus | 38 | 0.1% |
| Eubacterium ruminantium | 37 | 0.1% |
| Desulfovibrio piger | 37 | 0.1% |
| Roseburia hominis | 36 | 0.09% |
| Ruminococcus torques | 36 | 0.09% |
| Murimonas intestini | 36 | 0.09% |
| Eubacterium eligens | 35 | 0.09% |
| Dorea formicigenerans | 35 | 0.09% |
| Dorea longicatena | 35 | 0.09% |
| cyanobacterium enrichment | 32 | 0.08% |
| Intestinimonas timonensis | 32 | 0.08% |
| unclassified Lachnospiraceae | 31 | 0.08% |
| Collinsella intestinalis | 31 | 0.08% |
| Blautia stercoris | 30 | 0.08% |
| Ruthenibacterium lactatiformans | 29 | 0.08% |
| unclassified Alloprevotella | 29 | 0.08% |
| Bifidobacteriaceae genomosp. | 28 | 0.07% |
| Clostridium aldenense | 27 | 0.07% |
| Terrisporobacter glycolicus | 27 | 0.07% |
| unclassified Erysipelotrichaceae | 27 | 0.07% |

| | | |
|---|---|---|
| Intestinimonas massiliensis | 26 | 0.07% |
| Anaerotaenia torta | 26 | 0.07% |
| Eubacterium hallii | 26 | 0.07% |
| unclassified Planctomycetales | 25 | 0.07% |
| Ruminiclostridium thermocellum | 25 | 0.07% |
| Slackia isoflavoniconvertens | 23 | 0.06% |
| Lactobacillus reuteri | 22 | 0.06% |
| Acetivibrio ethanolgignens | 22 | 0.06% |
| Lachnospira pectinoschiza | 22 | 0.06% |
| Prevotella ruminicola | 21 | 0.06% |
| Blautia faecis | 21 | 0.06% |
| unclassified Turicibacter | 21 | 0.06% |
| Adlercreutzia equolifaciens | 21 | 0.06% |
| Clostridium aminobutyricum | 21 | 0.06% |
| Prevotella histicola | 20 | 0.05% |
| unclassified Prevotellaceae | 20 | 0.05% |
| Prevotella loescheii | 19 | 0.05% |
| Anaerobacterium chartisolvens | 19 | 0.05% |
| Blautia luti | 18 | 0.05% |
| Olsenella profusa | 18 | 0.05% |
| Prevotella maculosa | 18 | 0.05% |
| Senegalimassilia anaerobia | 17 | 0.04% |
| Mitsuokella jalaludinii | 17 | 0.04% |
| Paraeggerthella hongkongensis | 17 | 0.04% |
| Candidatus Soleaferrea | 17 | 0.04% |
| Ruminococcus callidus | 17 | 0.04% |
| Vallitalea pronyensis | 16 | 0.04% |
| Collinsella massiliensis | 16 | 0.04% |
| Acetanaerobacterium elongatum | 16 | 0.04% |
| Hungatella hathewayi | 16 | 0.04% |
| Ruminococcus bicirculans | 15 | 0.04% |
| Clostridium saccharolyticum | 15 | 0.04% |
| unclassified Clostridiales | 15 | 0.04% |
| Succinivibrio dextrinosolvens | 15 | 0.04% |
| Hallella seregens | 14 | 0.04% |
| unclassified Bacteroides | 14 | 0.04% |
| unclassified Rikenella | 14 | 0.04% |
| Eubacterium siraeum | 14 | 0.04% |
| Barnesiella viscericola | 13 | 0.03% |
| unclassified Candidatus Glomeribacter | 13 | 0.03% |
| Clostridium populeti | 13 | 0.03% |
| Clostridium fusiformis | 13 | 0.03% |
| Christensenella minuta | 13 | 0.03% |
| Helicobacter rodentium | 13 | 0.03% |
| Herbinix luporum | 12 | 0.03% |
| Oribacterium sinus | 12 | 0.03% |
| Ruminococcus bromii | 11 | 0.03% |
| Prevotella shahii | 11 | 0.03% |
| Prevotella bivia | 11 | 0.03% |
| Butyrivibrio fibrisolvens | 11 | 0.03% |

| | | |
|---|---|---|
| Erysipelothrix inopinata | 11 | 0.03% |
| unclassified Anaerovibrio | 11 | 0.03% |
| Oscillibacter valericigenes | 11 | 0.03% |
| Clostridium cellulovorans | 11 | 0.03% |
| Clostridium xylanolyticum | 10 | 0.03% |
| Alloprevotella tannerae | 10 | 0.03% |
| Clostridium celatum | 10 | 0.03% |
| Prevotella dentasini | 10 | 0.03% |
| Fibrobacter intestinalis | 10 | 0.03% |
| Anaerostipes butyraticus | 10 | 0.03% |
| Campylobacter lanienae | 10 | 0.03% |
| Porphyromonas catoniae | 9 | 0.02% |
| Eubacteriaceae oral | 9 | 0.02% |
| Clostridium polysaccharolyticum | 9 | 0.02% |
| Holdemania filiformis | 9 | 0.02% |
| unclassified Porphyromonadaceae | 9 | 0.02% |
| Roseburia intestinalis | 9 | 0.02% |
| Abyssivirga alkaniphila | 9 | 0.02% |
| Clostridium cellobioparum | 9 | 0.02% |
| Megasphaera hominis | 9 | 0.02% |
| Hespellia porcina | 9 | 0.02% |
| Parasutterella secunda | 9 | 0.02% |
| Slackia piriformis | 9 | 0.02% |
| Ruminococcus lactaris | 9 | 0.02% |
| Propionispira arcuata | 8 | 0.02% |
| Olivibacter sitiensis | 8 | 0.02% |
| Mucispirillum schaedleri | 8 | 0.02% |
| Collinsella stercoris | 8 | 0.02% |
| Lactobacillus secaliphilus | 8 | 0.02% |
| Methylocystis rosea | 8 | 0.02% |
| Mitsuokella multacida | 8 | 0.02% |
| Pseudoflavonifractor capillosus | 8 | 0.02% |
| Anaerostipes hadrus | 8 | 0.02% |
| Candidatus Dorea | 8 | 0.02% |
| unclassified Eubacterium | 8 | 0.02% |
| Eggerthella lenta | 8 | 0.02% |
| Geosporobacter ferrireducens | 8 | 0.02% |
| Elbe River | 7 | 0.02% |
| Falcatimonas natans | 7 | 0.02% |
| Robinsoniella peoriensis | 7 | 0.02% |
| Natranaerovirga pectinivora | 7 | 0.02% |
| Clostridium oroticum | 7 | 0.02% |
| Parabacteroides goldsteinii | 7 | 0.02% |
| Clostridium leptum | 7 | 0.02% |
| Subdoligranulum variabile | 7 | 0.02% |
| Selenomonas bovis | 7 | 0.02% |
| Agathobacter ruminis | 7 | 0.02% |
| Bacteroidales genomosp. | 7 | 0.02% |
| Bacteroides intestinalis | 7 | 0.02% |
| Bacteroides barnesiae | 7 | 0.02% |

| | | |
|---|---|---|
| Peptococcus simiae | 7 | 0.02% |
| Turicibacter sanguinis | 7 | 0.02% |
| Mobilitalea sibirica | 6 | 0.02% |
| Coprococcus eutactus | 6 | 0.02% |
| Bacteroides uniformis | 6 | 0.02% |
| Marvinbryantia formatexigens | 6 | 0.02% |
| Paludibacter jiangxiensis | 6 | 0.02% |
| unclassified Ruminococcaceae | 6 | 0.02% |
| Brassicibacter thermophilus | 6 | 0.02% |
| Blautia glucerasea | 6 | 0.02% |
| Garciella nitratireducens | 6 | 0.02% |
| Clostridium asparagiforme | 6 | 0.02% |
| Anaerobium acetethylicum | 6 | 0.02% |
| Clostridium clariflavum | 6 | 0.02% |
| Zhizhongheella caldifontis | 6 | 0.02% |
| unclassified Clostridia | 5 | 0.01% |
| Clostridium lactatifermentans | 5 | 0.01% |
| Lactobacillus rogosae | 5 | 0.01% |
| Bacteroides stercoris | 5 | 0.01% |
| unclassified Mollicutes | 5 | 0.01% |
| unclassified Deltaproteobacteria | 5 | 0.01% |
| Ruminococcus gnavus | 5 | 0.01% |
| Caminicella sporogenes | 5 | 0.01% |
| unclassified Faecalibacterium | 5 | 0.01% |
| Lactobacillus coleohominis | 5 | 0.01% |
| Slackia exigua | 5 | 0.01% |
| unclassified Acetivibrio | 5 | 0.01% |
| unclassified Lactobacillus | 5 | 0.01% |
| Desulfotomaculum tongense | 5 | 0.01% |
| Thermotalea metallivorans | 5 | 0.01% |
| Lutispora thermophila | 5 | 0.01% |
| Bacteroides pectinophilus | 5 | 0.01% |
| Selenomonas ruminantium | 5 | 0.01% |
| Gorbachella massiliensis | 5 | 0.01% |
| Papillibacter cinnamivorans | 5 | 0.01% |
| Eubacterium oxidoreducens | 5 | 0.01% |
| Anaerovorax odorimutans | 5 | 0.01% |
| Blautia schinkii | 5 | 0.01% |
| Prevotella bryantii | 4 | 0.01% |
| Sphaerochaeta coccoides | 4 | 0.01% |
| Prevotella enoeca | 4 | 0.01% |
| Flavonifractor plautii | 4 | 0.01% |
| Mogibacterium diversum | 4 | 0.01% |
| Clostridium clostridioforme | 4 | 0.01% |
| Clostridium cellulolyticum | 4 | 0.01% |
| Prevotella buccalis | 4 | 0.01% |
| Eubacterium rangiferina | 4 | 0.01% |
| Desulfotomaculum halophilum | 4 | 0.01% |
| Prevotella fusca | 4 | 0.01% |
| Eubacterium plexicaudatum | 4 | 0.01% |

| | | |
|---|---|---|
| unclassified Clostridium | 4 | 0.01% |
| Treponema zioleckii | 4 | 0.01% |
| Romboutsia sedimentorum | 4 | 0.01% |
| Clostridium symbiosum | 4 | 0.01% |
| Faecalicoccus acidiformans | 4 | 0.01% |
| Lactobacillus hamsteri | 4 | 0.01% |
| Clostridium hiranonis | 3 | 0.01% |
| Bacteroides oleiciplenus | 3 | 0.01% |
| Bacteroides cellulosilyticus | 3 | 0.01% |
| Holdemania massiliensis | 3 | 0.01% |
| Parasporobacterium paucivorans | 3 | 0.01% |
| Corynebacterium provencense | 3 | 0.01% |
| Prevotella denticola | 3 | 0.01% |
| unclassified Coriobacteriaceae | 3 | 0.01% |
| Parabacteroides merdae | 3 | 0.01% |
| Eubacterium contortum | 3 | 0.01% |
| unclassified Petrimonas | 3 | 0.01% |
| Anaeromassilibacillus senegalensis | 3 | 0.01% |
| Bacteroides helcogenes | 3 | 0.01% |
| Bacteroides caecigallinarum | 3 | 0.01% |
| Candidatus Treponema | 3 | 0.01% |
| Bifidobacterium bohemicum | 3 | 0.01% |
| Parabacteroides chinchillae | 3 | 0.01% |
| Salmonella enterica | 3 | 0.01% |
| Oceanirhabdus sediminicola | 3 | 0.01% |
| unclassified Sphingobium | 3 | 0.01% |
| methanogenic archaeon | 3 | 0.01% |
| Candidatus Heliomonas | 3 | 0.01% |
| unclassified Collinsella | 3 | 0.01% |
| Desulfovibrio desulfuricans | 3 | 0.01% |
| Bacteroides timonensis | 3 | 0.01% |
| Prevotella micans | 3 | 0.01% |
| unclassified Bacillus | 3 | 0.01% |
| Caloranaerobacter azorensis | 3 | 0.01% |
| Prevotella paludivivens | 3 | 0.01% |
| unclassified Wautersiella | 3 | 0.01% |
| Clostridium lavalense | 3 | 0.01% |
| Kluyvera georgiana | 3 | 0.01% |
| Bacteroides nordii | 3 | 0.01% |
| Bacteroides vulgatus | 3 | 0.01% |
| Porphyromonas cangingivalis | 2 | 0.01% |
| unclassified Enterococcus | 2 | 0.01% |
| Selenomonas sputigena | 2 | 0.01% |
| Clostridium methylpentosum | 2 | 0.01% |
| Ruminococcus gauvreauii | 2 | 0.01% |
| Elusimicrobium minutum | 2 | 0.01% |
| Asteroleplasma anaerobium | 2 | 0.01% |
| Clostridium quinii | 2 | 0.01% |
| Prevotella albensis | 2 | 0.01% |
| Dielma fastidiosa | 2 | 0.01% |

| | | |
|---|---|---|
| unclassified Oscillibacter | 2 | 0.01% |
| Lactobacillus agilis | 2 | 0.01% |
| Acidaminococcus fermentans | 2 | 0.01% |
| Bacteroides salanitronis | 2 | 0.01% |
| Lachnospira multipara | 2 | 0.01% |
| metal-contaminated soil | 2 | 0.01% |
| Asaccharobacter celatus | 2 | 0.01% |
| Shigella dysenteriae | 2 | 0.01% |
| Hydrogenoanaerobacterium saccharovorans | 2 | 0.01% |
| Casaltella massiliensis | 2 | 0.01% |
| unclassified Oscillospira | 2 | 0.01% |
| Blautia hydrogenotrophica | 2 | 0.01% |
| Acetivibrio cellulolyticus | 2 | 0.01% |
| Anaerocolumna cellulosilytica | 2 | 0.01% |
| Clostridium sphenoides | 2 | 0.01% |
| Ethanoligenens harbinense | 2 | 0.01% |
| Catabacter hongkongensis | 2 | 0.01% |
| unclassified Dysgonomonas | 2 | 0.01% |
| Bacillus pumilus | 2 | 0.01% |
| alpha proteobacterium | 2 | 0.01% |
| unclassified Lactobacillaceae | 2 | 0.01% |
| Asaccharospora irregularis | 2 | 0.01% |
| actinobacterium enrichment | 2 | 0.01% |
| Saccharofermentans acetigenes | 2 | 0.01% |
| unclassified Erysipelotrichia | 2 | 0.01% |
| Eisenbergiella tayi | 2 | 0.01% |
| Eubacterium cellulosolvens | 2 | 0.01% |
| Bacteroides galacturonicus | 2 | 0.01% |
| Anaeroplasma abactoclasticum | 2 | 0.01% |
| Lachnoanaerobaculum umeaense | 2 | 0.01% |
| Blautia coccoides | 2 | 0.01% |
| unclassified Ruminococcus | 2 | 0.01% |
| Breznakia pachnodae | 2 | 0.01% |
| Hespellia stercorisuis | 2 | 0.01% |
| Clostridium chartatabidum | 2 | 0.01% |
| Bacteroides acidifaciens | 2 | 0.01% |
| Bacteroides coprocola | 2 | 0.01% |
| Lactobacillus amylolyticus | 2 | 0.01% |
| unclassified Olsenella | 2 | 0.01% |
| Propionispira paucivorans | 2 | 0.01% |
| Bacteroides heparinolyticus | 2 | 0.01% |
| Lachnoanaerobaculum cf. | 2 | 0.01% |
| Desulfotomaculum guttoideum | 2 | 0.01% |
| Lactobacillus gallinarum | 2 | 0.01% |
| unclassified Spirochaetia | 2 | 0.01% |
| Dialister succinatiphilus | 2 | 0.01% |
| Methylocystis echinoides | 2 | 0.01% |
| Porphyromonas pasteri | 2 | 0.01% |
| Collinsella tanakaei | 2 | 0.01% |
| Coprobacillus cateniformis | 2 | 0.01% |

| | | |
|---|---|---|
| Bacteroides ovatus | 2 | 0.01% |
| Laceyella putida | 2 | 0.01% |
| Treponema porcinum | 2 | 0.01% |
| Lacibacterium aquatile | 2 | 0.01% |
| Lachnoanaerobaculum orale | 2 | 0.01% |
| Pseudomonas fluorescens | 2 | 0.01% |
| unclassified Bulleidia | 2 | 0.01% |
| Treponema brennaborense | 2 | 0.01% |
| Corynebacterium variabile | 1 | 0% |
| Helicobacter equorum | 1 | 0% |
| Clostridium aerotolerans | 1 | 0% |
| Novispirillum itersonii | 1 | 0% |
| Thermovenabulum ferriorganovorum | 1 | 0% |
| Methylosinus sporium | 1 | 0% |
| Clostridiisalibacter paucivorans | 1 | 0% |
| Gemmatimonas aurantiaca | 1 | 0% |
| Meiothermus silvanus | 1 | 0% |
| Mangroviflexus xiamenensis | 1 | 0% |
| Dysgonomonas termitidis | 1 | 0% |
| Paludibacter propionicigenes | 1 | 0% |
| Methylopila jiangsuensis | 1 | 0% |
| Blautia hansenii | 1 | 0% |
| Magnetococcus marinus | 1 | 0% |
| Lactobacillus johnsonii | 1 | 0% |
| Paraprevotella xylaniphila | 1 | 0% |
| Caldicoprobacter algeriensis | 1 | 0% |
| Clostridium paradoxum | 1 | 0% |
| Odoribacter splanchnicus | 1 | 0% |
| green non-sulfur | 1 | 0% |
| Bacteroides faecis | 1 | 0% |
| Bifidobacterium pseudolongum | 1 | 0% |
| Peptococcus niger | 1 | 0% |
| Geosporobacter subterraneus | 1 | 0% |
| Actinoallomurus purpureus | 1 | 0% |
| Prevotella scopos | 1 | 0% |
| Sphingosinicella cucumeris | 1 | 0% |
| Eubacterium sulci | 1 | 0% |
| Allisonella histaminiformans | 1 | 0% |
| Ruminococcus albus | 1 | 0% |
| Romboutsia lituseburensis | 1 | 0% |
| Candidatus Stoquefichus | 1 | 0% |
| Lactobacillus porcinae | 1 | 0% |
| unclassified Kopriimonas | 1 | 0% |
| Pelotomaculum propionicicum | 1 | 0% |
| unclassified Megasphaera | 1 | 0% |
| unclassified Oribacterium | 1 | 0% |
| Akkermansia muciniphila | 1 | 0% |
| Clostridium celerecrescens | 1 | 0% |
| Planctomycete enrichment | 1 | 0% |
| Butyrivibrio hungatei | 1 | 0% |

| | | |
|---|---|---|
| Pseudobutyrivibrio ruminis | 1 | 0% |
| Actinoplanes sarveparensis | 1 | 0% |
| Acidaminococcus intestini | 1 | 0% |
| Coriobacterium glomerans | 1 | 0% |
| unclassified Actinobacteria | 1 | 0% |
| Acidisphaera rubrifaciens | 1 | 0% |
| Afipia broomeae | 1 | 0% |
| unclassified Porphyromonas | 1 | 0% |
| Lactivibrio alcoholicus | 1 | 0% |
| Clostridium aminophilum | 1 | 0% |
| Alkaliphilus metalliredigens | 1 | 0% |
| Thiohalobacter thiocyanaticus | 1 | 0% |
| Ruminococcus champanellensis | 1 | 0% |
| Stomatobaculum longum | 1 | 0% |
| Bacteroides dorei | 1 | 0% |
| Nocardioides hankookensis | 1 | 0% |
| Bacillus horikoshii | 1 | 0% |
| Devosia ginsengisoli | 1 | 0% |
| Desulfotomaculum nigrificans | 1 | 0% |
| Anaerofilum pentosovorans | 1 | 0% |
| beta proteobacterium | 1 | 0% |
| Alkalibaculum bacchi | 1 | 0% |
| Prevotella marshii | 1 | 0% |
| Sphingosinicella vermicomposti | 1 | 0% |
| Anaerostipes rhamnosivorans | 1 | 0% |
| Clostridium papyrosolvens | 1 | 0% |
| Helicobacter canadensis | 1 | 0% |
| unclassified Catenuloplanes | 1 | 0% |
| Bacillus asahii | 1 | 0% |
| Prevotella amnii | 1 | 0% |
| unclassified Clostridiaceae | 1 | 0% |
| Clostridium hylemonae | 1 | 0% |
| Anaerosporobacter mobilis | 1 | 0% |
| Kosakonia sacchari | 1 | 0% |
| Bacteroides gallinaceum | 1 | 0% |
| Tumebacillus ginsengisoli | 1 | 0% |
| Bacteroides salyersiae | 1 | 0% |
| Bariatricus massiliensis | 1 | 0% |
| unclassified Massilia | 1 | 0% |
| Defluviitalea raffinosedens | 1 | 0% |
| Bacteroides stercorirosoris | 1 | 0% |
| Bacteroides caecicola | 1 | 0% |
| Luteolibacter pohnpeiensis | 1 | 0% |
| Geoalkalibacter subterraneus | 1 | 0% |
| Sutterella wadsworthensis | 1 | 0% |
| Anaerobiospirillum succiniciproducens | 1 | 0% |
| Centipeda periodontii | 1 | 0% |
| Alkaliphilus crotonatoxidans | 1 | 0% |
| Mageeibacillus indolicus | 1 | 0% |
| Aeriscardovia aeriphila | 1 | 0% |

| | | |
|---|---|---|
| Pediococcus inopinatus | 1 | 0% |
| Bradyrhizobium genosp. | 1 | 0% |
| Lactobacillus diolivorans | 1 | 0% |
| Veillonella caviae | 1 | 0% |
| unclassified Verrucomicrobiales | 1 | 0% |
| type II | 1 | 0% |
| gamma proteobacterium | 1 | 0% |
| Prevotella aff. | 1 | 0% |
| Lachnoclostridium phytofermentans | 1 | 0% |
| Bacteroides massiliensis | 1 | 0% |
| Paraclostridium benzoelyticum | 1 | 0% |
| Campylobacter jejuni | 1 | 0% |
| Clostridium amylolyticum | 1 | 0% |
| Nesterenkonia aethiopica | 1 | 0% |
| Shigella flexneri | 1 | 0% |
| Bradyrhizobium jicamae | 1 | 0% |
| Ferruginibacter paludis | 1 | 0% |
| Eisenbergiella massiliensis | 1 | 0% |
| Pleomorphochaeta multiformis | 1 | 0% |
| Shigella boydii | 1 | 0% |
| Paenibacillus favisporus | 1 | 0% |
| Lachnobacterium bovis | 1 | 0% |
| Nakamurella lactea | 1 | 0% |
| unclassified Garciella | 1 | 0% |
| Anaerocolumna xylanovorans | 1 | 0% |
| Escherichia albertii | 1 | 0% |
| Porphyromonas pogonae | 1 | 0% |
| Atopobium rimae | 1 | 0% |
| Parabacteroides johnsonii | 1 | 0% |
| Cellulosibacter alkalithermophilus | 1 | 0% |
| Bacteroides paurosaccharolyticus | 1 | 0% |
| Methylosinus trichosporium | 1 | 0% |
| Lactobacillus hominis | 1 | 0% |
| Lactobacillus sunkii | 1 | 0% |
| Prevotella timonensis | 1 | 0% |
| Atopobium vaginae | 1 | 0% |
| Eubacterium uniforme | 1 | 0% |
| Dethiosulfatibacter aminovorans | 1 | 0% |
| Brooklawnia cerclae | 1 | 0% |
| Tyzzerella nexilis | 1 | 0% |
| Mycoplasma sualvi | 1 | 0% |
| Pseudobutyrivibrio xylanivorans | 1 | 0% |
| Prevotella bergensis | 1 | 0% |
| low G+C | 1 | 0% |
| Clostridium bolteae | 1 | 0% |
| unclassified Sutterella | 1 | 0% |
| Prevotella saccharolytica | 1 | 0% |
| unclassified Bacteroidaceae | 1 | 0% |
| Eubacterium fissicatena | 1 | 0% |
| Intestinimonas gabonensis | 1 | 0% |

| | | |
|---|---|---|
| Janthinobacterium lividum | 1 | 0% |
| Clostridium scindens | 1 | 0% |
| Clostridium hungatei | 1 | 0% |
| Rhodoferax saidenbachensis | 1 | 0% |
| Bacteroides plebeius | 1 | 0% |
| unclassified Alphaproteobacteria | 1 | 0% |
| Terrimonas arctica | 1 | 0% |
| Veillonella magna | 1 | 0% |
| Catonella morbi | 1 | 0% |
| Arenimonas oryziterrae | 1 | 0% |
| Bythopirellula goksoyri | 1 | 0% |
| Methylocystis heyeri | 1 | 0% |
| Clostridium chauvoei | 1 | 0% |
| Rhizorhabdus argentea | 1 | 0% |
| Defluviitalea saccharophila | 1 | 0% |
| Campylobacter coli | 1 | 0% |
| Nitrobacter vulgaris | 1 | 0% |
| Sphaerochaeta pleomorpha | 1 | 0% |
