# Supplementary material for: Influence of the Fermented Feed and Vaccination and Their Interaction on Parameters of Large White/Norwegian Landrace Piglets
Source: Animals (Basel). 2020 Jul 15;10(7):1201. doi: 10.3390/ani10071201 (PMC7401620; doi:10.3390/ani10071201)
Supplement: Supplementary file 1 [file animals-10-01201-s001.zip › Table S8 Species RFV group after experiment.pdf]

Species RFV group after experiment	Number of reads	Relative abundance
Lactobacillus amylovorus	16955	44.14%
Prevotella copri	7938	20.66%
Prevotella brevis	1774	4.62%
Unclassified	702	1.83%
Prevotella stercorea	615	1.6%
Megasphaera elsdenii	605	1.57%
Faecalibacterium prausnitzii	523	1.36%
Prevotella oris	389	1.01%
Eubacterium rectale	370	0.96%
Alloprevotella rava	336	0.87%
Lactobacillus crispatus	309	0.8%
Barnesiella intestinihominis	299	0.78%
Sporobacter termitidis	265	0.69%
Prevotella oralis	254	0.66%
Lactobacillus panis	253	0.66%
Roseburia faecis	234	0.61%
Lactobacillus kitasatonis	232	0.6%
Gemmiger formicilis	216	0.56%
Terrisporobacter glycolicus	195	0.51%
Lactobacillus pontis	182	0.47%
Lactobacillus delbrueckii	173	0.45%
Paraprevotella clara	147	0.38%
Prevotella dentalis	139	0.36%
Anaerovibrio lipolyticus	138	0.36%
Bacteroidales oral	132	0.34%
Ruminiclostridium thermocellum	118	0.31%
Butyricicoccus pullicaecorum	111	0.29%
Prevotella histicola	111	0.29%
Blautia wexlerae	106	0.28%
Coprococcus catus	99	0.26%
Phascolarctobacterium succinatutens	97	0.25%
Lactobacillus jensenii	94	0.24%
Coprococcus comes	91	0.24%
Oscillospira guilliermondii	88	0.23%
Anaerotaenia torta	86	0.22%
Flintibacter butyricus	81	0.21%
Oscillibacter ruminantium	75	0.2%
Prevotella ruminicola	74	0.19%
Eubacterium coprostanoligenes	73	0.19%
Eubacterium ruminantium	73	0.19%
Fusicatenibacter saccharivorans	73	0.19%
Clostridium cellulovorans	64	0.17%
Intestinibacter bartlettii	61	0.16%
Lactobacillus helveticus	59	0.15%
Murimonas intestini	59	0.15%
Clostridium celatum	58	0.15%
Lachnospira pectinoschiza	57	0.15%
Prevotella salivae	52	0.14%
Ruminococcus flavefaciens	51	0.13%

cyanobacterium enrichment	50	0.13%
Eubacterium eligens	49	0.13%
Lactobacillus frumenti	48	0.12%
Lactobacillus acidophilus	48	0.12%
unclassified Barnesiella	47	0.12%
Eubacterium hallii	45	0.12%
Eubacterium desmolans	45	0.12%
Intestinimonas butyriciproducens	44	0.11%
unclassified Bacteroidales	40	0.1%
Romboutsia sedimentorum	40	0.1%
Hungatella hathewayi	39	0.1%
Ruminococcus faecis	38	0.1%
unclassified Prevotellaceae	37	0.1%
unclassified Prevotella	36	0.09%
Parabacteroides distasonis	36	0.09%
Prevotella genomosp.	35	0.09%
Blautia obeum	33	0.09%
Ruminococcus torques	33	0.09%
Butyrivibrio fibrisolvens	32	0.08%
Ruminococcus bicirculans	32	0.08%
Blautia stercoris	32	0.08%
Solobacterium moorei	31	0.08%
Eubacterium ramulus	31	0.08%
Prevotella conceptionensis	30	0.08%
Blautia massiliensis	30	0.08%
Eubacteriaceae oral	27	0.07%
Holdemanella biformis	27	0.07%
Anaerobacterium chartisolvens	26	0.07%
Dorea formicigenerans	26	0.07%
Fournierella massiliensis	25	0.07%
Desulfovibrio piger	25	0.07%
Roseburia inulinivorans	25	0.07%
Dorea longicatena	25	0.07%
Succinivibrio dextrinosolvens	25	0.07%
Catenibacterium mitsuokai	25	0.07%
Clostridium saccharolyticum	24	0.06%
Vallitalea pronyensis	23	0.06%
unclassified Lachnospiraceae	23	0.06%
unclassified Tannerella	23	0.06%
Prevotella denticola	22	0.06%
Acetivibrio ethanolgignens	22	0.06%
Ruminococcus bromii	22	0.06%
Clostridium quinii	22	0.06%
Dialister succinatiphilus	21	0.05%
Blautia glucerasea	21	0.05%
Candidatus Soleaferrea	21	0.05%
Eubacterium siraeum	21	0.05%
Clostridium populeti	21	0.05%
Clostridium polysaccharolyticum	21	0.05%
Prevotella maculosa	20	0.05%

Christensenella minuta	20	0.05%
Clostridium aldenense	20	0.05%
Prevotella loescheii	19	0.05%
Brassicibacter thermophilus	19	0.05%
Intestinimonas timonensis	18	0.05%
Oribacterium sinus	18	0.05%
unclassified Clostridiales	17	0.04%
unclassified Turicibacter	17	0.04%
Campylobacter lanienae	16	0.04%
Clostridium cellulolyticum	16	0.04%
Mitsuokella jalaludinii	16	0.04%
Erysipelothrix inopinata	16	0.04%
Collinsella aerofaciens	16	0.04%
Coprococcus eutactus	15	0.04%
Papillibacter cinnamivorans	15	0.04%
Blautia faecis	15	0.04%
Ruthenibacterium lactatiformans	15	0.04%
Sutterella stercoricanis	15	0.04%
Prevotella dentasini	14	0.04%
Asaccharospora irregularis	14	0.04%
Desulfovibrio fairfieldensis	14	0.04%
Herbinix luporum	14	0.04%
Ruminococcus callidus	13	0.03%
Hallella seregens	13	0.03%
Acetanaerobacterium elongatum	13	0.03%
Mogibacterium diversum	13	0.03%
Peptococcus simiae	13	0.03%
unclassified Ruminococcaceae	13	0.03%
Eisenbergiella tayi	12	0.03%
unclassified Rikenella	12	0.03%
Clostridium phoceensis	12	0.03%
Falcatimonas natans	12	0.03%
Bacteroides intestinalis	11	0.03%
Roseburia hominis	11	0.03%
Lactobacillus hamsteri	11	0.03%
Clostridium bovipellis	11	0.03%
Pseudomonas fluorescens	11	0.03%
Saccharofermentans acetigenes	11	0.03%
Prevotella shahii	10	0.03%
Clostridium lavalense	10	0.03%
unclassified Porphyromonadaceae	10	0.03%
Turicibacter sanguinis	10	0.03%
Anaerostipes hadrus	10	0.03%
Candidatus Dorea	10	0.03%
Clostridium aminobutyricum	10	0.03%
Eubacterium oxidoreducens	10	0.03%
Intestinimonas massiliensis	10	0.03%
Bacteroides heparinolyticus	10	0.03%
Megasphaera hominis	9	0.02%
Eubacterium rangiferina	9	0.02%

Anaerobium acetethylicum	9	0.02%
Anaerocolumna cellulosilytica	9	0.02%
Gracilibacter thermotolerans	9	0.02%
Mitsuokella multacida	9	0.02%
Anaerovorax odorimutans	8	0.02%
Ruminococcus albus	8	0.02%
Olsenella scatoligenes	8	0.02%
Clostridium xylanolyticum	8	0.02%
Clostridium methylpentosum	8	0.02%
Parabacteroides chinchillae	8	0.02%
Oscillibacter valericigenes	8	0.02%
Enorma massiliensis	8	0.02%
Anaeromassilibacillus senegalensis	8	0.02%
unclassified Clostridium	8	0.02%
Lactobacillus rogosae	8	0.02%
Blautia luti	8	0.02%
Clostridium fusiformis	8	0.02%
Methylocystis rosea	8	0.02%
unclassified Acetivibrio	8	0.02%
Clostridium symbiosum	8	0.02%
Pseudoflavonifractor capillosus	8	0.02%
Helicobacter rodentium	8	0.02%
Barnesiella viscericola	7	0.02%
Hespellia porcina	7	0.02%
Agathobacter ruminis	7	0.02%
Clostridium chartatabidum	7	0.02%
Lactobacillus reuteri	7	0.02%
Holdemania filiformis	7	0.02%
Lactobacillus secaliphilus	7	0.02%
Abyssivirga alkaniphila	7	0.02%
Lactobacillus gallinarum	7	0.02%
unclassified Bacillus	7	0.02%
Prevotella bivia	7	0.02%
Enterorhabdus mucosicola	6	0.02%
Prevotella paludivivens	6	0.02%
Selenomonas ruminantium	6	0.02%
Clostridium oroticum	6	0.02%
Bifidobacteriaceae genomosp.	6	0.02%
Catonella morbi	6	0.02%
Gorbachella massiliensis	6	0.02%
unclassified Bacteroides	6	0.02%
Clostridium cellobioparum	6	0.02%
Paludibacter propionicigenes	6	0.02%
Clostridium clostridioforme	6	0.02%
Subdoligranulum variabile	6	0.02%
Bacteroides galacturonicus	6	0.02%
Prevotella baroniae	6	0.02%
Clostridium fimetarium	6	0.02%
Treponema berlinense	6	0.02%
Clostridium asparagiforme	6	0.02%

Mageeibacillus indolicus	6	0.02%
Ruminococcus gnavus	5	0.01%
Mucispirillum schaedleri	5	0.01%
Lactobacillus amylolyticus	5	0.01%
Peptococcus niger	5	0.01%
Elbe River	5	0.01%
unclassified Lactobacillus	5	0.01%
methanogenic archaeon	5	0.01%
Denitrobacterium detoxificans	5	0.01%
Clostridium leptum	5	0.01%
unclassified Erysipelotrichaceae	5	0.01%
Prevotella buccalis	5	0.01%
unclassified Alloprevotella	5	0.01%
Prevotella buccae	5	0.01%
Anaerostipes butyraticus	5	0.01%
Lutispora thermophila	4	0.01%
Pediococcus acidilactici	4	0.01%
Prevotella marshii	4	0.01%
Caloranaerobacter azorensis	4	0.01%
Parabacteroides goldsteinii	4	0.01%
Ethanoligenens harbinense	4	0.01%
Clostridium chauvoei	4	0.01%
Bacteroides pectinophilus	4	0.01%
unclassified Paludibacter	4	0.01%
Paraclostridium benzoelyticum	4	0.01%
Coprobacillus cateniformis	4	0.01%
Desulfotomaculum guttoideum	4	0.01%
Clostridioides difficile	4	0.01%
unclassified Clostridia	4	0.01%
unclassified Sporobacter	4	0.01%
Prevotella albensis	4	0.01%
Marvinbryantia formatexigens	4	0.01%
Eubacterium infirmum	4	0.01%
Olsenella umbonata	4	0.01%
Clostridium hungatei	4	0.01%
Blautia schinkii	4	0.01%
Bacteroides clarus	4	0.01%
Anaerocolumna aminovalerica	4	0.01%
unclassified Cryptanaerobacter	4	0.01%
Parasutterella secunda	4	0.01%
Porphyromonas catoniae	4	0.01%
Anaerocolumna xylanovorans	4	0.01%
Asteroleplasma anaerobium	4	0.01%
Clostridium disporicum	4	0.01%
Anaerobiospirillum succiniciproducens	4	0.01%
Robinsoniella peoriensis	4	0.01%
Roseburia intestinalis	4	0.01%
Mobilitalea sibirica	4	0.01%
unclassified Wautersiella	3	0.01%
Prevotella bryantii	3	0.01%

Parasutterella excrementihominis	3	0.01%
Acidaminococcus fermentans	3	0.01%
Sphaerochaeta coccoides	3	0.01%
Blautia producta	3	0.01%
Eubacterium contortum	3	0.01%
Bacteroides dorei	3	0.01%
Acidaminobacter hydrogenoformans	3	0.01%
Clostridium aerotolerans	3	0.01%
Anaerostipes rhamnosivorans	3	0.01%
Prevotella scopos	3	0.01%
Bacteroides coprophilus	3	0.01%
Eubacterium sulci	3	0.01%
unclassified Bacteroidaceae	3	0.01%
Clostridium hiranonis	3	0.01%
unclassified Subdoligranulum	3	0.01%
Bacteroides caecicola	3	0.01%
Corynebacterium provencense	3	0.01%
unclassified Eubacterium	3	0.01%
Pseudobutyrivibrio ruminis	3	0.01%
Clostridium butyricum	3	0.01%
Bacteroides helcogenes	3	0.01%
Treponema bryantii	3	0.01%
unclassified Planctomycetales	3	0.01%
Butyrivibrio crossotus	3	0.01%
Bacteroidales genomosp.	3	0.01%
Candidatus Treponema	3	0.01%
Natranaerovirga pectinivora	3	0.01%
Propionispira arcuata	3	0.01%
Flavonifractor plautii	3	0.01%
Clostridium taeniosporum	2	0.01%
Parabacteroides merdae	2	0.01%
Eubacterium ventriosum	2	0.01%
Lachnospiraceae oral	2	0.01%
Clostridium tertium	2	0.01%
unclassified Deltaproteobacteria	2	0.01%
Lachnospira multipara	2	0.01%
metal-contaminated soil	2	0.01%
Ercella succinigenes	2	0.01%
unclassified Anaerovibrio	2	0.01%
Bacteroides caecigallinarum	2	0.01%
Bacteroides oleiciplenus	2	0.01%
actinobacterium SCGC	2	0.01%
unclassified Succinivibrio	2	0.01%
Natronincola histidinovorans	2	0.01%
Clostridium celerecrescens	2	0.01%
Slackia isoflavoniconvertens	2	0.01%
unclassified Methanobrevibacter	2	0.01%
Clostridium oceanicum	2	0.01%
Bacteroides massiliensis	2	0.01%
Allisonella histaminiformans	2	0.01%

Acetivibrio cellulolyticus	2	0.01%
unclassified Megasphaera	2	0.01%
Clostridium hydrogeniproducens	2	0.01%
unclassified Enterococcus	2	0.01%
Ferruginibacter profundus	2	0.01%
Faecalicoccus acidiformans	2	0.01%
Clostridium viride	2	0.01%
Clostridium hveragerdense	2	0.01%
unclassified Lactobacillaceae	2	0.01%
Treponema parvum	2	0.01%
Alloprevotella tannerae	2	0.01%
Clostridium botulinum	2	0.01%
Clostridium bolteae	2	0.01%
unclassified Oscillibacter	2	0.01%
Prevotella timonensis	2	0.01%
unclassified Clostridiaceae	2	0.01%
Oceanirhabdus sediminicola	2	0.01%
Clostridium neopropionicum	2	0.01%
Clostridium straminisolvens	2	0.01%
Defluviitalea saccharophila	2	0.01%
Porphyromonas cangingivalis	2	0.01%
unclassified Ruminococcus	2	0.01%
Caminicella sporogenes	2	0.01%
Prevotella jejuni	2	0.01%
Catabacter hongkongensis	2	0.01%
Escherichia coli	2	0.01%
Methanosphaera cuniculi	2	0.01%
Bacteroides acidifaciens	2	0.01%
Clostridium sartagoforme	2	0.01%
Selenomonas bovis	2	0.01%
Olivibacter sitiensis	2	0.01%
Bacteroides uniformis	2	0.01%
Propionispira paucivorans	2	0.01%
Helicobacter equorum	2	0.01%
Gemmatimonas aurantiaca	2	0.01%
Bacteroides faecis	2	0.01%
Clostridium papyrosolvens	2	0.01%
Anaerosporobacter mobilis	2	0.01%
Caloramator fervidus	2	0.01%
Bacteroides stercoris	2	0.01%
Lachnoanaerobaculum saburreum	2	0.01%
Acetoanaerobium pronyense	1	0%
Desulfotomaculum halophilum	1	0%
Lacibacter cauensis	1	0%
Sutterella wadsworthensis	1	0%
Lactobacillus coleohominis	1	0%
Anaerofustis stercorihominis	1	0%
Bacteroides salanitronis	1	0%
Mycobacterium scrofulaceum	1	0%
Clostridium stercorarium	1	0%

Kiloniella spongiae	1	0%
Lactobacillus oris	1	0%
Mahella australiensis	1	0%
Bacteroides finegoldii	1	0%
Streptomyces chrestomyceticus	1	0%
Stenotrophobacter terrae	1	0%
Pyramidobacter piscolens	1	0%
Clostridium longisporum	1	0%
Hyphomicrobium facile	1	0%
Prevotella saccharolytica	1	0%
Lactobacillus rodentium	1	0%
Methylocystis bryophila	1	0%
Lactobacillus tucceti	1	0%
unclassified Anaerovorax	1	0%
Herbinix hemicellulosilytica	1	0%
Hungatella effluvii	1	0%
Lachnoanaerobaculum umeaense	1	0%
Anaerotruncus colihominis	1	0%
Acidaminococcus intestini	1	0%
Prevotella aff.	1	0%
Selenomonas lacticifex	1	0%
Candidatus Heliomonas	1	0%
Clostridium clariflavum	1	0%
Prevotella fusca	1	0%
Terrabacter terrae	1	0%
Ruminococcus champanellensis	1	0%
Collinsella stercoris	1	0%
Salinibacillus xinjiangensis	1	0%
Bariatricus massiliensis	1	0%
Treponema succinifaciens	1	0%
Dehalogenimonas alkenigignens	1	0%
Slackia exigua	1	0%
Treponema porcinum	1	0%
Bellilinea caldifistulae	1	0%
Anaerofilum pentosovorans	1	0%
unclassified Oscillospira	1	0%
Desulfotomaculum nigrificans	1	0%
Tyzzerella nexilis	1	0%
Propionispira raffinosivorans	1	0%
Bacteroides pyogenes	1	0%
Clostridium sphenoides	1	0%
Prevotella micans	1	0%
Prevotella nanceiensis	1	0%
Howardella ureilytica	1	0%
Rhodospirillum rubrum	1	0%
Porphyromonas pogonae	1	0%
Lactonifactor longoviformis	1	0%
Acetobacter indonesiensis	1	0%
Nocardioidaceae str.	1	0%
Bacteroides zoogleoformans	1	0%

Clostridium lactatifermentans	1	0%
Olsenella uli	1	0%
Casaltella massiliensis	1	0%
Clostridium aurantibutyricum	1	0%
Bacillus longiquaesitum	1	0%
unclassified Peptococcus	1	0%
Collinsella intestinalis	1	0%
Campylobacter hyointestinalis	1	0%
Pseudonocardia petroleophila	1	0%
Prevotella enoeca	1	0%
unclassified Dialister	1	0%
Clostridium tarantellae	1	0%
Olsenella profusa	1	0%
Drancourtella massiliensis	1	0%
unclassified Veillonellaceae	1	0%
Nakamurella flavida	1	0%
Parvibacter caecicola	1	0%
Pleomorphochaeta multiformis	1	0%
unclassified Erysipelotrichia	1	0%
unclassified Petrimonas	1	0%
unclassified Eubacteriaceae	1	0%
unclassified Paenibacillaceae	1	0%
unclassified Treponema	1	0%
Lachnoanaerobaculum cf.	1	0%
Lachnoclostridium phytofermentans	1	0%
Eubacterium xylanophilum	1	0%
Geosporobacter ferrireducens	1	0%
unclassified Lachnospira	1	0%
Eubacterium pyruvativorans	1	0%
Lactobacillus intestinalis	1	0%
Clostridium tepidiprofundi	1	0%
Hathewaya limosa	1	0%
Variovorax boronicumulans	1	0%
Devosia ginsengisoli	1	0%
Yersinia kristensenii	1	0%
Candidatus Azospirillum	1	0%
Pediococcus ethanolidurans	1	0%
Caloramator quimbayensis	1	0%
Ruminococcus lactaris	1	0%
Clostridium indolis	1	0%
Dialister invisus	1	0%
Hyphomicrobium aestuarii	1	0%
Chloroidium saccharophilum	1	0%
Sphaerochaeta pleomorpha	1	0%
Elusimicrobium minutum	1	0%
Carboxylicivirga mesophila	1	0%
Bauldia litoralis	1	0%
Methyloligella halotolerans	1	0%
Methylomonas lenta	1	0%
Anaeromicrobium sediminis	1	0%

alpha proteobacterium	1	0%
unclassified Catonella	1	0%
Clostridium sporosphaeroides	1	0%
Brevundimonas staleyi	1	0%
Bacteroides timonensis	1	0%
Desulfotomaculum alcoholivorax	1	0%
unclassified Roseburia	1	0%
unclassified Proteus	1	0%
Chelativorans composti	1	0%
Methylosinus trichosporium	1	0%
Rhodobium orientis	1	0%
Bacteroides graminisolvens	1	0%
Aeriscardovia aeriphila	1	0%
Luteimicrobium album	1	0%
Granulicella paludicola	1	0%
Clostridium intestinale	1	0%
Treponema brennaborense	1	0%
Ferruginibacter yonginensis	1	0%
Oligosphaera ethanolica	1	0%
Candidatus Filomicrobium	1	0%
Anaeroplasma bactoclasticum	1	0%
Bacteroides cellulosilyticus	1	0%
