# Supplementary material for: Influence of the Fermented Feed and Vaccination and Their Interaction on Parameters of Large White/Norwegian Landrace Piglets
Source: Animals (Basel). 2020 Jul 15;10(7):1201. doi: 10.3390/ani10071201 (PMC7401620; doi:10.3390/ani10071201)
Supplement: Supplementary file 1 [file animals-10-01201-s001.zip › Table S9 Differences between microbiological parameters of the piglets feces.pdf]

Supplementary file 9. Differences between microbiological parameters of the piglets' feces.

	$1S_{nonV}$ /$2S_{nonV}$	$1S_V$ / $2S_V$	$1RF_{nonV}$ / $2RF_{nonV}$	$1RF_V$ / $2RF_V$	$1S_{nonV}$ / $1S_V$	$1S_{nonV}$ /$1RF_{nonV}$	$1S_{nonV}$ / $1RF_V$	$1S_V$ / $1RF_{nonV}$	$1S_V$ /$1RF_V$	$1RF_{nonV}$ / $1RF_V$	$2S_{nonV}$ / $2S_V$	$2S_{nonV}$ / $2RF_{nonV}$	$2S_{nonV}$ / $2RF_V$	$2S_V$ / $2RF_{nonV}$	$2S_V$ / $2RF_V$	$2RF_{nonV}$ / $2RF_V$
Significance of the differences between groups (P)																
LAB	0.001	0.002	0.0001	0.001	0.001	0.001	0.001	0.011	0.008	0.074	0.001	0.001	0.368	0.001	0.001	0.001
TBC	0.001	0.478	0.001	0.001	0.001	0.001	0.002	0.008	0.001	0.0001	0.001	0.225	0.001	0.0001	0.001	0.001
TEC	0.001	0.001	0.001	0.001	0.0001	0.004	0.001	0.001	0.003	0.016	0.009	0.003	0.001	0.001	0.001	0.008
Y/M	0.004	0.001	0.001	0.0001	0.001	0.0001	0.003	0.001	0.001	0.0001	0.001	0.001	0.049	0.001	0.001	0.001

LAB—lactic acid bateria; TBC— total bacteria count; TEC—total enterobacteria count; Y/M—yeast/mold count;
S_{nonV} - non vaccinated piglets group fed with soya meal, S_V - vaccinated piglets fed with soya meal, RF_{nonV} - non vaccinated piglets group fed with fermented rapeseed meal, RF_V - vaccinated piglets group fed with fermented rapeseed meal.
1 – at the beginning of experiment, 2 – at the of experiment.
Data are presented as average (n = 10/group).
