# Supplementary material for: Influence of the Fermented Feed and Vaccination and Their Interaction on Parameters of Large White/Norwegian Landrace Piglets
Source: Animals (Basel). 2020 Jul 15;10(7):1201. doi: 10.3390/ani10071201 (PMC7401620; doi:10.3390/ani10071201)
Supplement: Supplementary file 1 [file animals-10-01201-s001.zip › Table S10 Differences between microbiological parameters of the piglets.pdf]

Supplementary file 10. Differences between blood parameters of the piglets'.

Blood parameters	$1S_{nonV}/2S_{nonV}$	$1S_V/2S_V$	$1RF_{nonV}/2RF_{nonV}$	$1RF_V/2RF_V$	$1S_{nonV}/1S_V$	$1S_{nonV}/1RF_{nonV}$	$1S_{nonV}/1RF_V$	$1S_V/1RF_{nonV}$	$1S_V/1RF_V$	$1RF_{nonV}/1RF_V$	$2S_{nonV}/2S_V$	$2S_{nonV}/2RF_{nonV}$	$2S_{nonV}/2RF_V$	$2S_V/2RF_{nonV}$	$2S_V/2RF_V$	$2RF_{nonV}/2RF_V$
	Significance of the differences between groups (P)															
TTH	0.83	0.171	0.731	0.001	0.361	0.616	0.306	0.072	0.318	0.556	0.48	0.141	0.002	0.957	0.001	0.002
ALB	**0.032**	**0.004**	**0.001**	**0.01**	0.326	**0.047**	**0.018**	**0.003**	**0.011**	0.433	0.197	0.5	0.061	0.075	**0.003**	**0.002**
TP	**0.002**	**0.002**	**0.015**	**0.001**	0.194	**0.004**	0.169	0.495	0.212	0.065	**0.001**	0.278	**0.001**	**0.04**	**0.001**	**0.044**
UREA	0.239	0.167	**0.025**	**0.005**	0.136	0.066	0.48	0.169	0.394	0.889	0.155	0.086	**0.001**	0.06	0.087	0.07
CREA	0.283	0.806	**0.019**	0.431	0.419	**0.048**	0.953	**0.024**	0.381	0.222	0.342	0.134	0.085	0.057	**0.005**	0.445
ALT	**0.01**	**0.001**	**0.001**	0.067	0.067	0.223	**0.027**	**0.023**	**0.036**	0.255	**0.039**	**0.002**	**0.014**	**0.01**	**0.028**	**0.001**
AST	0.095	0.792	0.086	0.203	0.273	**0.039**	0.125	0.182	**0.037**	0.33	0.06	**0.004**	**0.007**	0.12	0.322	**0.002**
ALP	0.125	0.172	**0.017**	0.077	0.245	0.061	0.505	0.133	0.065	0.16	0.331	0.569	0.364	**0.017**	0.306	0.873
TBI	0.199	0.22	0.25	0.45	0.396	0.396	0.396	0.34	0.32	0.21	0.184	0.184	0.223	0.35	0.242	0.022
CHOL	**0.001**	**0.011**	0.051	**0.047**	0.982	0.334	**0.003**	0.173	0.056	0.535	**0.004**	0.084	**0.019**	0.374	0.711	0.271
HDL-C	**0.001**	**0.001**	**0.01**	0.114	**0.011**	**0.041**	**0.003**	**0.049**	**0.01**	0.373	**0.006**	**0.018**	**0.005**	**0.04**	**0.006**	**0.021**
LDL-C	**0.009**	**0.007**	0.137	0.272	0.763	0.728	**0.001**	0.205	0.121	0.376	0.089	0.211	0.15	**0.022**	**0.003**	**0.019**
TG	**0.041**	0.073	**0.012**	**0.001**	0.764	0.891	**0.002**	0.263	**0.07**	0.079	1	**0.005**	**0.006**	**0.001**	**0.001**	**0.014**
GLU	0.536	0.785	0.128	**0.001**	0.329	0.417	**0.044**	0.97	0.211	0.192	**0.01**	0.113	**0.03**	0.213	0.78	0.671
IP	**0.034**	0.062	**0.025**	**0.001**	0.135	**0.006**	**0.011**	**0.001**	0.168	0.073	**0.001**	**0.01**	0.322	**0.046**	1	0.244
Fe	0.169	**0.015**	0.069	**0.04**	0.92	**0.021**	0.921	**0.001**	**0.005**	**0.001**	0.443	0.07	0.073	**0.038**	**0.045**	0.084
Mg	0.274	0.424	0.316	0.121	**0.035**	0.079	0.051	**0.024**	0.302	**0.043**	**0.037**	**0.001**	**0.001**	0.428	0.337	**0.004**
Ca	**0.033**	**0.001**	0.165	**0.007**	0.704	0.399	0.413	0.061	0.492	0.111	**0.025**	**0.045**	**0.023**	0.467	**0.003**	0.202
K	**0.003**	**0.014**	0.354	**0.002**	**0.014**	0.053	**0.005**	**0.024**	0.846	**0.001**	**0.035**	0.066	**0.001**	0.114	**0.009**	0.371
Na	0.097	**0.001**	**0.022**	0.061	0.105	0.663	0.315	**0.012**	**0.04**	**0.004**	1	0.34	0.08	**0.001**	**0.018**	**0.026**
T3	**0.011**	**0.009**	0.099	0.43	**0.011**	**0.013**	0.971	**0.012**	0.793	0.762	**0.003**	**0.001**	**0.009**	**0.001**	**0.011**	0.214
T4	0.094	0.084	**0.006**	0.052	0.429	**0.002**	0.247	0.071	0.078	0.072	**0.017**	**0.001**	**0.001**	**0.014**	**0.017**	0.277
IgG	**0.024**	0.114	**0.037**	0.329	0.294	**0.031**	0.234	**0.03**	0.753	0.126	0.083	0.199	0.474	0.286	0.43	0.41
vitB12	0.061	**0.047**	0.211	**0.005**	**0.015**	**0.014**	0.168	**0.011**	**0.001**	**0.002**	**0.037**	**0.021**	**0.033**	**0.001**	**0.018**	0.429

S_{nonV} - non vaccinated piglets group fed with soya meal, S_V - vaccinated piglets fed with soya meal, RF_{nonV} - non vaccinated piglets group fed with fermented rapeseed meal, RF_V - vaccinated piglets group fed with fermented rapeseed meal. 1 – at the beginning of experiment, 2 – at the of experiment. Data are presented as average (n = 10/group).
