# Supplementary material for: Influence of the Fermented Feed and Vaccination and Their Interaction on Parameters of Large White/Norwegian Landrace Piglets
Source: Animals (Basel). 2020 Jul 15;10(7):1201. doi: 10.3390/ani10071201 (PMC7401620; doi:10.3390/ani10071201)
Supplement: Supplementary file 1 [file animals-10-01201-s001.zip › Table S1 Species SnonV group before experiment.pdf]

Species SnonV group before experiment	Number of reads
Prevotella copri	11871
Lactobacillus amylovorus	9566
Unclassified	872
Barnesiella intestinihominis	673
Lactobacillus reuteri	649
Faecalibacterium prausnitzii	576
Prevotella stercorea	547
Prevotella oris	497
Clostridium cellulovorans	480
Eubacterium rectale	413
Prevotella brevis	369
Roseburia faecis	365
Terrisporobacter glycolicus	355
Blautia wexlerae	298
Anaerovibrio lipolyticus	292
Prevotella oralis	277
Alloprevotella rava	274
Gemmiger formicilis	248
Coprococcus catus	239
Bacteroidales oral	179
Fusicatenibacter saccharivorans	175
Flintibacter butyricus	171
Eubacterium coprostanoligenes	155
Butyricicoccus pullicaecorum	136
Lactobacillus crispatus	135
Paraprevotella clara	132
Lactobacillus delbrueckii	127
unclassified Bacteroidales	126
Catenibacterium mitsuokai	115
Phascolarctobacterium succinatutens	113
Clostridium celatum	111
Escherichia coli	109
Intestinimonas butyriciproducens	108
Oscillospira guilliermondii	106
Lactobacillus jensenii	105
Intestinibacter bartlettii	104
Prevotella salivae	97
Oscillibacter ruminantium	93
Prevotella paludivivens	92
Ruminococcus bicirculans	89
Ruminococcus flavefaciens	87
Lactobacillus kitasatonis	87
Murimonas intestini	86
Holdemanella biformis	82
Coprococcus comes	81
Ruminococcus faecis	81
unclassified Prevotella	77
Ruminococcus torques	77
Eubacterium eligens	72

Sporobacter termitidis	72
Blautia obeum	72
Lactobacillus pontis	71
Ruminiclostridium thermocellum	69
Eubacterium ramulus	69
Prevotella dentalis	68
Dorea longicatena	67
Prevotella conceptionensis	65
Parabacteroides goldsteinii	63
Eubacterium hallii	60
Parabacteroides distasonis	59
Romboutsia sedimentorum	57
Lactobacillus panis	55
Lactobacillus helveticus	55
Eubacterium ruminantium	50
unclassified Tannerella	48
Fournierella massiliensis	47
Candidatus Soleaferrea	47
Campylobacter lanienae	47
Collinsella aerofaciens	47
Clostridium xylanolyticum	46
unclassified Turicibacter	45
Clostridium aldenense	44
Blautia massiliensis	43
Anaerotaenia torta	42
Saccharofermentans acetigenes	42
Lactobacillus acidophilus	42
Blautia stercoris	41
Clostridium polysaccharolyticum	40
Lactobacillus frumenti	40
Agathobacter ruminis	39
Blautia glucerasea	39
Turicibacter sanguinis	38
Anaerobacterium chartisolvens	38
unclassified Barnesiella	38
Blautia producta	38
Clostridium saccharolyticum	38
Clostridium quinii	37
unclassified Rikenella	37
Butyrivibrio fibrisolvens	37
Clostridium asparagiforme	36
Candidatus Dorea	36
unclassified Deltaproteobacteria	35
Megasphaera elsdenii	34
Clostridium phoceensis	34
Hungatella hathewayi	34
Roseburia inulinivorans	33
Intestinimonas timonensis	32
Prevotella genomosp.	31
Ruminococcus bromii	31

Mogibacterium diversum	30
Roseburia hominis	29
Anaerobium acetethylicum	29
Erysipelothrix inopinata	29
unclassified Lachnospiraceae	29
Acetivibrio ethanolgignens	28
Falcatimonas natans	27
Dorea formicigenerans	26
Methylocystis rosea	26
Eubacterium desmolans	26
Prevotella loescheii	26
Prevotella ruminicola	26
Lachnospira pectinoschiza	25
Eubacteriaceae oral	25
Clostridium aminobutyricum	25
Prevotella buccae	25
Candidatus Treponema	25
Treponema bryantii	24
cyanobacterium enrichment	24
Intestinimonas massiliensis	24
Brassicibacter thermophilus	23
Vallitalea pronyensis	23
Lactobacillus johnsonii	22
Clostridium bovipellis	22
Lactobacillus mucosae	22
Herbinix luporum	22
Clostridium lavalense	21
Oribacterium sinus	20
Bacteroidales genomosp.	20
Asaccharospora irregularis	20
Clostridium leptum	19
Bacteroides pectinophilus	19
Blautia faecis	19
Desulfovibrio fairfieldensis	19
unclassified Clostridium	19
Sphaerochaeta coccoides	19
Eubacterium siraeum	19
Barnesiella viscericola	18
Denitrobacterium detoxificans	18
Gracilibacter thermotolerans	17
Anaerovorax odorimutans	17
unclassified Anaerovibrio	17
Ruminococcus lactaris	17
unclassified Ruminococcaceae	17
Clostridium populeti	17
Blautia schinkii	17
unclassified Prevotellaceae	17
Eisenbergiella tayi	16
Desulfovibrio piger	16
Clostridium fusiformis	15

Clostridium symbiosum	15
Anaerocolumna cellulosilytica	15
Pseudoflavonifractor capillosus	15
Prevotella bivia	15
Blautia luti	14
Alloprevotella tannerae	14
Anaerostipes hadrus	14
Selenomonas bovis	14
Clostridium amylolyticum	14
unclassified Erysipelotrichaceae	14
Lactobacillus agilis	14
Ruthenibacterium lactatiformans	14
Desulfotomaculum guttoideum	13
Parabacteroides chinchillae	13
Oscillibacter valericigenes	13
Papillibacter cinnamivorans	13
Hespellia porcina	13
Anaeromassilibacillus senegalensis	12
unclassified Porphyromonadaceae	12
Olsenella scatoligenes	12
Coprococcus eutactus	12
Mucispirillum schaedleri	12
Eubacterium oxidoreducens	12
Natranaerovirga pectinivora	12
Solobacterium moorei	12
Holdemania filiformis	12
Eubacterium contortum	11
Bacteroides barnesiae	11
unclassified Mollicutes	11
Treponema parvum	11
Peptococcus simiae	11
Selenomonas ruminantium	11
Ruminococcus callidus	11
Ruminococcus gnavus	11
Prevotella maculosa	11
methanogenic archaeon	11
unclassified Bacteroides	11
Caloramator fervidus	11
Porphyromonas catoniae	10
Clostridium disporicum	10
Christensenella minuta	10
Prevotella shahii	10
unclassified Clostridiales	10
Clostridium clostridioforme	10
Treponema porcinum	10
Bacteroides uniformis	10
Prevotella denticola	10
unclassified Clostridia	9
Acetanaerobacterium elongatum	9
Lachnospira multipara	9

Clostridium cellobioparum	9
Ruminococcus albus	9
Enorma massiliensis	9
Clostridium methylpentosum	9
Intestinimonas gabonensis	9
unclassified Enterococcus	9
Ruminococcus champanellensis	9
Eubacterium infirmum	9
unclassified Planctomycetales	9
Propionispira arcuata	8
Lactobacillus vaginalis	8
Clostridium chartatabidum	8
Pseudomonas fluorescens	8
Prevotella bryantii	8
Bacteroides stercoris	8
Clostridium oroticum	8
Prevotella dentasini	7
Acidaminobacter hydrogenoformans	7
Abyssivirga alkaniphila	7
Clostridium lactatifermentans	7
Sutterella stercoricanis	7
Acetivibrio cellulolyticus	7
Clostridium hveragerdense	7
Anaerostipes butyraticus	7
Caminicella sporogenes	7
Campylobacter coli	7
Lactobacillus amylolyticus	7
Mobilitalea sibirica	7
Fibrobacter intestinalis	7
Oligosphaera ethanolica	7
Robinsoniella peoriensis	6
Kluyvera georgiana	6
Lactobacillus secaliphilus	6
Eubacterium rangiferina	6
Clostridium tertium	6
Hallella seregens	6
Macellibacteroides fermentans	6
unclassified Lactobacillus	6
Bacteroides intestinalis	6
unclassified Methanobrevibacter	6
Catabacter hongkongensis	6
Roseburia intestinalis	6
Prevotella baroniae	6
Flavonifractor plautii	6
Bacteroides caecicola	6
Bacteroides galacturonicus	5
Corynebacterium provencense	5
Olivibacter sitiensis	5
Cellulosilyticum ruminicola	5
Sphaerochaeta pleomorpha	5

Treponema berlinense	5
Clostridium glycyrrhizinilyticum	5
Clostridium sulfidigenes	5
Faecalitalea cylindroides	5
Parvibacter caecicola	5
Clostridium sphenoides	5
Paludibacter propionicigenes	5
unclassified Candidatus Glomeribacter	5
Lachnoanaerobaculum umeaense	5
Marvinbryantia formatexigens	5
unclassified Acetivibrio	5
alpha proteobacterium	5
unclassified Paludibacter	5
unclassified Alloprevotella	5
Desulfovibrio desulfuricans	5
Bacteroides caccae	4
Eisenbergiella massiliensis	4
unclassified Eubacterium	4
Alkalibacter saccharofermentans	4
Clostridium cellulolyticum	4
Garciella nitratireducens	4
Clostridium celerecrescens	4
Butyrivibrio crossotus	4
Succinivibrio dextrinosolvens	4
Bacteroides faecis	4
Sutterella parvirubra	4
Succiniclasticum ruminis	4
Lactobacillus rogosae	4
Methylocystis echinoides	4
Mitsuokella jalaludinii	4
Prevotella buccalis	4
Prevotella scopos	4
Parasporobacterium paucivorans	4
Clostridioides difficile	4
Anaerostipes rhamnosivorans	4
Acidaminococcus intestini	4
Elbe River	4
Faecalicoccus acidiformans	4
Geosporobacter ferrireducens	4
Clostridium isatidis	4
Clostridium sartagoforme	4
Enterorhabdus mucosicola	4
Blautia coccoides	4
Bacteroides heparinolyticus	4
Lactobacillus fermentum	4
Porphyromonas cangingivalis	4
Parasutterella secunda	4
Prevotella fusca	4
Olsenella uli	4
Prevotella saccharolytica	4

Hungatella effluvii	4
Bacteroides paurosaccharolyticus	3
Clostridium hiranonis	3
Bacteroides salyersiae	3
unclassified Erysipelotrichia	3
Desulfotomaculum nigrificans	3
Lactobacillus hamsteri	3
Selenomonas sputigena	3
Anaeroplasma bactoclasticum	3
Faecalicoccus pleomorphus	3
Clostridium butyricum	3
Lactonifactor longoviformis	3
Lutispora thermophila	3
Subdoligranulum variabile	3
Breznakia pachnodae	3
Clostridium aminophilum	3
unclassified Roseburia	3
Escherichia albertii	3
Bacteroides oleiciplenus	3
Pithomyces chartarum	3
Marmoricola bigeumensis	3
Clostridium tepidiprofundi	3
Eubacterium minutum	3
Gorbachella massiliensis	3
Treponema brennaborense	3
Clostridium hylemonae	3
Prevotella enoeca	3
Clostridium tarantellae	3
Desulfotomaculum tongense	3
Thermotalea metallivorans	3
Prevotella melaninogenica	3
Bacteroides clarus	3
Clostridium aerotolerans	3
unclassified Treponema	3
Prevotella albensis	3
Prevotella micans	3
Propionispira paucivorans	3
Clostridium clariflavum	2
Collinsella intestinalis	2
unclassified Olsenella	2
type II	2
Bacteroides graminisolvens	2
unclassified Alphaproteobacteria	2
Pediococcus acidilactici	2
Peptoclostridium acidaminophilum	2
Tyzzerella nexilis	2
Bacteroides helcogenes	2
Proteiniborus ethanoligenes	2
unclassified Bacteroidia	2
Alkaliphilus crotonatoxidans	2

metal-contaminated soil	2
Clostridium intestinale	2
endosymbiont of	2
Hydrogenoanaerobacterium saccharovorans	2
Blautia hydrogenotrophica	2
Prevotella multiformis	2
Clonostachys cf.	2
Clostridium fimetarium	2
Lactobacillus psittaci	2
Prevotella corporis	2
Clostridium indolis	2
Bacteroides nordii	2
Clostridium chauvoei	2
Eubacterium tenue	2
unclassified Lactobacillaceae	2
Pseudobutyrivibrio xylanivorans	2
Oceanirhabdus sediminicola	2
unclassified Bacteroidaceae	2
Methylocystis bryophila	2
Clostridium scindens	2
Dehalobacterium formicoaceticum	2
unclassified Ruminococcus	2
Cladosporium cladosporioides	2
Clostridium cadaveris	2
Acetobacter indonesiensis	2
Bacteroides acidifaciens	2
Bacteroides coprocola	2
unclassified Veillonellaceae	2
Anaerosporobacter mobilis	2
Helicobacter rodentium	2
Tepidibacter mesophilus	2
unclassified Faecalibacterium	2
Bacteroides ovatus	2
Anaerobiospirillum succiniciproducens	2
Mageeibacillus indolicus	2
Chaetomium globosum	2
Paeniclostridium sordellii	2
Campylobacter jejuni	2
Eubacterium pyruvativorans	2
Pleomorphochaeta multiformis	2
Anaerocolumna xylanovorans	2
Parabacteroides johnsonii	2
Anaerofustis stercorihominis	2
Bacteroides caecigallinarum	2
Peptococcus niger	2
Oxalobacter formigenes	2
Propionispira raffinosivorans	2
Caproiciproducens galactitolivorans	2
Hespellia stercorisuis	2
Syntrophococcus sucromutans	2

Olsenella profusa	2
unclassified Oribacterium	1
Nocardioides kribbensis	1
Tetrasphaera vanveenii	1
Oligoflexus tunisiensis	1
Bacillus niacini	1
Clostridium propionicum	1
Terrimonas lutea	1
Paraeggerthella hongkongensis	1
Pseudohaliea rubra	1
Clostridium colinum	1
uncultivated soil	1
Erysipelothrix rhusiopathiae	1
unclassified Petrimonas	1
Terrisporobacter petrolearius	1
Pedosphaera parvula	1
Eubacterium brachy	1
unclassified Candidatus Solibacter	1
unclassified Christensenella	1
Eubacterium xylanophilum	1
Clostridium akagii	1
unclassified Dysgonomonas	1
Hyphomicrobium aestuarii	1
Methylobacterium goesingense	1
unclassified Collinsella	1
Ethanoligenens harbinense	1
Eubacterium sulci	1
Lactobacillus gallinarum	1
Bittarella massiliensis	1
Pseudomonas stutzeri	1
Pseudobutyrivibrio ruminis	1
unclassified Spirochaetia	1
Gaiella occulta	1
Paludibaculum fermentans	1
Clostridium papyrosolvens	1
Lactobacillus curvatus	1
unclassified Actinobacteria	1
Coprobacter secundus	1
Defluviitalea raffinosedens	1
Alistipes shahii	1
Treponema zioleckii	1
Clostridium neopropionicum	1
Prevotella histicola	1
unclassified Burkholderia	1
Pseudogymnoascus roseus	1
Clostridium tyrobutyricum	1
Acholeplasma parvum	1
Porphyromonas pasteri	1
beta proteobacterium	1
unclassified Oscillospira	1

unclassified Bifidobacterium	1
Clostridium cavendishii	1
unclassified Geobacter	1
unclassified Nitrospira	1
Oxobacter pfennigii	1
Lactobacillus casei	1
Mogibacterium vescum	1
Thermoflavimicrobium dichotomicum	1
Anaerocolumna aminovalerica	1
Clostridium aurantibutyricum	1
Cladosporium herbarum	1
Butyricimonas paravirosa	1
Actinomycetales str.	1
Slackia isoflavoniconvertens	1
Flavobacterium succinicans	1
Aestuariispira insulae	1
Pseudomonas putida	1
Bifidobacterium pseudolongum	1
Methanobrevibacter smithii	1
Acidaminococcus fermentans	1
Eubacterium plexicaudatum	1
Bariatricus massiliensis	1
Eubacterium ventriosum	1
unclassified Acidobacterium	1
Lactobacillus gasseri	1
Finegoldia magna	1
Lachnoanaerobaculum saburreum	1
Pseudomonas psychrophila	1
Bacillus nealsonii	1
Acetatifactor muris	1
Lachnoclostridium phytofermentans	1
Parabacteroides merdae	1
Kribbella swartbergensis	1
Aminobacter aminovorans	1
Bacillus funiculus	1
Pseudogymnoascus pannorum	1
Porphyromonas pogonae	1
Arthrobacter pascens	1
Paeniclostridium ghonii	1
Catonella morbi	1
Ercella succinigenes	1
Ureibacillus defluvii	1
Methylosinus trichosporium	1
Lactobacillus antri	1
unclassified Spirochaeta	1
actinobacterium SCGC	1
unclassified Clostridiales Family XIII. Incertae Sedis	1
Bacteroides zoogleoformans	1
Selenomonas artemidis	1
unclassified Anaerovorax	1

Helicobacter equorum	1
Nitrospira calida	1
Mycobacterium fortuitum	1
Sporosarcina soli	1
Asteroleplasma anaerobium	1
Mycobacterium barrassiae	1
unclassified Wautersiella	1
Helicobacter rappini	1
Prevotella oulorum	1
unclassified Prolixibacter	1
Lactobacillus intestinalis	1
Clostridium fallax	1
Fibrobacter succinogenes	1
Parasutterella excrementihominis	1
Luteolibacter cuticulihirudinis	1
Sphaerobacter thermophilus	1
Ruminococcus gauvreauii	1
Lactobacillus manihotivorans	1
Bacteroides cellulosilyticus	1
Alternaria alternata	1
Defluviitalea phaphyphila	1
Niastella koreensis	1
Lactobacillus rodentium	1
Desulfotomaculum gibsoniae	1
Vitis hybrid	1
Clostridium viride	1
Paludibacter jiangxiensis	1
Clostridium putrefaciens	1
Enterorhabdus caecimuris	1
Adlercreutzia equolifaciens	1
benzene mineralizing	1
Mycobacterium szulgai	1
unclassified Oscillibacter	1
unclassified Solirubrobacter	1
Gordonia caeni	1
Proteocatella sphenisci	1
Treponema succinifaciens	1
unclassified Nitrosovibrio	1
Vallitalea guaymasensis	1
Howardella ureilytica	1
unclassified Cryptanaerobacter	1
Lewinella cohaerens	1
Lactobacillus letivazi	1
candidate division	1
Methanosphaera cuniculi	1
unclassified Pseudomonas	1
Candidatus Heliomonas	1
Streptococcus lutetiensis	1
Prevotella oryzae	1
Bacteroides plebeius	1

Aspergillus tubingensis	1
Variovorax dokdonensis	1
Bacteroides salanitronis	1
Candidatus Stoquefichus	1
Eubacterium saphenum	1
Clostridium piliforme	1
Campylobacter hyointestinalis	1
Clostridium longisporum	1
actinobacterium enrichment	1
delta proteobacterium	1
Bacteroides fragilis	1

Relative abundance
31.95%
25.75%
2.35%
1.81%
1.75%
1.55%
1.47%
1.34%
1.29%
1.11%
0.99%
0.98%
0.96%
0.8%
0.79%
0.75%
0.74%
0.67%
0.64%
0.48%
0.47%
0.46%
0.42%
0.37%
0.36%
0.36%
0.34%
0.34%
0.31%
0.3%
0.3%
0.29%
0.29%
0.29%
0.28%
0.28%
0.26%
0.25%
0.25%
0.24%
0.23%
0.23%
0.23%
0.22%
0.22%
0.22%
0.21%
0.21%
0.19%

0.19%
0.19%
0.19%
0.19%
0.19%
0.18%
0.18%
0.17%
0.17%
0.16%
0.16%
0.15%
0.15%
0.15%
0.13%
0.13%
0.13%
0.13%
0.13%
0.13%
0.12%
0.12%
0.12%
0.12%
0.11%
0.11%
0.11%
0.11%
0.11%
0.11%
0.1%
0.1%
0.1%
0.1%
0.1%
0.1%
0.1%
0.1%
0.1%
0.1%
0.1%
0.1%
0.09%
0.09%
0.09%
0.09%
0.09%
0.09%
0.08%
0.08%

0.08%
0.08%
0.08%
0.08%
0.08%
0.08%
0.07%
0.07%
0.07%
0.07%
0.07%
0.07%
0.07%
0.07%
0.07%
0.07%
0.07%
0.06%
0.06%
0.06%
0.06%
0.06%
0.06%
0.06%
0.06%
0.06%
0.06%
0.05%
0.05%
0.05%
0.05%
0.05%
0.05%
0.05%
0.05%
0.05%
0.05%
0.05%
0.05%
0.05%
0.05%
0.05%
0.05%
0.05%
0.05%
0.05%
0.05%
0.04%
0.04%
0.04%

0.04%
0.04%
0.04%
0.04%
0.04%
0.04%
0.04%
0.04%
0.04%
0.04%
0.04%
0.04%
0.03%
0.03%
0.03%
0.03%
0.03%
0.03%
0.03%
0.03%
0.03%
0.03%
0.03%
0.03%
0.03%
0.03%
0.03%
0.03%
0.03%
0.03%
0.03%
0.03%
0.03%
0.03%
0.03%
0.03%
0.03%
0.03%
0.03%
0.03%
0.03%
0.03%
0.03%
0.03%
0.03%
0.03%
0.03%
0.02%
0.02%
0.02%

0.02%
0.02%
0.02%
0.02%
0.02%
0.02%
0.02%
0.02%
0.02%
0.02%
0.02%
0.02%
0.02%
0.02%
0.02%
0.02%
0.02%
0.02%
0.02%
0.02%
0.02%
0.02%
0.02%
0.02%
0.02%
0.02%
0.02%
0.02%
0.02%
0.02%
0.02%
0.02%
0.02%
0.02%
0.02%
0.02%
0.02%
0.02%
0.02%
0.02%
0.02%
0.02%
0.02%
0.02%
0.02%
0.01%
0.01%
0.01%
0.01%
0.01%

0.01%
0.01%
0.01%
0.01%
0.01%
0.01%
0.01%
0.01%
0.01%
0.01%
0.01%
0.01%
0.01%
0.01%
0.01%
0.01%
0.01%
0.01%
0.01%
0.01%
0.01%
0.01%
0.01%
0.01%
0.01%
0.01%
0.01%
0.01%
0.01%
0.01%
0.01%
0.01%
0.01%
0.01%
0.01%
0.01%
0.01%
0.01%
0.01%
0.01%
0.01%
0.01%
0.01%
0.01%
0.01%
0.01%
0.01%
0.01%
0.01%
0.01%

0.01%
0.01%
0.01%
0.01%
0.01%
0.01%
0.01%
0.01%
0.01%
0.01%
0.01%
0.01%
0.01%
0.01%
0.01%
0.01%
0.01%
0.01%
0.01%
0.01%
0.01%
0.01%
0.01%
0.01%
0.01%
0.01%
0.01%
0.01%
0.01%
0.01%
0.01%
0.01%
0.01%
0.01%
0.01%
0.01%
0.01%
0.01%
0.01%
0.01%
0.01%
0.01%
0.01%
0.01%
0.01%
0.01%
0.01%
0.01%
0.01%
0.01%

0.01%
0.01%
0.01%
0.01%
0.01%
0.01%
0.01%
0.01%
0.01%
0.01%
0.01%
0.01%
0.01%
0.01%
0.01%
0.01%
0.01%
0.01%
0.01%
0.01%
0.01%
0.01%
0.01%
0.01%
0.01%
0.01%
0.01%
0.01%
0.01%
0.01%
0.01%
0.01%
0.01%
0.01%
0.01%
0.01%
0.01%
0.01%
0.01%
0.01%
0.01%
0.01%
0.01%
0.01%
0.01%
0.01%
0.01%
0.01%
0.01%
0.01%

0.01%
0%
0%
0%
0%
0%
0%
0%
0%
0%
0%
0%
0%
0%
0%
0%
0%
0%
0%
0%
0%
0%
0%
0%
0%
0%
0%
0%
0%
0%
0%
0%
0%
0%
0%
0%
0%
0%
0%
0%
0%
0%
0%
0%
0%
0%
0%
0%
0%
0%

0%
0%
0%
0%
0%
0%
0%
0%
0%
0%
0%
0%
0%
0%
0%
0%
0%
0%
0%
0%
0%
0%
0%
0%
0%
0%
0%
0%
0%
0%
0%
0%
0%
0%
0%
0%
0%
0%
0%
0%
0%
0%
0%
0%
0%
0%
0%
0%
0%
0%

0%
0%
0%
0%
0%
0%
0%
0%
0%
0%
0%
0%
0%
0%
0%
0%
0%
0%
0%
0%
0%
0%
0%
0%
0%
0%
0%
0%
0%
0%
0%
0%
0%
0%
0%
0%
0%
0%
0%
0%
0%
0%
0%
0%
0%
0%
0%
0%
0%
0%

0%
0%
0%
0%
0%
0%
0%
0%
0%
0%
0%
