# Supplementary material for: Influence of the Fermented Feed and Vaccination and Their Interaction on Parameters of Large White/Norwegian Landrace Piglets
Source: Animals (Basel). 2020 Jul 15;10(7):1201. doi: 10.3390/ani10071201 (PMC7401620; doi:10.3390/ani10071201)
Supplement: Supplementary file 1 [file animals-10-01201-s001.zip › Table S2 Species SV group before experiment.pdf]

**Species SV group before experiment**	**Number of reads**	**Relative abundance**
Prevotella copri	6777	19.31%
Lactobacillus amylovorus	6385	18.19%
Lactobacillus reuteri	2912	8.3%
Barnesiella intestinihominis	2280	6.5%
Prevotella oris	1013	2.89%
Unclassified	878	2.5%
Prevotella stercorea	802	2.29%
Faecalibacterium prausnitzii	662	1.89%
Catenibacterium mitsuokai	555	1.58%
Escherichia coli	458	1.3%
Anaerobium acetethylicum	450	1.28%
Anaerovibrio lipolyticus	447	1.27%
Alloprevotella rava	345	0.98%
Roseburia faecis	325	0.93%
Prevotella oralis	322	0.92%
Blautia wexlerae	277	0.79%
Megasphaera elsdenii	266	0.76%
Flintibacter butyricus	263	0.75%
Prevotella conceptionensis	256	0.73%
Prevotella brevis	239	0.68%
Bacteroidales oral	207	0.59%
Intestinimonas butyriciproducens	192	0.55%
Oscillibacter ruminantium	183	0.52%
Phascolarctobacterium succinatutens	178	0.51%
Eubacterium eligens	168	0.48%
Oscillospira guilliermondii	164	0.47%
Intestinibacter bartlettii	161	0.46%
Butyricicoccus pullicaecorum	159	0.45%
unclassified Deltaproteobacteria	154	0.44%
unclassified Bacteroidales	150	0.43%
Fusicatenibacter saccharivorans	147	0.42%
Eubacterium rectale	142	0.4%
Coprococcus catus	137	0.39%
Lactobacillus jensenii	132	0.38%
Ruminococcus faecis	123	0.35%
Fournierella massiliensis	121	0.34%
Gemmiger formicilis	119	0.34%
Sporobacter termitidis	117	0.33%
Eubacterium coprostanoligenes	109	0.31%
Paraprevotella clara	109	0.31%
Parabacteroides distasonis	108	0.31%
Murimonas intestini	103	0.29%
unclassified Tannerella	103	0.29%
Lactobacillus crispatus	101	0.29%
Lactobacillus delbrueckii	97	0.28%
Ruminococcus bicirculans	96	0.27%
Clostridium cellulovorans	94	0.27%
Lactobacillus kitasatonis	88	0.25%
Holdemanella biformis	88	0.25%

Terrisporobacter glycolicus	83	0.24%
Anaerostipes butyraticus	82	0.23%
unclassified Prevotella	77	0.22%
Lactobacillus mucosae	76	0.22%
Succinivibrio dextrinosolvens	73	0.21%
unclassified Rikenella	71	0.2%
Ruminococcus torques	67	0.19%
Saccharofermentans acetigenes	63	0.18%
Dorea formicigenerans	60	0.17%
Porphyromonas catoniae	59	0.17%
Blautia obeum	59	0.17%
Roseburia inulinivorans	59	0.17%
Clostridium aldenense	58	0.17%
Ruminiclostridium thermocellum	57	0.16%
Campylobacter lanienae	56	0.16%
Lactobacillus pontis	56	0.16%
unclassified Barnesiella	53	0.15%
Lactobacillus panis	51	0.15%
Clostridium phoceensis	51	0.15%
Intestinimonas timonensis	51	0.15%
Prevotella loescheii	50	0.14%
Ruminococcus flavefaciens	49	0.14%
Eubacterium ramulus	48	0.14%
Prevotella dentalis	48	0.14%
Hungatella hathewayi	48	0.14%
Blautia schinkii	47	0.13%
Mogibacterium diversum	47	0.13%
Blautia producta	46	0.13%
Blautia glucerasea	46	0.13%
Anaerotaenia torta	46	0.13%
Barnesiella viscericola	46	0.13%
Lactobacillus frumenti	46	0.13%
Lactobacillus helveticus	45	0.13%
Prevotella genomosp.	45	0.13%
Desulfovibrio fairfieldensis	45	0.13%
Eubacteriaceae oral	43	0.12%
Blautia stercoris	43	0.12%
Coprococcus comes	42	0.12%
Ruminococcus bromii	42	0.12%
Christensenella minuta	39	0.11%
Prevotella paludivivens	38	0.11%
Papillibacter cinnamivorans	37	0.11%
Candidatus Dorea	37	0.11%
Ruminococcus lactaris	36	0.1%
Desulfovibrio piger	35	0.1%
unclassified Lachnospiraceae	35	0.1%
Candidatus Soleaferrea	35	0.1%
Dorea longicatena	33	0.09%
Anaerovorax odorimutans	33	0.09%
Sphaerochaeta coccoides	33	0.09%

Gracilibacter thermotolerans	33	0.09%
Prevotella buccae	33	0.09%
Bacteroidales genomosp.	31	0.09%
Clostridium polysaccharolyticum	31	0.09%
Clostridium aminobutyricum	31	0.09%
Acetivibrio ethanolgignens	30	0.09%
Eubacterium oxidoreducens	30	0.09%
Clostridium chartatabidum	30	0.09%
Blautia massiliensis	28	0.08%
Clostridium asparagiforme	28	0.08%
Eubacterium hallii	28	0.08%
unclassified Erysipelotrichaceae	25	0.07%
Blautia luti	25	0.07%
Agathobacter ruminis	25	0.07%
Parabacteroides goldsteinii	24	0.07%
Eisenbergiella tayi	24	0.07%
Clostridium leptum	24	0.07%
Ruminococcus callidus	23	0.07%
Clostridium saccharolyticum	23	0.07%
Selenomonas ruminantium	23	0.07%
unclassified Prevotellaceae	23	0.07%
Sphaerochaeta pleomorpha	23	0.07%
unclassified Turicibacter	22	0.06%
Collinsella aerofaciens	22	0.06%
Prevotella shahii	22	0.06%
Eubacterium ruminantium	22	0.06%
Acidaminobacter hydrogenoformans	21	0.06%
Prevotella salivae	21	0.06%
Intestinimonas massiliensis	21	0.06%
Oribacterium sinus	20	0.06%
Mucispirillum schaedleri	20	0.06%
unclassified Ruminococcaceae	20	0.06%
Eubacterium infirmum	20	0.06%
Clostridium populeti	20	0.06%
Lactobacillus acidophilus	20	0.06%
Falcatimonas natans	19	0.05%
Mitsuokella jalaludinii	19	0.05%
cyanobacterium enrichment	19	0.05%
Bacteroides stercoris	19	0.05%
Parabacteroides chinchillae	19	0.05%
Prevotella maculosa	18	0.05%
Clostridium celatum	18	0.05%
Intestinimonas gabonensis	18	0.05%
Treponema bryantii	18	0.05%
Prevotella ruminicola	18	0.05%
Asaccharospora irregularis	18	0.05%
Hespellia porcina	17	0.05%
Peptococcus simiae	17	0.05%
Butyrivibrio fibrisolvens	17	0.05%
Clostridium hylemonae	16	0.05%

unclassified Porphyromonadaceae	16	0.05%
Fibrobacter intestinalis	16	0.05%
Blautia faecis	16	0.05%
Robinsoniella peoriensis	16	0.05%
Anaerostipes hadrus	16	0.05%
Brassicibacter thermophilus	16	0.05%
unclassified Clostridium	16	0.05%
Paludibacter propionicigenes	16	0.05%
unclassified Candidatus Glomeribacter	15	0.04%
Natranaerovirga pectinivora	15	0.04%
Denitrobacterium detoxificans	15	0.04%
Lactobacillus johnsonii	15	0.04%
Herbinix luporum	14	0.04%
Bacteroides caecicola	14	0.04%
Parasporobacterium paucivorans	14	0.04%
Lactobacillus antri	14	0.04%
unclassified Anaerovibrio	14	0.04%
Methanobrevibacter smithii	14	0.04%
Ruminococcus gnavus	14	0.04%
Caloramator fervidus	14	0.04%
Desulfotomaculum guttoideum	14	0.04%
Ruminococcus albus	13	0.04%
Eubacterium desmolans	13	0.04%
Pseudomonas fluorescens	13	0.04%
methanogenic archaeon	13	0.04%
Roseburia intestinalis	13	0.04%
Candidatus Treponema	13	0.04%
Pseudoflavonifractor capillosus	12	0.03%
Anaerobacterium chartisolvens	12	0.03%
Eubacterium siraeum	12	0.03%
Clostridium fimetarium	12	0.03%
Kluyvera georgiana	11	0.03%
Roseburia hominis	11	0.03%
Enorma massiliensis	11	0.03%
Holdemania filiformis	11	0.03%
unclassified Lactobacillaceae	11	0.03%
Paludibacter jiangxiensis	11	0.03%
Lactobacillus vaginalis	11	0.03%
unclassified Wautersiella	11	0.03%
Methylocystis rosea	11	0.03%
Clostridium oroticum	11	0.03%
Clostridium methylpentosum	10	0.03%
unclassified Mollicutes	10	0.03%
Lactobacillus amylolyticus	10	0.03%
Olsenella scatoligenes	10	0.03%
Pleomorphochaeta multiformis	10	0.03%
Erysipelothrix inopinata	10	0.03%
Mobilitalea sibirica	10	0.03%
unclassified Clostridiales	10	0.03%
Alloprevotella tannerae	10	0.03%

Parabacteroides merdae	10	0.03%
Clostridium symbiosum	10	0.03%
Prevotella scopos	9	0.03%
Anaerosporobacter mobilis	9	0.03%
unclassified Planctomycetales	9	0.03%
Clostridium quinii	9	0.03%
Treponema berlinense	9	0.03%
Anaeromassilibacillus senegalensis	9	0.03%
Acetivibrio cellulolyticus	9	0.03%
Caminicella sporogenes	9	0.03%
Clostridium clostridioforme	9	0.03%
Vallitalea pronyensis	8	0.02%
unclassified Lactobacillus	8	0.02%
Prevotella bivia	8	0.02%
Lachnospira pectinoschiza	8	0.02%
Lutispora thermophila	8	0.02%
Anaerocolumna cellulosilytica	8	0.02%
Clostridium tepidiprofundi	8	0.02%
Prevotella dentasini	8	0.02%
Clostridium fusiformis	8	0.02%
Ruminococcus champanellensis	8	0.02%
unclassified Paludibacter	7	0.02%
Desulfotomaculum nigrificans	7	0.02%
unclassified Roseburia	7	0.02%
Desulfovibrio desulfuricans	7	0.02%
Clostridium cellobioparum	7	0.02%
Anaerostipes rhamnosivorans	7	0.02%
Bacteroides salanitronis	7	0.02%
Flavonifractor plautii	7	0.02%
Clostridium xylanolyticum	7	0.02%
unclassified Enterococcus	7	0.02%
Oscillibacter valericigenes	7	0.02%
Bacteroides timonensis	7	0.02%
Faecalicoccus acidiformans	7	0.02%
Coprococcus eutactus	7	0.02%
Turicibacter sanguinis	7	0.02%
Clostridium longisporum	7	0.02%
Lactobacillus secaliphilus	7	0.02%
Eubacterium rangiferina	7	0.02%
Clostridium lactatifermentans	7	0.02%
Ruthenibacterium lactatiformans	7	0.02%
Lactobacillus hamsteri	7	0.02%
Solobacterium moorei	7	0.02%
unclassified Clostridia	6	0.02%
unclassified Alloprevotella	6	0.02%
Succiniclasticum ruminis	6	0.02%
Clostridium cellulolyticum	6	0.02%
Clostridium lavalense	6	0.02%
Bacteroides intestinalis	6	0.02%
unclassified Eubacterium	6	0.02%

Geosporobacter ferrireducens	6	0.02%
Escherichia albertii	6	0.02%
Olivibacter sitiensis	6	0.02%
Selenomonas bovis	6	0.02%
Bacteroides faecis	6	0.02%
Eubacterium sulci	6	0.02%
Eubacterium contortum	6	0.02%
unclassified Bacteroides	6	0.02%
Marvinbryantia formatexigens	6	0.02%
Clostridium sphenoides	6	0.02%
Howardella ureilytica	5	0.01%
Bacteroides uniformis	5	0.01%
Clostridium indolis	5	0.01%
Catabacter hongkongensis	5	0.01%
Desulfotomaculum halophilum	5	0.01%
Ruminococcus gauvreauii	5	0.01%
Propionispira arcuata	5	0.01%
Bacteroides caecigallinarum	5	0.01%
Prevotella enoeca	5	0.01%
Bacteroides zoogleoformans	5	0.01%
Parasutterella secunda	5	0.01%
Prevotella bryantii	5	0.01%
Clostridium scindens	5	0.01%
Eubacterium cellulosolvens	5	0.01%
Hallella seregens	5	0.01%
Porphyromonas pasteri	5	0.01%
Clostridium papyrosolvens	4	0.01%
Bacteroides caccae	4	0.01%
Prevotella albensis	4	0.01%
Prevotella baroniae	4	0.01%
Sutterella stercoricanis	4	0.01%
Caloranaerobacter azorensis	4	0.01%
Lachnospira multipara	4	0.01%
Clostridium celerecrescens	4	0.01%
Clostridium neopropionicum	4	0.01%
Clostridium colicanis	4	0.01%
Lactobacillus rogosae	4	0.01%
Kosakonia sacchari	4	0.01%
Clostridium bovipellis	4	0.01%
Romboutsia sedimentorum	4	0.01%
Coprobacillus cateniformis	4	0.01%
Clostridium viride	4	0.01%
Clostridium glycyrrhizinilyticum	4	0.01%
Oligosphaera ethanolica	4	0.01%
Elbe River	4	0.01%
Prevotella buccalis	4	0.01%
Clostridium aerotolerans	4	0.01%
Bacteroides oleiciplenus	4	0.01%
Propionispira paucivorans	4	0.01%
unclassified Methanobrevibacter	4	0.01%

Acetanaerobacterium elongatum	4	0.01%
Bacteroides clarus	4	0.01%
Lactonifactor longoviformis	4	0.01%
Anaeroplasma abactoclasticum	4	0.01%
Macellibacteroides fermentans	4	0.01%
Megasphaera hominis	3	0.01%
unclassified Bifidobacterium	3	0.01%
Ethanoligenens harbinense	3	0.01%
Abyssivirga alkaniphila	3	0.01%
Bacteroides pectinophilus	3	0.01%
Clostridium hveragerdense	3	0.01%
alpha proteobacterium	3	0.01%
Clostridium colinum	3	0.01%
Salmonella enterica	3	0.01%
Bacteroides barnesiae	3	0.01%
Proteiniborus ethanoligenes	3	0.01%
unclassified Bacteroidaceae	3	0.01%
Lachnoanaerobaculum umeaense	3	0.01%
Anaerotruncus colihominis	3	0.01%
Clostridium clariflavum	3	0.01%
Prevotella fusca	3	0.01%
Alkaliphilus oremlandii	3	0.01%
Lactobacillus tucceti	3	0.01%
Asteroleplasma anaerobium	3	0.01%
unclassified Betaproteobacteria	3	0.01%
Cellulosilyticum ruminicola	3	0.01%
unclassified Veillonellaceae	3	0.01%
Lactobacillus fermentum	3	0.01%
Escherichia fergusonii	3	0.01%
Defluviitalea phaphyphila	3	0.01%
unclassified Spirochaetia	3	0.01%
Prevotella histicola	3	0.01%
Lactobacillus gasseri	3	0.01%
Anaerocolumna xylanovorans	3	0.01%
unclassified Treponema	3	0.01%
Prevotella amnii	3	0.01%
Candidatus Izimaplasma	3	0.01%
unclassified Oscillospira	3	0.01%
Acidaminococcus fermentans	2	0.01%
Garciella nitratireducens	2	0.01%
Eubacterium tenue	2	0.01%
Lactobacillus gallinarum	2	0.01%
unclassified Fusobacterium	2	0.01%
Enterorhabdus caecimuris	2	0.01%
Enterorhabdus mucosicola	2	0.01%
Peptostreptococcus stomatis	2	0.01%
Paraclostridium benzoelyticum	2	0.01%
Paeniclostridium sordellii	2	0.01%
Porphyromonas cangingivalis	2	0.01%
Clostridium cocleatum	2	0.01%

Bacteroides cellulosilyticus	2	0.01%
Caproiciproducens galactitolivorans	2	0.01%
unclassified Papillibacter	2	0.01%
Lactobacillus coleohominis	2	0.01%
Desulfotomaculum tongense	2	0.01%
Clostridium straminisolvens	2	0.01%
Campylobacter jejuni	2	0.01%
Clostridium amylolyticum	2	0.01%
Bacillus pumilus	2	0.01%
Paraeggerthella hongkongensis	2	0.01%
Prevotella multiformis	2	0.01%
Candidatus Methanomethylophilus	2	0.01%
Lactobacillus taiwanensis	2	0.01%
Pediococcus ethanolidurans	2	0.01%
Clostridium cadaveris	2	0.01%
Erysipelothrix rhusiopathiae	2	0.01%
Helicobacter rodentium	2	0.01%
Clostridium sulfidigenes	2	0.01%
Bacteroides heparinolyticus	2	0.01%
Corynebacterium provencense	2	0.01%
Lactobacillus rodentium	2	0.01%
Parabacteroides gordonii	2	0.01%
unclassified Desulfonispora	2	0.01%
Porphyromonas pogonae	2	0.01%
Lactobacillus casei	2	0.01%
Bacteroides salyersiae	2	0.01%
Oxobacter pfennigii	2	0.01%
Prevotella micans	2	0.01%
Anaerofustis stercorihominis	2	0.01%
Lachnoanaerobaculum saburreum	2	0.01%
Desulfitobacterium chlororespirans	2	0.01%
Eubacterium plexicaudatum	2	0.01%
unclassified Anaerovorax	2	0.01%
Thermotalea metallivorans	2	0.01%
Prevotella denticola	2	0.01%
Clostridium caenicola	2	0.01%
Caldicoprobacter algeriensis	2	0.01%
Butyricimonas virosa	2	0.01%
Bacteroides coprocola	2	0.01%
Sedimentibacter hydroxybenzoicus	2	0.01%
Fusobacterium perfoetens	2	0.01%
Candidatus Heliomonas	2	0.01%
Anaeroplasma bactoclasticum	2	0.01%
Bacteroides nordii	2	0.01%
Cellulosibacter alkalithermophilus	2	0.01%
Clostridium hiranonis	2	0.01%
unclassified Ruminococcus	2	0.01%
unclassified Syntrophococcus	2	0.01%
Peptococcus niger	2	0.01%
Gorbachella massiliensis	2	0.01%

unclassified Bulleidia	2	0.01%
Anaerobiospirillum succiniciproducens	2	0.01%
Lachnoanaerobaculum cf.	1	0%
Eubacterium xylanophilum	1	0%
Sphaerochaeta globosa	1	0%
Spiroplasma lampyridicola	1	0%
Ornatilinea apprima	1	0%
Parapedobacter pyrenivorans	1	0%
Parvibacter caecicola	1	0%
Clostridioides difficile	1	0%
unclassified Pedobacter	1	0%
Fusobacterium periodonticum	1	0%
Lysobacter dokdonensis	1	0%
Clostridium sufflavum	1	0%
Cellulomonas terrae	1	0%
Bifidobacterium breve	1	0%
Rarimicrobium hominis	1	0%
Helicobacter canadensis	1	0%
unclassified Haloplasmataceae	1	0%
Dialister succinatiphilus	1	0%
unclassified Sphingobacterium	1	0%
Lactobacillus acidifarinae	1	0%
Lactobacillus hilgardii	1	0%
Clostridium tyrobutyricum	1	0%
Herbivorax saccincola	1	0%
Tepidibacter mesophilus	1	0%
Pseudogymnoascus roseus	1	0%
Rubrivivax gelatinosus	1	0%
Clostridium putrefaciens	1	0%
Clostridium thermosuccinogenes	1	0%
unclassified Zoogloea	1	0%
Campylobacter lari	1	0%
Bacteroides ovatus	1	0%
Clostridium aurantibutyricum	1	0%
unclassified Acidimicrobium	1	0%
Sharpea azabuensis	1	0%
gamma proteobacterium	1	0%
Alistipes massiliensis	1	0%
Adlercreutzia equolifaciens	1	0%
Photorhabdus luminescens	1	0%
Anaerocolumna jejuensis	1	0%
Treponema porcinum	1	0%
Lacibacterium aquatile	1	0%
Shigella flexneri	1	0%
unclassified Conexibacter	1	0%
Eisenbergiella massiliensis	1	0%
Clostridium botulinum	1	0%
Clostridium thermopalmarium	1	0%
unclassified Victivallaceae	1	0%
Kiloniella spongiae	1	0%

Megasphaera indica	1	0%
Parabacteroides johnsonii	1	0%
Lactobacillus psittaci	1	0%
unclassified Sphingobium	1	0%
Blautia hydrogenotrophica	1	0%
unclassified Lactobacillales	1	0%
Treponema zioleckii	1	0%
unclassified Clostridiaceae	1	0%
Clostridium sporogenes	1	0%
Pantoea ananatis	1	0%
planctomycete str.	1	0%
Clostridium tarantellae	1	0%
Shigella dysenteriae	1	0%
Bradyrhizobium canariense	1	0%
Prevotella nanceiensis	1	0%
Acidaminococcus intestini	1	0%
Clostridium cellulosi	1	0%
Butyrivibrio crossotus	1	0%
Slackia exigua	1	0%
Clostridium tertium	1	0%
unclassified Megasphaera	1	0%
Subdoligranulum variabile	1	0%
Zoogloea oryzae	1	0%
unclassified Cytophaga	1	0%
Paeniclostridium ghonii	1	0%
Clostridium disporicum	1	0%
Clostridium sartagoforme	1	0%
Catonella morbi	1	0%
Eubacterium uniforme	1	0%
Acetoanaerobium pronyense	1	0%
Flavobacterium johnsoniae	1	0%
Gottschalkia acidurici	1	0%
metal-contaminated soil	1	0%
Lactobacillus porcinae	1	0%
unclassified Staphylococcus	1	0%
unclassified Erysipelothrix	1	0%
Edaphobacter modestus	1	0%
Lactobacillus sakei	1	0%
Clostridium intestinale	1	0%
Defluviitalea raffinosedens	1	0%
Clostridium fallax	1	0%
Eubacterium limosum	1	0%
Ruminobacter amylophilus	1	0%
Prevotella marshii	1	0%
unclassified Bacillales	1	0%
unclassified Subdoligranulum	1	0%
Bifidobacterium longum	1	0%
Bacillus panaciterrae	1	0%
Eubacterium fissicatena	1	0%
unclassified Bacillus	1	0%

Clostridium vulturis	1	0%
Solitalea canadensis	1	0%
Lachnospiraceae oral	1	0%
Jonquetella anthropi	1	0%
Bacteroides luti	1	0%
Clostridium bolteae	1	0%
Bacillus niacini	1	0%
Kribbella swartbergensis	1	0%
Dongia mobilis	1	0%
Dongia rigui	1	0%
Alternaria atra	1	0%
Prevotella timonensis	1	0%
Pedosphaera parvula	1	0%
Pediococcus pentosaceus	1	0%
Helicobacter rappini	1	0%
Solirubrobacter ginsenosidimutans	1	0%
Desulfovibrio intestinalis	1	0%
unclassified Acidobacterium	1	0%
Pseudobutyrivibrio xylanivorans	1	0%
Lactobacillus camelliae	1	0%
Mycobacterium tuberculosis	1	0%
Kiloniella laminariae	1	0%
Pyramidobacter piscolens	1	0%
Tepidimicrobium xylanilyticum	1	0%
Prevotella saccharolytica	1	0%
Bacteroides helcogenes	1	0%
unclassified Tepidibacter	1	0%
Tyzzerella nexilis	1	0%
Bacteroides galacturonicus	1	0%
Carboxylicivirga flava	1	0%
Olsenella umbonata	1	0%
Clostridium aldrichii	1	0%
Methylophilus methylotrophus	1	0%
Erwinia teleogrylli	1	0%
Prevotella melaninogenica	1	0%
unclassified Actinomycetales	1	0%
Ercella succinigenes	1	0%
Dehalobacterium formicoaceticum	1	0%
Defluviitalea saccharophila	1	0%
Faecalicoccus pleomorphus	1	0%
Phenylobacterium composti	1	0%
actinobacterium SCGC	1	0%
Verticillium dahliae	1	0%
unclassified Cryptanaerobacter	1	0%
Clostridium chauvoei	1	0%
Clostridium aestuarii	1	0%
Blautia coccoides	1	0%
unclassified Gammaproteobacteria	1	0%
Saccharomyces cerevisiae	1	0%
Flavitalea populi	1	0%

unclassified Ethanoligenens	1	0%
Acetatifactor muris	1	0%
unclassified Anaerostipes	1	0%
Clostridium isatidis	1	0%
Shuttleworthia satelles	1	0%
Serratia marcescens	1	0%
Zhizhongheella caldifontis	1	0%
Parastreptomyces abscessus	1	0%
Breznakia pachnodae	1	0%
Salinibacillus kushneri	1	0%
Prevotella oulorum	1	0%
Bulleidia extructa	1	0%
Mogibacterium timidum	1	0%
Clostridium ventriculi	1	0%
Bacteroides acidifaciens	1	0%
unclassified Enterobacteriaceae	1	0%
Fibrobacter succinogenes	1	0%
Pseudomonas savastanoi	1	0%
denitrifying Fe-oxidizing	1	0%
Massilia timonae	1	0%
Cytophaga xylanolytica	1	0%
unclassified Oribacterium	1	0%
Dielma fastidiosa	1	0%
Treponema brennaborense	1	0%
Alternaria alternata	1	0%
unclassified Bacteroidia	1	0%
Kitasatospora mediocidica	1	0%
