# Supplementary material for: Influence of the Fermented Feed and Vaccination and Their Interaction on Parameters of Large White/Norwegian Landrace Piglets
Source: Animals (Basel). 2020 Jul 15;10(7):1201. doi: 10.3390/ani10071201 (PMC7401620; doi:10.3390/ani10071201)
Supplement: Supplementary file 1 [file animals-10-01201-s001.zip › Table S3 Species RFnonV group before experiment.pdf]

Species RFnonV group before experiment	Number of reads	Relative abundance
Prevotella copri	10791	29.53%
Lactobacillus amylovorus	7792	21.32%
Roseburia faecis	1118	3.06%
Unclassified	970	2.65%
Bifidobacterium boum	931	2.55%
Barnesiella intestinihominis	868	2.38%
Megasphaera elsdenii	784	2.15%
Eubacterium rectale	768	2.1%
Faecalibacterium prausnitzii	678	1.86%
Prevotella oris	555	1.52%
Anaerovibrio lipolyticus	527	1.44%
Clostridium cellulovorans	419	1.15%
Prevotella stercorea	371	1.02%
Alloprevotella rava	356	0.97%
Selenomonas bovis	338	0.92%
Bifidobacterium thermophilum	334	0.91%
Prevotella brevis	293	0.8%
Terrisporobacter glycolicus	264	0.72%
Prevotella oralis	227	0.62%
Sporobacter termitidis	222	0.61%
Lactobacillus panis	207	0.57%
Clostridium chartatabidum	202	0.55%
Phascolarctobacterium succinatutens	179	0.49%
Catenibacterium mitsuokai	172	0.47%
Ruminococcus bromii	155	0.42%
Lactobacillus reuteri	150	0.41%
Bacteroidales oral	147	0.4%
Lactobacillus crispatus	138	0.38%
Ruminococcus faecis	125	0.34%
Fournierella massiliensis	123	0.34%
Lactobacillus delbrueckii	122	0.33%
unclassified Bacteroidales	118	0.32%
Paraprevotella clara	115	0.31%
Intestinibacter bartlettii	109	0.3%
Lactobacillus kitasatonis	105	0.29%
Lactobacillus pontis	102	0.28%
Flintibacter butyricus	91	0.25%
Bifidobacterium pseudolongum	90	0.25%
Clostridium celatum	85	0.23%
Blautia glucerasea	81	0.22%
Lactobacillus jensenii	81	0.22%
Prevotella salivae	81	0.22%
Prevotella paludivivens	77	0.21%
Eubacterium eligens	76	0.21%
Blautia wexlerae	76	0.21%
Blautia obeum	76	0.21%
Bifidobacterium breve	76	0.21%
Murimonas intestini	75	0.21%
Fusicatenibacter saccharivorans	74	0.2%

Parabacteroides distasonis	73	0.2%
Roseburia inulinivorans	71	0.19%
unclassified Tannerella	69	0.19%
Gemmiger formicilis	68	0.19%
Oscillibacter ruminantium	63	0.17%
Collinsella aerofaciens	62	0.17%
Escherichia coli	61	0.17%
Desulfovibrio piger	59	0.16%
Bifidobacterium choerinum	58	0.16%
Ruminococcus flavefaciens	58	0.16%
Butyrivibrio fibrisolvens	55	0.15%
unclassified Prevotella	55	0.15%
Campylobacter lanienae	54	0.15%
Eubacterium coprostanoligenes	53	0.15%
Butyricicoccus pullicaecorum	53	0.15%
Ruminiclostridium thermocellum	53	0.15%
Saccharofermentans acetigenes	48	0.13%
Oscillospira guilliermondii	46	0.13%
Clostridium quinii	46	0.13%
Ruminococcus torques	43	0.12%
Asteroleplasma anaerobium	42	0.11%
unclassified Lachnospiraceae	40	0.11%
Mitsuokella jalaludinii	39	0.11%
Intestinimonas butyriciproducens	39	0.11%
Romboutsia sedimentorum	39	0.11%
Parabacteroides chinchillae	38	0.1%
Christensenella minuta	37	0.1%
Lactobacillus helveticus	37	0.1%
Clostridium longisporum	37	0.1%
Bacteroides stercoris	36	0.1%
Coprococcus comes	35	0.1%
Prevotella dentalis	34	0.09%
Erysipelothrix inopinata	33	0.09%
Bifidobacterium longum	32	0.09%
Eubacterium ramulus	31	0.08%
Blautia massiliensis	31	0.08%
Turicibacter sanguinis	31	0.08%
Agathobacter ruminis	30	0.08%
Lactobacillus acidophilus	29	0.08%
Prevotella ruminicola	28	0.08%
Lactobacillus frumenti	28	0.08%
Eubacterium desmolans	28	0.08%
Porphyromonas catoniae	28	0.08%
Hungatella hathewayi	28	0.08%
Lactobacillus johnsonii	27	0.07%
Eubacterium hallii	27	0.07%
Anaerobacterium chartisolvens	26	0.07%
Mogibacterium diversum	26	0.07%
Holdemanella biformis	25	0.07%
Blautia schinkii	25	0.07%

Intestinimonas timonensis	25	0.07%
Eubacterium oxidoreducens	24	0.07%
unclassified Prevotellaceae	24	0.07%
Blautia stercoris	24	0.07%
Sutterella stercoricanis	23	0.06%
Anaerovorax odorimutans	23	0.06%
Desulfovibrio fairfieldensis	22	0.06%
Dorea longicatena	22	0.06%
Eubacterium ruminantium	22	0.06%
unclassified Ruminococcaceae	22	0.06%
Holdemania filiformis	22	0.06%
Clostridium phoceensis	21	0.06%
Acetivibrio ethanolgignens	20	0.05%
Dorea formicigenerans	20	0.05%
Clostridium populeti	20	0.05%
Candidatus Soleaferrea	20	0.05%
unclassified Barnesiella	20	0.05%
Sharpea azabuensis	20	0.05%
unclassified Rikenella	20	0.05%
Intestinimonas massiliensis	20	0.05%
unclassified Deltaproteobacteria	19	0.05%
Oribacterium sinus	19	0.05%
Clostridium aldenense	19	0.05%
Clostridium bovipellis	19	0.05%
Denitrobacterium detoxificans	19	0.05%
unclassified Clostridium	19	0.05%
Anaerobium acetethylicum	18	0.05%
Bifidobacterium pullorum	18	0.05%
Prevotella genomosp.	18	0.05%
Clostridium symbiosum	18	0.05%
Clostridium cellobioparum	17	0.05%
unclassified Anaerovibrio	17	0.05%
Clostridium tepidiprofundi	17	0.05%
Olivibacter sitiensis	16	0.04%
Anaerotaenia torta	16	0.04%
Bifidobacterium thermacidophilum	16	0.04%
Clostridium polysaccharolyticum	16	0.04%
Bifidobacterium scardovii	16	0.04%
Bacteroidales genomosp.	16	0.04%
Methylocystis rosea	16	0.04%
Prevotella buccae	16	0.04%
Pyramidobacter piscolens	15	0.04%
unclassified Bifidobacterium	15	0.04%
Bacteroides uniformis	15	0.04%
Blautia faecis	15	0.04%
Barnesiella viscericola	15	0.04%
Solobacterium moorei	15	0.04%
Clostridium colicanis	14	0.04%
Prevotella conceptionensis	14	0.04%
Propionispira arcuata	14	0.04%

Ruminococcus bicirculans	14	0.04%
Prevotella loescheii	14	0.04%
Clostridium oroticum	14	0.04%
Coprococcus catus	14	0.04%
Mitsuokella multacida	14	0.04%
Clostridium aminobutyricum	14	0.04%
unclassified Clostridiales	14	0.04%
Ruthenibacterium lactatiformans	13	0.04%
Bacteroides intestinalis	13	0.04%
Roseburia hominis	13	0.04%
Prevotella shahii	13	0.04%
Falcatimonas natans	13	0.04%
Eubacterium infirmum	13	0.04%
Clostridium tarantellae	13	0.04%
Eubacteriaceae oral	12	0.03%
Brassicibacter thermophilus	12	0.03%
Parabacteroides goldsteinii	12	0.03%
Roseburia intestinalis	12	0.03%
Gracilibacter thermotolerans	12	0.03%
Succinivibrio dextrinosolvens	12	0.03%
Cloacibacillus porcorum	12	0.03%
Asaccharospora irregularis	12	0.03%
Candidatus Treponema	12	0.03%
Ruminococcus lactaris	12	0.03%
Hespellia porcina	12	0.03%
Clostridium amylolyticum	11	0.03%
Anaeromassilibacillus senegalensis	11	0.03%
Intestinimonas gabonensis	11	0.03%
Bifidobacterium kashiwanohense	11	0.03%
unclassified Porphyromonadaceae	11	0.03%
Acetanaerobacterium elongatum	11	0.03%
Candidatus Dorea	11	0.03%
Coprococcus eutactus	11	0.03%
Selenomonas sputigena	10	0.03%
Prevotella amnii	10	0.03%
Clostridium leptum	10	0.03%
Mucispirillum schaedleri	10	0.03%
Caloramator fervidus	9	0.02%
Clostridium tertium	9	0.02%
Alloprevotella tannerae	9	0.02%
Bacteroides salyersiae	9	0.02%
Megasphaera hominis	9	0.02%
unclassified Paludibacter	9	0.02%
Bacteroides caecicola	9	0.02%
Bacteroides caecigallinarum	8	0.02%
Clostridium paraputrificum	8	0.02%
unclassified Erysipelotrichaceae	8	0.02%
Lactobacillus hamsteri	8	0.02%
Bacteroides zoogleoformans	8	0.02%
Clostridium glycyrrhizinilyticum	8	0.02%

Eisenbergiella tayi	8	0.02%
Bifidobacterium animalis	8	0.02%
Clostridium saccharolyticum	8	0.02%
Clostridium cellulolyticum	8	0.02%
Sphaerochaeta coccoides	8	0.02%
Bifidobacterium tissieri	8	0.02%
methanogenic archaeon	8	0.02%
Lactobacillus coleohominis	8	0.02%
unclassified Alloprevotella	8	0.02%
Clostridium lavalense	8	0.02%
Selenomonas ruminantium	7	0.02%
Treponema brennaborense	7	0.02%
Ruminococcus callidus	7	0.02%
Clostridium methylpentosum	7	0.02%
Clostridium clostridioforme	7	0.02%
Bacteroides helcogenes	7	0.02%
Oscillibacter valericigenes	7	0.02%
Enorma massiliensis	7	0.02%
Bacteroides vulgatus	7	0.02%
Clostridium asparagiforme	7	0.02%
Acetivibrio cellulolyticus	7	0.02%
Anaerocolumna cellulosilytica	7	0.02%
Prevotella scopos	7	0.02%
Vallitalea pronyensis	7	0.02%
Peptococcus simiae	7	0.02%
Prevotella bivia	7	0.02%
Prevotella fusca	7	0.02%
Eubacterium rangiferina	7	0.02%
Lachnospira pectinoschiza	7	0.02%
Alistipes shahii	7	0.02%
Clostridium straminisolvens	7	0.02%
Clostridium xylanolyticum	7	0.02%
unclassified Bacteroides	6	0.02%
Prevotella multiformis	6	0.02%
Treponema berlinense	6	0.02%
Elbe River	6	0.02%
Lactobacillus secaliphilus	6	0.02%
Abyssivirga alkaniphila	6	0.02%
Acidaminococcus fermentans	6	0.02%
Clostridium hveragerdense	6	0.02%
Butyricimonas virosa	6	0.02%
Clostridium butyricum	6	0.02%
Paludibacter jiangxiensis	6	0.02%
Clostridium lactatifermentans	6	0.02%
unclassified Acetivibrio	6	0.02%
Parabacteroides merdae	6	0.02%
Treponema porcinum	6	0.02%
Clostridium disporicum	5	0.01%
Olsenella scatoligenes	5	0.01%
Bacteroides coprocola	5	0.01%

Prevotella maculosa	5	0.01%
Lactobacillus mucosae	5	0.01%
Clostridium aerotolerans	5	0.01%
Clostridium botulinum	5	0.01%
Papillibacter cinnamivorans	5	0.01%
Clostridium clariflavum	5	0.01%
Lactobacillus fermentum	5	0.01%
Paludibacter propionicigenes	5	0.01%
Natranaerovirga pectinivora	5	0.01%
Lactobacillus amylolyticus	5	0.01%
Paraclostridium benzoelyticum	5	0.01%
Oligosphaera ethanolica	5	0.01%
Enterorhabdus mucosicola	5	0.01%
Fibrobacter intestinalis	5	0.01%
Oceanirhabdus sediminicola	5	0.01%
Bacteroides salanitronis	5	0.01%
Eubacterium contortum	5	0.01%
Herbinix luporum	5	0.01%
Blautia hydrogenotrophica	4	0.01%
Catabacter hongkongensis	4	0.01%
Blautia luti	4	0.01%
unclassified Mollicutes	4	0.01%
Lactobacillus psittaci	4	0.01%
Parabacteroides johnsonii	4	0.01%
Blautia producta	4	0.01%
unclassified Asteroleplasma	4	0.01%
Methanobrevibacter smithii	4	0.01%
Clostridium cadaveris	4	0.01%
Eubacterium siraeum	4	0.01%
Flavonifractor plautii	4	0.01%
Clostridium putrefaciens	4	0.01%
Campylobacter hyointestinalis	4	0.01%
Clostridium papyrosolvens	4	0.01%
Bifidobacterium callitrichos	4	0.01%
Lactobacillus rogosae	4	0.01%
Bifidobacterium pseudocatenulatum	4	0.01%
Eubacterium cellulosolvens	4	0.01%
Faecalicoccus acidiformans	4	0.01%
Methylocystis bryophila	4	0.01%
Propionispira paucivorans	4	0.01%
Clostridium scindens	4	0.01%
cyanobacterium enrichment	4	0.01%
Syntrophococcus sucromutans	4	0.01%
Bacteroides oleiciplenus	4	0.01%
Prevotella albensis	4	0.01%
Clostridium baratii	4	0.01%
Anaerostipes butyraticus	4	0.01%
unclassified Wautersiella	4	0.01%
Kluyvera georgiana	4	0.01%
Pseudomonas fluorescens	4	0.01%

Desulfovibrio desulfuricans	4	0.01%
Clostridium sartagoforme	4	0.01%
Pseudoflavonifractor capillosus	4	0.01%
Ethanoligenens harbinense	3	0.01%
Ruminococcus gnavus	3	0.01%
Clostridium aminophilum	3	0.01%
unclassified Petrimonas	3	0.01%
Bacteroides heparinolyticus	3	0.01%
Anaerostipes hadrus	3	0.01%
Robinsoniella peoriensis	3	0.01%
Bacteroides cellulosilyticus	3	0.01%
Bacteroides gallinaceum	3	0.01%
Lactobacillus gallinarum	3	0.01%
Bacteroides barnesiae	3	0.01%
Pseudobutyrivibrio ruminis	3	0.01%
unclassified Enterococcus	3	0.01%
Bacteroides thetaiotaomicron	3	0.01%
unclassified Clostridia	3	0.01%
Bacteroides plebeius	3	0.01%
Ruminococcus champanellensis	3	0.01%
Bifidobacterium bombi	3	0.01%
Sphaerochaeta pleomorpha	3	0.01%
Stomatobaculum longum	3	0.01%
unclassified Lactobacillus	3	0.01%
Prevotella dentasini	3	0.01%
Marvinbryantia formatexigens	3	0.01%
Parasporobacterium paucivorans	3	0.01%
unclassified Bulleidia	3	0.01%
Clostridium hiranonis	3	0.01%
Caminicella sporogenes	3	0.01%
Acidaminococcus intestini	3	0.01%
Clostridium fusiformis	3	0.01%
unclassified Planctomycetales	3	0.01%
unclassified Pyramidobacter	3	0.01%
Acidaminobacter hydrogenoformans	3	0.01%
unclassified Lactobacillaceae	3	0.01%
Olsenella umbonata	3	0.01%
Bacteroides caccae	3	0.01%
Ruminococcus albus	3	0.01%
Bulleidia extructa	3	0.01%
Bacteroides timonensis	3	0.01%
unclassified Catonella	3	0.01%
Prevotella baroniae	3	0.01%
unclassified Eubacterium	3	0.01%
Corynebacterium provencense	3	0.01%
Parvibacter caecicola	3	0.01%
Geosporobacter ferrireducens	2	0.01%
Clostridium fimetarium	2	0.01%
Adlercreutzia equolifaciens	2	0.01%
Methylosinus trichosporium	2	0.01%

Clostridium sphenoides	2	0.01%
Eggerthella lenta	2	0.01%
Escherichia albertii	2	0.01%
Dielma fastidiosa	2	0.01%
unclassified Bacteroidia	2	0.01%
Eisenbergiella massiliensis	2	0.01%
Alistipes putredinis	2	0.01%
Lactobacillus gasseri	2	0.01%
Nocardioidaceae str.	2	0.01%
Bifidobacterium adolescentis	2	0.01%
Methanosphaera cuniculi	2	0.01%
Holdemania massiliensis	2	0.01%
Treponema parvum	2	0.01%
Terrisporobacter mayombei	2	0.01%
Geosporobacter subterraneus	2	0.01%
Vitis hybrid	2	0.01%
Bacillus pumilus	2	0.01%
Prevotella nigrescens	2	0.01%
Eubacterium minutum	2	0.01%
Lachnoclostridium phytofermentans	2	0.01%
Eubacterium xylanophilum	2	0.01%
Lachnoanaerobaculum saburreum	2	0.01%
Lutispora thermophila	2	0.01%
Mageeibacillus indolicus	2	0.01%
delta proteobacterium	2	0.01%
Anaerotruncus colihominis	2	0.01%
Prevotella melaninogenica	2	0.01%
unclassified Roseburia	2	0.01%
Lachnoanaerobaculum umeaense	2	0.01%
Litorilinea aerophila	2	0.01%
Clostridioides difficile	2	0.01%
Anaerobiospirillum succiniciproducens	2	0.01%
Bacteroides dorei	2	0.01%
Clostridium intestinale	2	0.01%
unclassified Bacillus	2	0.01%
Olsenella profusa	2	0.01%
Lachnospira multipara	2	0.01%
Clostridium moniliforme	2	0.01%
Bacillus nealsonii	2	0.01%
Desulfotomaculum nigrificans	2	0.01%
Prevotella denticola	2	0.01%
Blautia hansenii	2	0.01%
Lactobacillus agilis	2	0.01%
Clostridium sulfidigenes	2	0.01%
alpha proteobacterium	2	0.01%
Paraeggerthella hongkongensis	2	0.01%
Clostridium chauvoei	2	0.01%
Peptococcus niger	2	0.01%
Hallella seregens	2	0.01%
Butyrivibrio crossotus	2	0.01%

unclassified Sphingobium	2	0.01%
unclassified Candidatus Glomeribacter	2	0.01%
Enterorhabdus caecimuris	2	0.01%
Pseudarthrobacter sulfonivorans	2	0.01%
Candidatus Methanomethylophilus	2	0.01%
Clostridium isatidis	2	0.01%
Butyricimonas paravirosa	2	0.01%
Odoribacter splanchnicus	2	0.01%
Caproiciproducens galactitolivorans	2	0.01%
Clostridium ventriculi	2	0.01%
Bifidobacterium ruminantium	2	0.01%
Salinibacillus xinjiangensis	2	0.01%
Salmonella enterica	1	0%
Cytophaga xylanolytica	1	0%
Clostridium sardiniense	1	0%
Collinsella intestinalis	1	0%
Stenotrophobacter terrae	1	0%
Lysinibacillus massiliensis	1	0%
Kosakonia sacchari	1	0%
unclassified Thauera	1	0%
Clostridium purinilyticum	1	0%
Hydrogenoanaerobacterium saccharovorans	1	0%
Steroidobacter denitrificans	1	0%
unclassified Dorea	1	0%
unclassified Oscillospira	1	0%
Clostridium tyrobutyricum	1	0%
Gluconobacter oxydans	1	0%
unclassified Methanobrevibacter	1	0%
Woodsholea maritima	1	0%
Coprobacter secundus	1	0%
Clostridium frigoriphilum	1	0%
Sphingomonas sanxanigenens	1	0%
unclassified Turicibacter	1	0%
Clostridium amygdalinum	1	0%
metal-contaminated soil	1	0%
unclassified Actinobacteria	1	0%
Bacteroides massiliensis	1	0%
Terracidiphilus gabretensis	1	0%
Candidatus Udaeobacter	1	0%
Flavobacterium succinicans	1	0%
Sporosarcina luteola	1	0%
Clostridium thermopalmarium	1	0%
Eubacterium sulci	1	0%
Alistipes senegalensis	1	0%
unclassified Papillibacter	1	0%
Prevotella corporis	1	0%
unclassified Victivallaceae	1	0%
Clostridium subterminale	1	0%
Crassaminicella profunda	1	0%
Paraprevotella xylaniphila	1	0%

Sedimentibacter acidaminivorans	1	0%
unclassified Sporobacter	1	0%
Sporosarcina saromensis	1	0%
Clostridium indolis	1	0%
Arcanobacterium hippocoleae	1	0%
Tetrasphaera remsis	1	0%
Chelativorans composti	1	0%
Campylobacter coli	1	0%
Bacteroides pectinophilus	1	0%
Bifidobacterium saeculare	1	0%
Clostridium vincentii	1	0%
Thermotalea metallivorans	1	0%
Bacteroides coprophilus	1	0%
Desulfotomaculum tongense	1	0%
Carboxylicivirga linearis	1	0%
Dethiobacter alkaliphilus	1	0%
Caloranaerobacter azorensis	1	0%
Selenomonas infelix	1	0%
Mobilitalea sibirica	1	0%
Streptomyces yanglinensis	1	0%
Bifidobacterium angulatum	1	0%
unclassified Syntrophococcus	1	0%
Cellulosibacter alkalithermophilus	1	0%
unclassified Methylosinus	1	0%
Clostridium stercorarium	1	0%
Proteiniborus ethanoligenes	1	0%
Bifidobacterium merycicum	1	0%
Lactonifactor longoviformis	1	0%
unclassified Bacteroidaceae	1	0%
Bifidobacterium stellenboschense	1	0%
Clostridium termitidis	1	0%
Drancourtella massiliensis	1	0%
Bacteroides galacturonicus	1	0%
Hungatella effluvii	1	0%
Lactobacillus antri	1	0%
marine gamma	1	0%
unclassified Solirubrobacterales	1	0%
Defluviitalea saccharophila	1	0%
Senegalimassilia anaerobia	1	0%
Candidatus Heliomonas	1	0%
Rhodobium orientis	1	0%
actinobacterium SCGC	1	0%
unclassified Nitrosovibrio	1	0%
Porphyromonas cangingivalis	1	0%
Acidicapsa ligni	1	0%
Clostridium hungatei	1	0%
Bifidobacterium catenulatum	1	0%
Synergistes jonesii	1	0%
Aquicella lusitana	1	0%
unclassified Ruminococcus	1	0%

unclassified Anaerovorax	1	0%
Bacteroides clarus	1	0%
unclassified Solirubrobacter	1	0%
Prevotella micans	1	0%
Lactobacillus tucceti	1	0%
Longilinea arvoryzae	1	0%
Breznakia pachnodae	1	0%
unclassified Pseudomonas	1	0%
Allisonella histaminiformans	1	0%
Bacillus pakistanensis	1	0%
Mogibacterium timidum	1	0%
Bacteroides acidifaciens	1	0%
Bifidobacterium coryneforme	1	0%
Streptomyces laculatispora	1	0%
Lactobacillus kalixensis	1	0%
Clostridium pasteurianum	1	0%
Bifidobacterium biavatii	1	0%
Bacteroides gallinarum	1	0%
Prevotella oulorum	1	0%
Oryzihumus leptocrescens	1	0%
Occallatibacter savannae	1	0%
Ruminococcus gauvreauii	1	0%
Bifidobacterium gallicum	1	0%
Aquihabitans daechungensis	1	0%
Acholeplasma hippikon	1	0%
Erysipelothrix rhusiopathiae	1	0%
unclassified Candidatus Solibacter	1	0%
Nocardioides islandensis	1	0%
Prevotella buccalis	1	0%
Hyphomicrobium hollandicum	1	0%
Paraclostridium bifermentans	1	0%
unclassified Spirochaetia	1	0%
Streptomyces scabrisporus	1	0%
Clostridium innocuum	1	0%
Dialister succinatiphilus	1	0%
Clostridium thermosuccinogenes	1	0%
Helicobacter rodentium	1	0%
Bacteroidales str.	1	0%
Porphyromonas pasteri	1	0%
unclassified Faecalibacterium	1	0%
Lactobacillus plantarum	1	0%
Selenomonas lacticifex	1	0%
unclassified Rubrobacter	1	0%
Ferruginibacter profundus	1	0%
Coprobacillus cateniformis	1	0%
Bacillus niabensis	1	0%
Roseburia cecicola	1	0%
Pseudolabrys taiwanensis	1	0%
Garciella nitratireducens	1	0%
Anaerocolumna aminovalerica	1	0%

Clostridium aurantibutyricum	1	0%
Thermoleophilum album	1	0%
unclassified Haloplasmataceae	1	0%
Clostridium taeniosporum	1	0%
Prevotella bryantii	1	0%
Collinsella massiliensis	1	0%
Slackia isoflavoniconvertens	1	0%
Cellulomonas hominis	1	0%
Bacillus pseudofirmus	1	0%
Rhodoferax ferrireducens	1	0%
Oceanibaculum pacificum	1	0%
Acidibacter ferrireducens	1	0%
Streptomyces ipomoeae	1	0%
Phycicoccus aerophilus	1	0%
Eubacterium pyruvativorans	1	0%
Pleomorphochaeta multiformis	1	0%
Actinomycetospora rishiriensis	1	0%
Clostridium viride	1	0%
unclassified Treponema	1	0%
Methyloligella halotolerans	1	0%
Streptococcus danieliae	1	0%
Bacteroides graminisolvens	1	0%
unclassified Oscillibacter	1	0%
Gorbachella massiliensis	1	0%
Prevotella nanceiensis	1	0%
Sporomusa malonica	1	0%
Clostridium celerecrescens	1	0%
Slackia exigua	1	0%
Clostridium neopropionicum	1	0%
Pirellula staleyi	1	0%
Caldilinea tarbellica	1	0%
Dehalogenimonas alkenigignens	1	0%
Methyloferula stellata	1	0%
unclassified Holophaga	1	0%
Hyphomicrobium facile	1	0%
Treponema bryantii	1	0%
Providencia rettgeri	1	0%
Coxiella endosymbiont	1	0%
Bacillus longiquaesitum	1	0%
Parabacteroides faecis	1	0%
Marmoricola pocheonesis	1	0%
Helicobacter equorum	1	0%
Defluviitalea raffinosedens	1	0%
Anaerolinea thermolimosa	1	0%
Paraburkholderia caledonica	1	0%
unclassified Geobacter	1	0%
Eubacterium limosum	1	0%
Lactobacillus casei	1	0%
Bacteroides faecis	1	0%
Pseudomonas putida	1	0%

unclassified Haliangium	1	0%
unclassified Acidobacterium	1	0%
Pseudomonas lurida	1	0%
Zhihengliuella flava	1	0%
Lactobacillus vaginalis	1	0%
Nitrospira japonica	1	0%
