# Supplementary material for: Influence of the Fermented Feed and Vaccination and Their Interaction on Parameters of Large White/Norwegian Landrace Piglets
Source: Animals (Basel). 2020 Jul 15;10(7):1201. doi: 10.3390/ani10071201 (PMC7401620; doi:10.3390/ani10071201)
Supplement: Supplementary file 1 [file animals-10-01201-s001.zip › Table S4 Species RFV group before experiment.pdf]

| Species RFV group before experiment | Number of reads | Relative abundance |
|---|---|---|
| Lactobacillus amylovorus | 6040 | 16.92% |
| Prevotella copri | 5674 | 15.9% |
| Collinsella aerofaciens | 1383 | 3.87% |
| Unclassified | 1340 | 3.75% |
| Selenomonas bovis | 913 | 2.56% |
| Faecalibacterium prausnitzii | 822 | 2.3% |
| Olsenella scatoligenes | 755 | 2.12% |
| Prevotella stercorea | 700 | 1.96% |
| Barnesiella intestinihominis | 610 | 1.71% |
| Prevotella oris | 515 | 1.44% |
| Oligosphaera ethanolica | 458 | 1.28% |
| Roseburia faecis | 444 | 1.24% |
| Denitrobacterium detoxificans | 442 | 1.24% |
| Oscillibacter ruminantium | 429 | 1.2% |
| Megasphaera elsdenii | 423 | 1.19% |
| unclassified Bacteroidales | 389 | 1.09% |
| Succinivibrio dextrinosolvens | 373 | 1.04% |
| Gemmiger formicilis | 351 | 0.98% |
| Alloprevotella rava | 349 | 0.98% |
| Flintibacter butyricus | 338 | 0.95% |
| Oscillospira guilliermondii | 329 | 0.92% |
| Bifidobacterium boum | 309 | 0.87% |
| Olivibacter sitiensis | 299 | 0.84% |
| Ruminococcus bromii | 295 | 0.83% |
| Enorma massiliensis | 268 | 0.75% |
| Desulfovibrio piger | 246 | 0.69% |
| Olsenella uli | 238 | 0.67% |
| Lactobacillus crispatus | 237 | 0.66% |
| Intestinimonas timonensis | 232 | 0.65% |
| unclassified Planctomycetales | 216 | 0.61% |
| Prevotella oralis | 211 | 0.59% |
| Enterorhabdus mucosicola | 208 | 0.58% |
| Eubacterium rectale | 207 | 0.58% |
| Campylobacter lanienae | 195 | 0.55% |
| Gracilibacter thermotolerans | 193 | 0.54% |
| Anaerovibrio lipolyticus | 193 | 0.54% |
| Escherichia coli | 171 | 0.48% |
| Blautia wexlerae | 169 | 0.47% |
| Phascolarctobacterium succinatutens | 157 | 0.44% |
| Bacteroidales oral | 152 | 0.43% |
| Sporobacter termitidis | 143 | 0.4% |
| Lactobacillus panis | 139 | 0.39% |
| Eubacterium cellulosolvens | 133 | 0.37% |
| Lactobacillus kitasatonis | 123 | 0.34% |
| Butyricicoccus pullicaecorum | 120 | 0.34% |
| Prevotella brevis | 120 | 0.34% |
| Lactobacillus reuteri | 120 | 0.34% |
| Intestinimonas butyriciproducens | 117 | 0.33% |
| Parabacteroides distasonis | 111 | 0.31% |

| | | |
|---|---|---|
| Holdemanella biformis | 104 | 0.29% |
| Prevotella dentalis | 99 | 0.28% |
| Bifidobacterium choerinum | 93 | 0.26% |
| Lactobacillus delbrueckii | 91 | 0.25% |
| Parabacteroides goldsteinii | 89 | 0.25% |
| Eubacterium coprostanoligenes | 88 | 0.25% |
| Prevotella paludivivens | 87 | 0.24% |
| Clostridium phoceensis | 84 | 0.24% |
| Fusicatenibacter saccharivorans | 84 | 0.24% |
| Prevotella conceptionensis | 82 | 0.23% |
| Lactobacillus jensenii | 74 | 0.21% |
| Paraprevotella clara | 74 | 0.21% |
| Bifidobacterium pseudolongum | 73 | 0.2% |
| Murimonas intestini | 73 | 0.2% |
| unclassified Tannerella | 69 | 0.19% |
| Lactobacillus pontis | 68 | 0.19% |
| Terrisporobacter glycolicus | 68 | 0.19% |
| Clostridium chartatabidum | 68 | 0.19% |
| Anaerobium acetethylicum | 67 | 0.19% |
| Caloramator fervidus | 66 | 0.18% |
| Ruminococcus bicirculans | 63 | 0.18% |
| Acetanaerobacterium elongatum | 62 | 0.17% |
| Acidaminobacter hydrogenoformans | 59 | 0.17% |
| Treponema bryantii | 59 | 0.17% |
| unclassified Prevotella | 59 | 0.17% |
| Olsenella profusa | 57 | 0.16% |
| Enterorhabdus caecimuris | 57 | 0.16% |
| Eubacterium eligens | 56 | 0.16% |
| Eubacterium ramulus | 55 | 0.15% |
| Intestinimonas massiliensis | 54 | 0.15% |
| Fournierella massiliensis | 54 | 0.15% |
| Blautia obeum | 53 | 0.15% |
| Catenibacterium mitsuokai | 52 | 0.15% |
| Clostridium cellulovorans | 51 | 0.14% |
| Lactobacillus frumenti | 51 | 0.14% |
| Bifidobacterium thermophilum | 50 | 0.14% |
| unclassified Barnesiella | 50 | 0.14% |
| Paludibacter propionicigenes | 50 | 0.14% |
| Bacteroides barnesiae | 50 | 0.14% |
| Eubacterium pyruvativorans | 49 | 0.14% |
| Ruminococcus faecis | 49 | 0.14% |
| Saccharofermentans acetigenes | 48 | 0.13% |
| Parvibacter caecicola | 46 | 0.13% |
| Bifidobacteriaceae genomosp. | 46 | 0.13% |
| Christensenella minuta | 45 | 0.13% |
| Intestinibacter bartlettii | 44 | 0.12% |
| Anaerovorax odorimutans | 44 | 0.12% |
| Candidatus Soleaferrea | 43 | 0.12% |
| Ruminiclostridium thermocellum | 43 | 0.12% |
| unclassified Rikenella | 43 | 0.12% |

| | | |
|---|---|---|
| Ruminococcus torques | 43 | 0.12% |
| Coprococcus comes | 43 | 0.12% |
| Collinsella intestinalis | 42 | 0.12% |
| Ruminococcus flavefaciens | 40 | 0.11% |
| Prevotella loescheii | 40 | 0.11% |
| Succiniclasticum ruminis | 39 | 0.11% |
| Lactobacillus helveticus | 39 | 0.11% |
| Prevotella genomosp. | 38 | 0.11% |
| Parabacteroides chinchillae | 38 | 0.11% |
| Acidaminococcus fermentans | 37 | 0.1% |
| Clostridium saccharolyticum | 37 | 0.1% |
| Anaeromassilibacillus senegalensis | 37 | 0.1% |
| Clostridium aldenense | 37 | 0.1% |
| Clostridium populeti | 36 | 0.1% |
| Sphaerochaeta coccoides | 34 | 0.1% |
| Mogibacterium diversum | 34 | 0.1% |
| Desulfovibrio fairfieldensis | 34 | 0.1% |
| Prevotella salivae | 31 | 0.09% |
| Bacteroides stercoris | 31 | 0.09% |
| Clostridium aminobutyricum | 31 | 0.09% |
| Candidatus Treponema | 31 | 0.09% |
| Clostridium xylanolyticum | 31 | 0.09% |
| Blautia massiliensis | 30 | 0.08% |
| unclassified Prevotellaceae | 30 | 0.08% |
| Blautia glucerasea | 30 | 0.08% |
| Holdemania filiformis | 29 | 0.08% |
| Clostridium viride | 29 | 0.08% |
| Anaerotaenia torta | 28 | 0.08% |
| Lactobacillus johnsonii | 28 | 0.08% |
| Lactobacillus acidophilus | 28 | 0.08% |
| Caloranaerobacter azorensis | 28 | 0.08% |
| unclassified Bacteroidia | 28 | 0.08% |
| Eubacterium hallii | 28 | 0.08% |
| Mitsuokella jalaludinii | 27 | 0.08% |
| Prevotella shahii | 27 | 0.08% |
| Ruminococcus lactaris | 27 | 0.08% |
| Eubacterium siraeum | 27 | 0.08% |
| Clostridium celatum | 26 | 0.07% |
| Sharpea azabuensis | 26 | 0.07% |
| Roseburia inulinivorans | 26 | 0.07% |
| Desulfotomaculum guttoideum | 26 | 0.07% |
| Coprococcus catus | 25 | 0.07% |
| unclassified Alloprevotella | 25 | 0.07% |
| Slackia exigua | 24 | 0.07% |
| Sutterella stercoricanis | 24 | 0.07% |
| Blautia schinkii | 23 | 0.06% |
| unclassified Erysipelotrichaceae | 23 | 0.06% |
| Geosporobacter ferrireducens | 23 | 0.06% |
| Blautia stercoris | 22 | 0.06% |
| unclassified Lachnospiraceae | 22 | 0.06% |

| | | |
|---|---|---|
| Ruminococcus callidus | 22 | 0.06% |
| Agathobacter ruminis | 22 | 0.06% |
| Collinsella massiliensis | 22 | 0.06% |
| Falcatimonas natans | 21 | 0.06% |
| Slackia piriformis | 21 | 0.06% |
| Clostridium asparagiforme | 21 | 0.06% |
| unclassified Olsenella | 21 | 0.06% |
| Butyrivibrio fibrisolvens | 21 | 0.06% |
| Eubacterium infirmum | 20 | 0.06% |
| Bifidobacterium breve | 20 | 0.06% |
| cyanobacterium enrichment | 20 | 0.06% |
| Adlercreutzia equolifaciens | 20 | 0.06% |
| Solobacterium moorei | 20 | 0.06% |
| Clostridium longisporum | 20 | 0.06% |
| Anaerobacterium chartisolvens | 19 | 0.05% |
| Oribacterium sinus | 19 | 0.05% |
| Hungatella hathewayi | 19 | 0.05% |
| Candidatus Heliomonas | 19 | 0.05% |
| Papillibacter cinnamivorans | 19 | 0.05% |
| Porphyromonas catoniae | 19 | 0.05% |
| Natranaerovirga pectinivora | 19 | 0.05% |
| Eubacteriaceae oral | 19 | 0.05% |
| Thermotalea metallivorans | 19 | 0.05% |
| Dorea longicatena | 19 | 0.05% |
| Pseudoflavonifractor capillosus | 18 | 0.05% |
| Olsenella umbonata | 18 | 0.05% |
| Ruminococcus albus | 18 | 0.05% |
| unclassified Deltaproteobacteria | 18 | 0.05% |
| Eubacterium ruminantium | 18 | 0.05% |
| unclassified Chlamydiia | 18 | 0.05% |
| Eubacterium desmolans | 18 | 0.05% |
| Paraeggerthella hongkongensis | 18 | 0.05% |
| Desulfotomaculum tongense | 17 | 0.05% |
| Ruthenibacterium lactatiformans | 17 | 0.05% |
| Clostridium colicanis | 17 | 0.05% |
| Turicibacter sanguinis | 17 | 0.05% |
| Barnesiella viscericola | 17 | 0.05% |
| Prevotella buccae | 17 | 0.05% |
| Faecalicoccus acidiformans | 17 | 0.05% |
| Clostridium tepidiprofundi | 17 | 0.05% |
| Clostridium polysaccharolyticum | 17 | 0.05% |
| Bacteroides salanitronis | 17 | 0.05% |
| unclassified Eubacterium | 17 | 0.05% |
| Ruminococcus gnavus | 16 | 0.04% |
| Bifidobacterium pullorum | 16 | 0.04% |
| Erysipelothrix inopinata | 16 | 0.04% |
| Blautia producta | 16 | 0.04% |
| Acetivibrio ethanolgignens | 16 | 0.04% |
| Oscillibacter valericigenes | 16 | 0.04% |
| Blautia faecis | 15 | 0.04% |

| | | |
|---|---|---|
| Peptococcus simiae | 15 | 0.04% |
| Catabacter hongkongensis | 15 | 0.04% |
| Fucophilus fucoidanolyticus | 15 | 0.04% |
| Garciella nitratireducens | 15 | 0.04% |
| Desulfotomaculum halophilum | 15 | 0.04% |
| Intestinimonas gabonensis | 15 | 0.04% |
| Alloprevotella tannerae | 15 | 0.04% |
| Candidatus Dorea | 14 | 0.04% |
| Herbinix luporum | 14 | 0.04% |
| Clostridium lactatifermentans | 14 | 0.04% |
| Lutispora thermophila | 14 | 0.04% |
| Treponema succinifaciens | 14 | 0.04% |
| Sphaerochaeta pleomorpha | 14 | 0.04% |
| unclassified Clostridiales | 14 | 0.04% |
| Marvinbryantia formatexigens | 13 | 0.04% |
| Clostridium leptum | 13 | 0.04% |
| Methanobrevibacter smithii | 13 | 0.04% |
| Eisenbergiella tayi | 13 | 0.04% |
| unclassified Clostridia | 13 | 0.04% |
| Cloacibacillus porcorum | 13 | 0.04% |
| Bacteroidales genomosp. | 12 | 0.03% |
| Clostridium cellobioparum | 12 | 0.03% |
| Prevotella maculosa | 12 | 0.03% |
| Bifidobacterium longum | 12 | 0.03% |
| Caminicella sporogenes | 12 | 0.03% |
| methanogenic archaeon | 12 | 0.03% |
| Vallitalea pronyensis | 12 | 0.03% |
| Dorea formicigenerans | 12 | 0.03% |
| Desulfotomaculum nigrificans | 11 | 0.03% |
| Collinsella stercoris | 11 | 0.03% |
| unclassified Ruminococcaceae | 11 | 0.03% |
| Pleomorphochaeta multiformis | 11 | 0.03% |
| Bacteroides uniformis | 11 | 0.03% |
| unclassified Porphyromonadaceae | 11 | 0.03% |
| Treponema porcinum | 10 | 0.03% |
| Kluyvera georgiana | 10 | 0.03% |
| unclassified Paludibacter | 10 | 0.03% |
| Roseburia hominis | 10 | 0.03% |
| Paludibacter jiangxiensis | 10 | 0.03% |
| Rarimicrobium hominis | 10 | 0.03% |
| Prevotella baroniae | 10 | 0.03% |
| Clostridium aerotolerans | 10 | 0.03% |
| Blautia luti | 10 | 0.03% |
| Hespellia porcina | 10 | 0.03% |
| Bacteroides caecigallinarum | 10 | 0.03% |
| unclassified Clostridium | 9 | 0.03% |
| Romboutsia sedimentorum | 9 | 0.03% |
| Anaerocolumna cellulosilytica | 9 | 0.03% |
| unclassified Turicibacter | 9 | 0.03% |
| unclassified Ruminococcus | 9 | 0.03% |

| | | |
|---|---|---|
| Lactobacillus secaliphilus | 9 | 0.03% |
| Clostridium sphenoides | 9 | 0.03% |
| Lactobacillus mucosae | 9 | 0.03% |
| Prevotella ruminicola | 9 | 0.03% |
| Clostridium oroticum | 9 | 0.03% |
| Selenomonas sputigena | 9 | 0.03% |
| Clostridium cellulolyticum | 8 | 0.02% |
| Robinsoniella peoriensis | 8 | 0.02% |
| Treponema zioleckii | 8 | 0.02% |
| Eubacterium oxidoreducens | 8 | 0.02% |
| Methylocystis rosea | 8 | 0.02% |
| Eubacterium rangiferina | 8 | 0.02% |
| Flavonifractor plautii | 8 | 0.02% |
| Clostridium symbiosum | 8 | 0.02% |
| Treponema brennaborense | 8 | 0.02% |
| Prevotella denticola | 8 | 0.02% |
| Slackia isoflavoniconvertens | 8 | 0.02% |
| unclassified Bacteroides | 7 | 0.02% |
| actinobacterium enrichment | 7 | 0.02% |
| Prevotella bivia | 7 | 0.02% |
| Bacteroides helcogenes | 7 | 0.02% |
| Clostridium putrefaciens | 7 | 0.02% |
| Clostridium bovipellis | 7 | 0.02% |
| Bacteroides faecis | 7 | 0.02% |
| Desulfovibrio desulfuricans | 7 | 0.02% |
| unclassified Bulleidia | 7 | 0.02% |
| unclassified Candidatus Glomeribacter | 7 | 0.02% |
| Mucispirillum schaedleri | 7 | 0.02% |
| Brassicibacter thermophilus | 7 | 0.02% |
| Fibrobacter intestinalis | 7 | 0.02% |
| Helicobacter rodentium | 7 | 0.02% |
| Clostridium papyrosolvens | 7 | 0.02% |
| Lachnospira pectinoschiza | 7 | 0.02% |
| Alkalibacter saccharofermentans | 7 | 0.02% |
| Clostridium neopropionicum | 6 | 0.02% |
| Lactobacillus amylolyticus | 6 | 0.02% |
| Bacteroides nordii | 6 | 0.02% |
| Mitsuokella multacida | 6 | 0.02% |
| Bifidobacterium kashiwanohense | 6 | 0.02% |
| Ethanoligenens harbinense | 6 | 0.02% |
| Escherichia albertii | 6 | 0.02% |
| Subdoligranulum variabile | 6 | 0.02% |
| unclassified Mollicutes | 6 | 0.02% |
| unclassified Oscillibacter | 6 | 0.02% |
| unclassified Bifidobacterium | 6 | 0.02% |
| Roseburia intestinalis | 6 | 0.02% |
| Clostridium methylpentosum | 6 | 0.02% |
| Clostridium celerecrescens | 6 | 0.02% |
| Bifidobacterium reuteri | 6 | 0.02% |
| Bacteroides intestinalis | 6 | 0.02% |

| | | |
|---|---|---|
| Clostridium fusiformis | 6 | 0.02% |
| Pseudomonas fluorescens | 6 | 0.02% |
| Anaerostipes hadrus | 6 | 0.02% |
| Bacteroides caccae | 6 | 0.02% |
| Coprococcus eutactus | 6 | 0.02% |
| Butyricimonas virosa | 6 | 0.02% |
| Thermanaerovibrio velox | 5 | 0.01% |
| Clostridium clostridioforme | 5 | 0.01% |
| Clostridium quinii | 5 | 0.01% |
| Parabacteroides merdae | 5 | 0.01% |
| unclassified Sporobacter | 5 | 0.01% |
| Oxobacter pfennigii | 5 | 0.01% |
| unclassified Oscillospira | 5 | 0.01% |
| Eubacterium sulci | 5 | 0.01% |
| unclassified Victivallaceae | 5 | 0.01% |
| unclassified Dysgonomonas | 5 | 0.01% |
| alpha proteobacterium | 5 | 0.01% |
| Asaccharospora irregularis | 5 | 0.01% |
| Faecalitalea cylindroides | 5 | 0.01% |
| Aeriscardovia aeriphila | 5 | 0.01% |
| Anaeroplasma abactoclasticum | 5 | 0.01% |
| Parasporobacterium paucivorans | 5 | 0.01% |
| Clostridium clariflavum | 5 | 0.01% |
| Parasutterella secunda | 5 | 0.01% |
| Moorella humiferrea | 5 | 0.01% |
| Campylobacter hyointestinalis | 5 | 0.01% |
| Lactobacillus gallinarum | 5 | 0.01% |
| Prevotella bryantii | 5 | 0.01% |
| Eubacterium contortum | 5 | 0.01% |
| Propionispira arcuata | 5 | 0.01% |
| Kosakonia sacchari | 5 | 0.01% |
| Selenomonas ruminantium | 5 | 0.01% |
| unclassified Anaerovibrio | 4 | 0.01% |
| Prevotella dentasini | 4 | 0.01% |
| Megasphaera paucivorans | 4 | 0.01% |
| Eggerthella lenta | 4 | 0.01% |
| Clostridium indolis | 4 | 0.01% |
| unclassified Sphingobacterium | 4 | 0.01% |
| Peptococcus niger | 4 | 0.01% |
| unclassified Succinivibrio | 4 | 0.01% |
| Megasphaera hominis | 4 | 0.01% |
| Bacteroides coprocola | 4 | 0.01% |
| Collinsella tanakaei | 4 | 0.01% |
| Senegalimassilia anaerobia | 4 | 0.01% |
| Acetivibrio cellulolyticus | 4 | 0.01% |
| Lactobacillus coleohominis | 4 | 0.01% |
| Clostridium disporicum | 4 | 0.01% |
| Prevotella amnii | 4 | 0.01% |
| Fusobacterium mortiferum | 4 | 0.01% |
| Bacteroides heparinolyticus | 4 | 0.01% |

| | | |
|---|---|---|
| unclassified Enterococcus | 4 | 0.01% |
| Porphyromonas cangingivalis | 4 | 0.01% |
| Bacteroides timonensis | 4 | 0.01% |
| Desulfovibrio intestinalis | 4 | 0.01% |
| Treponema berlinense | 4 | 0.01% |
| Clostridium piliforme | 4 | 0.01% |
| unclassified Lactobacillus | 4 | 0.01% |
| unclassified Collinsella | 4 | 0.01% |
| Prevotella fusca | 4 | 0.01% |
| Clostridium hungatei | 4 | 0.01% |
| Clostridium fimetarium | 4 | 0.01% |
| Anaerofilum pentosovorans | 4 | 0.01% |
| Erysipelothrix rhusiopathiae | 4 | 0.01% |
| Caldicoprobacter algeriensis | 3 | 0.01% |
| Lactobacillus gasseri | 3 | 0.01% |
| Clostridium sartagoforme | 3 | 0.01% |
| Propionispira paucivorans | 3 | 0.01% |
| unclassified Petrimonas | 3 | 0.01% |
| Porphyromonas pogonae | 3 | 0.01% |
| Bacteroides plebeius | 3 | 0.01% |
| Clostridium islandicum | 3 | 0.01% |
| Parabacteroides johnsonii | 3 | 0.01% |
| Bacteroides salyersiae | 3 | 0.01% |
| Elbe River | 3 | 0.01% |
| Bacteroides oleiciplenus | 3 | 0.01% |
| Bacteroides clarus | 3 | 0.01% |
| Clostridium tarantellae | 3 | 0.01% |
| Pyramidobacter piscolens | 3 | 0.01% |
| Gorbachella massiliensis | 3 | 0.01% |
| Anaerotruncus colihominis | 3 | 0.01% |
| Bifidobacterium animalis | 3 | 0.01% |
| unclassified Spirochaetia | 3 | 0.01% |
| Lactobacillus hamsteri | 3 | 0.01% |
| Lactobacillus agilis | 3 | 0.01% |
| Caproiciproducens galactitolivorans | 3 | 0.01% |
| Eubacterium plexicaudatum | 3 | 0.01% |
| Helicobacter canadensis | 3 | 0.01% |
| Macellibacteroides fermentans | 3 | 0.01% |
| Kiloniella spongiae | 3 | 0.01% |
| Akkermansia muciniphila | 3 | 0.01% |
| Candidatus Izimaplasma | 3 | 0.01% |
| Eubacterium minutum | 3 | 0.01% |
| Dehalobacterium formicoaceticum | 3 | 0.01% |
| Anaerostipes rhamnosivorans | 3 | 0.01% |
| Syntrophococcus sucromutans | 3 | 0.01% |
| Coriobacterium glomerans | 3 | 0.01% |
| Prevotella timonensis | 3 | 0.01% |
| Salmonella enterica | 3 | 0.01% |
| Acetatifactor muris | 3 | 0.01% |
| Bacteroides caecicola | 3 | 0.01% |

| | | |
|---|---|---|
| Clostridium straminisolvens | 3 | 0.01% |
| Acidaminococcus intestini | 3 | 0.01% |
| Cellulosibacter alkalithermophilus | 3 | 0.01% |
| Lachnobacterium bovis | 3 | 0.01% |
| Stomatobaculum longum | 3 | 0.01% |
| gamma proteobacterium | 3 | 0.01% |
| Elusimicrobium minutum | 3 | 0.01% |
| Bifidobacterium thermacidophilum | 3 | 0.01% |
| Mobilitalea sibirica | 3 | 0.01% |
| low G+C | 3 | 0.01% |
| Clostridium elmenteitii | 3 | 0.01% |
| Hallella seregens | 2 | 0.01% |
| unclassified Betaproteobacteria | 2 | 0.01% |
| unclassified Christensenella | 2 | 0.01% |
| Campylobacter coli | 2 | 0.01% |
| Helicobacter equorum | 2 | 0.01% |
| Cellulosilyticum ruminicola | 2 | 0.01% |
| unclassified Syntrophococcus | 2 | 0.01% |
| Bacteroides fragilis | 2 | 0.01% |
| unclassified Veillonellaceae | 2 | 0.01% |
| Eisenbergiella massiliensis | 2 | 0.01% |
| Parapedobacter luteus | 2 | 0.01% |
| unclassified Peptostreptococcaceae | 2 | 0.01% |
| Clostridium paraputrificum | 2 | 0.01% |
| Clostridium amygdalinum | 2 | 0.01% |
| Clostridium caenicola | 2 | 0.01% |
| Clostridium scindens | 2 | 0.01% |
| Faecalicoccus pleomorphus | 2 | 0.01% |
| Clostridium carnis | 2 | 0.01% |
| Victivallis vadensis | 2 | 0.01% |
| Herbinix hemicellulosilytica | 2 | 0.01% |
| unclassified Coriobacteriales | 2 | 0.01% |
| Anaerobiospirillum succiniciproducens | 2 | 0.01% |
| Campylobacter cf. | 2 | 0.01% |
| Verticillium dahliae | 2 | 0.01% |
| Bifidobacterium saeculare | 2 | 0.01% |
| Thermoflavimicrobium dichotomicum | 2 | 0.01% |
| unclassified Clostridiaceae | 2 | 0.01% |
| Alistipes finegoldii | 2 | 0.01% |
| Fusobacterium perfoetens | 2 | 0.01% |
| Ruminococcus champanellensis | 2 | 0.01% |
| Bacteroides acidifaciens | 2 | 0.01% |
| Lactobacillus salivarius | 2 | 0.01% |
| Clostridium botulinum | 2 | 0.01% |
| Lactobacillus gastricus | 2 | 0.01% |
| Asteroleplasma anaerobium | 2 | 0.01% |
| Mageeibacillus indolicus | 2 | 0.01% |
| Citrobacter freundii | 2 | 0.01% |
| Prevotella buccalis | 2 | 0.01% |
| Clostridium oceanicum | 2 | 0.01% |

| | | |
|---|---|---|
| Eubacterium xylanophilum | 2 | 0.01% |
| Fodinicurvata fenggangensis | 2 | 0.01% |
| Actinomyces howellii | 2 | 0.01% |
| Cytophaga xylanolytica | 2 | 0.01% |
| Clostridium butyricum | 2 | 0.01% |
| Lachnoanaerobaculum umeaense | 2 | 0.01% |
| Bacteroides pyogenes | 2 | 0.01% |
| marine alpha | 2 | 0.01% |
| Clostridium sulfidigenes | 2 | 0.01% |
| Photorhabdus luminescens | 2 | 0.01% |
| unclassified Actinomycetales | 2 | 0.01% |
| Phycicola gilvus | 2 | 0.01% |
| Clostridium hylemonae | 2 | 0.01% |
| Lactobacillus vaginalis | 2 | 0.01% |
| Clostridium lavalense | 2 | 0.01% |
| Wukongibacter baidiensis | 2 | 0.01% |
| Lactobacillus kefiranofaciens | 2 | 0.01% |
| unclassified Erysipelothrix | 2 | 0.01% |
| Bacteroides stercorirosoris | 2 | 0.01% |
| Ruminobacter amylophilus | 2 | 0.01% |
| Tindallia magadiensis | 2 | 0.01% |
| Clostridium hiranonis | 2 | 0.01% |
| Anaerosporobacter mobilis | 2 | 0.01% |
| Lactobacillus hominis | 2 | 0.01% |
| Tepidamorphus gemmatus | 1 | 0% |
| Ruminococcus gauvreauii | 1 | 0% |
| Helicobacter rappini | 1 | 0% |
| Abyssivirga alkaniphila | 1 | 0% |
| Streptomyces clavuligerus | 1 | 0% |
| Bacillus nealsonii | 1 | 0% |
| Desulfotomaculum varum | 1 | 0% |
| Prevotella oulorum | 1 | 0% |
| Shigella dysenteriae | 1 | 0% |
| Lactobacillus perolens | 1 | 0% |
| Clostridium aminophilum | 1 | 0% |
| Prevotella albensis | 1 | 0% |
| Lachnospiraceae oral | 1 | 0% |
| Gordonibacter urolithinfaciens | 1 | 0% |
| Rhodobium orientis | 1 | 0% |
| Desulfotomaculum geothermicum | 1 | 0% |
| unclassified Intestinimonas | 1 | 0% |
| Clostridium thermosuccinogenes | 1 | 0% |
| Odoribacter laneus | 1 | 0% |
| Actinocorallia cavernae | 1 | 0% |
| Atopobium vaginae | 1 | 0% |
| Bifidobacterium adolescentis | 1 | 0% |
| unclassified Bacillus | 1 | 0% |
| Desulfovibrio simplex | 1 | 0% |
| unclassified Desulfovirga | 1 | 0% |
| Anaeroplasma bactoclasticum | 1 | 0% |

| | | |
|---|---|---|
| Blastococcus jejuensis | 1 | 0% |
| unclassified Bacteriovorax | 1 | 0% |
| unclassified Frankia | 1 | 0% |
| Clostridium colinum | 1 | 0% |
| Brevundimonas aurantiaca | 1 | 0% |
| unclassified Marinilabilia | 1 | 0% |
| Methylopila oligotropha | 1 | 0% |
| Bifidobacterium stellenboschense | 1 | 0% |
| Clostridium sporosphaeroides | 1 | 0% |
| Caloramator australicus | 1 | 0% |
| Clostridium stercorarium | 1 | 0% |
| Pedosphaera parvula | 1 | 0% |
| Lactobacillus oris | 1 | 0% |
| Azospirillum lipoferum | 1 | 0% |
| Geodermatophilus africanus | 1 | 0% |
| Lactobacillus paucivorans | 1 | 0% |
| Lactobacillus taiwanensis | 1 | 0% |
| Methylocella palustris | 1 | 0% |
| unclassified Akkermansia | 1 | 0% |
| Prevotella saccharolytica | 1 | 0% |
| unclassified Bacteroidaceae | 1 | 0% |
| Eubacterium tenue | 1 | 0% |
| Clostridium chauvoei | 1 | 0% |
| Bacteroides galacturonicus | 1 | 0% |
| Helicobacter ganmani | 1 | 0% |
| Mycobacterium poriferae | 1 | 0% |
| Enorma timonensis | 1 | 0% |
| unclassified Verrucomicrobiales | 1 | 0% |
| Dysgonomonas gadei | 1 | 0% |
| Clostridium vincentii | 1 | 0% |
| unclassified Wautersiella | 1 | 0% |
| Bacteroides pectinophilus | 1 | 0% |
| unclassified Spirochaeta | 1 | 0% |
| Thermovenabulum ferriorganovorum | 1 | 0% |
| unclassified Gordonibacter | 1 | 0% |
| unclassified Cryptanaerobacter | 1 | 0% |
| Aminicella lysinilytica | 1 | 0% |
| Gram-positive thermophile | 1 | 0% |
| Prevotella nigrescens | 1 | 0% |
| Bifidobacterium subtile | 1 | 0% |
| Clostridium josui | 1 | 0% |
| Actinomyces hyovaginalis | 1 | 0% |
| Clostridium acetobutylicum | 1 | 0% |
| Prevotella micans | 1 | 0% |
| Bifidobacterium saguini | 1 | 0% |
| Desulfomonas oviles | 1 | 0% |
| Breznakia pachnodae | 1 | 0% |
| Geosporobacter subterraneus | 1 | 0% |
| unclassified Blastococcus | 1 | 0% |
| Mangroviflexus xiamenensis | 1 | 0% |

| | | |
|---|---|---|
| Tannerella forsythia | 1 | 0% |
| unclassified Sporichthya | 1 | 0% |
| Pseudomonas savastanoi | 1 | 0% |
| Butyricimonas paravirosa | 1 | 0% |
| Lactobacillus rossiae | 1 | 0% |
| Prevotella multisaccharivorax | 1 | 0% |
| Eubacterium dolichum | 1 | 0% |
| Anaerorhabdus furcosa | 1 | 0% |
| Bilophila wadsworthia | 1 | 0% |
| unclassified Haloplasmataceae | 1 | 0% |
| Clostridium baratii | 1 | 0% |
| Sanguibacteroides justesenii | 1 | 0% |
| Desulfotomaculum alcoholivorax | 1 | 0% |
| Lachnoanaerobaculum cf. | 1 | 0% |
| Microbacter margulisiae | 1 | 0% |
| unclassified Candidatus Solibacter | 1 | 0% |
| Eubacterium budayi | 1 | 0% |
| Pseudoxanthobacter soli | 1 | 0% |
| Prevotella scopos | 1 | 0% |
| Methylosinus sporium | 1 | 0% |
| Holdemania massiliensis | 1 | 0% |
| Actinobacillus minor | 1 | 0% |
| unclassified Megasphaera | 1 | 0% |
| Pseudobutyrivibrio ruminis | 1 | 0% |
| Bacteroides rodentium | 1 | 0% |
| Fusobacterium russii | 1 | 0% |
| Gabonibacter massiliensis | 1 | 0% |
| Rhodanobacter spathiphylli | 1 | 0% |
| unclassified Actinobacteria | 1 | 0% |
| Herbivorax saccincola | 1 | 0% |
| Pseudoramibacter alactolyticus | 1 | 0% |
| Porphyromonas pasteri | 1 | 0% |
| Lactobacillus plantarum | 1 | 0% |
| unclassified Faecalibacterium | 1 | 0% |
| Labilibacter marinus | 1 | 0% |
| Desulfovibrio multispirans | 1 | 0% |
| Roseburia cecicola | 1 | 0% |
| Blautia hydrogenotrophica | 1 | 0% |
| Anaerocolumna aminovalerica | 1 | 0% |
| unclassified Steroidobacter | 1 | 0% |
| Clostridium sufflavum | 1 | 0% |
| Brassicibacter mesophilus | 1 | 0% |
| unclassified Methanobrevibacter | 1 | 0% |
| Pedobacter lotistagni | 1 | 0% |
| Pontibacter humi | 1 | 0% |
| Candidatus Mycoplasma | 1 | 0% |
| Paeniclostridium sordellii | 1 | 0% |
| Paeniclostridium ghonii | 1 | 0% |
| Desulfotomaculum profundi | 1 | 0% |
| Anaerocolumna jejuensis | 1 | 0% |

| | | |
|---|---|---|
| Treponema parvum | 1 | 0% |
| Clostridium neonatale | 1 | 0% |
| Clostridium tyrobutyricum | 1 | 0% |
| Clostridium amylolyticum | 1 | 0% |
| Campylobacter jejuni | 1 | 0% |
| Actinobacillus porcitonsillarum | 1 | 0% |
| Lachnoanaerobaculum orale | 1 | 0% |
| Sellimonas intestinalis | 1 | 0% |
| beta proteobacterium | 1 | 0% |
| Brevundimonas kwangchunensis | 1 | 0% |
| Prevotella enoeca | 1 | 0% |
| Corynebacterium provencense | 1 | 0% |
| Anaerocolumna xylanovorans | 1 | 0% |
| unclassified Opitutus | 1 | 0% |
| Niveispirillum irakense | 1 | 0% |
| Anaerofustis stercorihominis | 1 | 0% |
| Bacteroides paurosaccharolyticus | 1 | 0% |
| Hydrogenoanaerobacterium saccharovorans | 1 | 0% |
| Clostridium purinilyticum | 1 | 0% |
| Desulfurispora thermophila | 1 | 0% |
| Geoalkalibacter subterraneus | 1 | 0% |
| Bifidobacterium callitrichos | 1 | 0% |
| Asaccharobacter celatus | 1 | 0% |
| Bacteroides vulgatus | 1 | 0% |
| Edaphobacter dinghuensis | 1 | 0% |
| metal-contaminated soil | 1 | 0% |
| Oxalobacter formigenes | 1 | 0% |
| Hymenobacter deserti | 1 | 0% |
| unclassified Nocardioides | 1 | 0% |
| Butyrivibrio crossotus | 1 | 0% |
| Clostridium cellulosi | 1 | 0% |
| Bacteroides cellulosilyticus | 1 | 0% |
| unclassified Desulfovibrio | 1 | 0% |
| unclassified Cytophaga | 1 | 0% |
| Thiohalobacter thiocyanaticus | 1 | 0% |
| Parabacteroides chartae | 1 | 0% |
| Caldicoprobacter faecalis | 1 | 0% |
| Parabacteroides gordonii | 1 | 0% |
| Bariatricus massiliensis | 1 | 0% |
| unclassified Saccharofermentans | 1 | 0% |
| unclassified Coriobacteriaceae | 1 | 0% |
| Lactobacillus casei | 1 | 0% |
| Serpentinicella alkaliphila | 1 | 0% |
| Natranaerobius thermophilus | 1 | 0% |
| unclassified Acidobacterium | 1 | 0% |
| Tepidimicrobium xylanilyticum | 1 | 0% |
| Lachnoanaerobaculum saburreum | 1 | 0% |
