# Supplementary material for: Influence of the Fermented Feed and Vaccination and Their Interaction on Parameters of Large White/Norwegian Landrace Piglets
Source: Animals (Basel). 2020 Jul 15;10(7):1201. doi: 10.3390/ani10071201 (PMC7401620; doi:10.3390/ani10071201)
Supplement: Supplementary file 1 [file animals-10-01201-s001.zip › Table S5 Species SnonV group after experiment.pdf]

Species SnonV group after experiment	Number of reads	Relative abundance
Prevotella copri	14483	41.58%
Clostridium cellulovorans	2471	7.09%
Lactobacillus amylovorus	1741	5%
Terrisporobacter glycolicus	1403	4.03%
Prevotella stercorea	1126	3.23%
Faecalibacterium prausnitzii	972	2.79%
Anaerovibrio lipolyticus	970	2.78%
Gemmiger formicilis	787	2.26%
Unclassified	748	2.15%
Prevotella oralis	637	1.83%
Prevotella oris	586	1.68%
Alloprevotella rava	470	1.35%
Clostridium celatum	435	1.25%
Prevotella brevis	368	1.06%
Eubacterium rectale	308	0.88%
Roseburia faecis	303	0.87%
Megasphaera elsdenii	272	0.78%
Butyricicoccus pullicaecorum	231	0.66%
Prevotella histicola	225	0.65%
Barnesiella intestinihominis	215	0.62%
Lactobacillus reuteri	172	0.49%
Blautia wexlerae	155	0.44%
Phascolarctobacterium succinatutens	148	0.42%
Sporobacter termitidis	135	0.39%
Intestinibacter bartlettii	135	0.39%
Prevotella dentalis	131	0.38%
Lactobacillus pontis	129	0.37%
Paraprevotella clara	118	0.34%
Fusicatenibacter saccharivorans	111	0.32%
Romboutsia sedimentorum	109	0.31%
Eubacterium ramulus	104	0.3%
Eubacterium coprostanoligenes	101	0.29%
Clostridium quinii	92	0.26%
Lactobacillus crispatus	86	0.25%
Lactobacillus panis	85	0.24%
Catenibacterium mitsuokai	84	0.24%
Oscillospira guilliermondii	81	0.23%
Bacteroidales oral	78	0.22%
Blautia obeum	67	0.19%
Lactobacillus kitasatonis	67	0.19%
Eubacterium hallii	65	0.19%
Ruminococcus faecis	63	0.18%
unclassified Prevotella	60	0.17%
cyanobacterium enrichment	58	0.17%
Dialister succinatiphilus	56	0.16%
Clostridium amylolyticum	52	0.15%
Campylobacter lanienae	48	0.14%
Ruminococcus bicirculans	48	0.14%
unclassified Bacteroidales	47	0.13%

Fournierella massiliensis	46	0.13%
Solobacterium moorei	46	0.13%
Lactobacillus johnsonii	46	0.13%
Murimonas intestini	44	0.13%
Ruminiclostridium thermocellum	44	0.13%
Flintibacter butyricus	43	0.12%
Prevotella salivae	43	0.12%
Roseburia inulinivorans	43	0.12%
Lactobacillus jensenii	42	0.12%
unclassified Turicibacter	42	0.12%
Erysipelothrix inopinata	41	0.12%
Butyrivibrio fibrisolvens	40	0.11%
Clostridium disporicum	39	0.11%
unclassified Clostridium	39	0.11%
Prevotella genomosp.	38	0.11%
Holdemanella biformis	38	0.11%
Oscillibacter ruminantium	38	0.11%
Turicibacter sanguinis	36	0.1%
Prevotella ruminicola	36	0.1%
Asaccharospora irregularis	35	0.1%
Ruminococcus flavefaciens	34	0.1%
Clostridium sartagoforme	34	0.1%
Ruminococcus torques	34	0.1%
Coprococcus comes	32	0.09%
Eubacterium eligens	32	0.09%
Prevotella maculosa	31	0.09%
Sutterella stercoricanis	29	0.08%
Dorea formicigenerans	29	0.08%
Mitsuokella jalaludinii	29	0.08%
Lactobacillus delbrueckii	28	0.08%
Eubacterium desmolans	28	0.08%
unclassified Tannerella	28	0.08%
unclassified Barnesiella	27	0.08%
Clostridium bovipellis	27	0.08%
Eubacterium ruminantium	27	0.08%
Blautia faecis	26	0.07%
Blautia luti	26	0.07%
Dorea longicatena	25	0.07%
Collinsella aerofaciens	25	0.07%
Clostridium aldenense	25	0.07%
Hungatella hathewayi	25	0.07%
Lachnospira pectinoschiza	24	0.07%
Parabacteroides distasonis	24	0.07%
Blautia massiliensis	23	0.07%
Anaerotaenia torta	23	0.07%
Clostridium leptum	22	0.06%
Blautia glucerasea	22	0.06%
Clostridium saccharolyticum	21	0.06%
Coprococcus catus	21	0.06%
Ruminococcus bromii	21	0.06%

Coprococcus eutactus	21	0.06%
Clostridium cadaveris	20	0.06%
unclassified Lachnospiraceae	20	0.06%
Holdemania filiformis	20	0.06%
Selenomonas ruminantium	20	0.06%
Lactobacillus acidophilus	20	0.06%
Acetivibrio ethanolgignens	19	0.05%
Blautia stercoris	19	0.05%
Candidatus Soleaferrea	19	0.05%
Prevotella denticola	18	0.05%
Clostridium tertium	18	0.05%
Clostridium hveragerdense	18	0.05%
Oribacterium sinus	18	0.05%
Brassicibacter thermophilus	18	0.05%
Lactobacillus helveticus	17	0.05%
Clostridium chartatabidum	17	0.05%
Anaerostipes hadrus	17	0.05%
Anaerobacterium chartisolvens	17	0.05%
Falcatimonas natans	16	0.05%
Roseburia hominis	16	0.05%
Succinivibrio dextrinosolvens	16	0.05%
Roseburia intestinalis	15	0.04%
Agathobacter ruminis	15	0.04%
Clostridium chauvoei	15	0.04%
Clostridium polysaccharolyticum	15	0.04%
Propionispira arcuata	15	0.04%
Intestinimonas butyriciproducens	15	0.04%
Denitrobacterium detoxificans	14	0.04%
Candidatus Dorea	14	0.04%
Eubacterium xylanophilum	14	0.04%
Gracilibacter thermotolerans	14	0.04%
Ruminococcus callidus	13	0.04%
unclassified Erysipelotrichaceae	13	0.04%
Peptococcus simiae	13	0.04%
unclassified Ruminococcaceae	13	0.04%
unclassified Clostridiales	12	0.03%
Prevotella loescheii	12	0.03%
Bifidobacteriaceae genomosp.	12	0.03%
Elbe River	12	0.03%
Clostridium populeti	12	0.03%
Prevotella conceptionensis	12	0.03%
Clostridium aurantibutyricum	12	0.03%
unclassified Prevotellaceae	12	0.03%
unclassified Anaerovibrio	12	0.03%
Bacteroides intestinalis	11	0.03%
Anaerovorax odorimutans	11	0.03%
Olsenella scatoligenes	11	0.03%
Candidatus Treponema	11	0.03%
Lactobacillus frumenti	11	0.03%
Clostridium xylanolyticum	11	0.03%

Clostridium methylpentosum	11	0.03%
Eisenbergiella tayi	10	0.03%
Ruthenibacterium lactatiformans	10	0.03%
Clostridium cellulolyticum	10	0.03%
Marvinbryantia formatexigens	10	0.03%
Bifidobacterium pseudolongum	10	0.03%
Clostridium lavalense	10	0.03%
Prevotella paludivivens	9	0.03%
Prevotella buccae	9	0.03%
Gorbachella massiliensis	9	0.03%
Vallitalea pronyensis	9	0.03%
Methylocystis rosea	9	0.03%
Acidaminococcus fermentans	9	0.03%
Clostridium cellobioparum	9	0.03%
Herbinix luporum	9	0.03%
Blautia producta	9	0.03%
Intestinimonas timonensis	9	0.03%
unclassified Bacteroides	9	0.03%
Prevotella dentasini	9	0.03%
Enorma massiliensis	9	0.03%
Anaerobium acetethylicum	9	0.03%
Selenomonas bovis	9	0.03%
Parabacteroides chinchillae	8	0.02%
Natranaerovirga pectinivora	8	0.02%
Mogibacterium diversum	8	0.02%
Eubacterium oxidoreducens	8	0.02%
Prevotella bivia	8	0.02%
Mobilitalea sibirica	8	0.02%
Eubacterium tenue	8	0.02%
unclassified Planctomycetales	8	0.02%
Clostridium clostridioforme	8	0.02%
Acetivibrio cellulolyticus	8	0.02%
Intestinimonas massiliensis	8	0.02%
unclassified Mollicutes	8	0.02%
Treponema porcinum	7	0.02%
Bacteroidales genomosp.	7	0.02%
Clostridium fimetarium	7	0.02%
Eubacteriaceae oral	7	0.02%
Faecalitalea cylindroides	7	0.02%
Alloprevotella tannerae	7	0.02%
unclassified Bulleidia	7	0.02%
Subdoligranulum variabile	7	0.02%
Clostridium tarantellae	7	0.02%
Eubacterium rangiferina	7	0.02%
unclassified Candidatus Glomeribacter	6	0.02%
Porphyromonas catoniae	6	0.02%
Barnesiella viscericola	6	0.02%
Paeniclostridium ghonii	6	0.02%
Clostridium sphenoides	6	0.02%
Parasporobacterium paucivorans	6	0.02%

Selenomonas sputigena	6	0.02%
Hallella seregens	6	0.02%
Abyssivirga alkaniphila	6	0.02%
Clostridium butyricum	6	0.02%
Faecalicoccus acidiformans	6	0.02%
unclassified Acetivibrio	6	0.02%
Clostridium oroticum	6	0.02%
Clostridium symbiosum	6	0.02%
Terrisporobacter mayombei	6	0.02%
Clostridium phoceensis	6	0.02%
Lactobacillus fermentum	6	0.02%
Clostridium papyrosolvens	6	0.02%
Lactobacillus hamsteri	6	0.02%
Caloramator quimbayensis	6	0.02%
Olsenella profusa	5	0.01%
Lutispora thermophila	5	0.01%
Propionispira paucivorans	5	0.01%
Olsenella umbonata	5	0.01%
unclassified Eubacterium	5	0.01%
Bacteroides stercoris	5	0.01%
Clostridium hiranonis	5	0.01%
Lachnospira multipara	5	0.01%
Prevotella baroniae	5	0.01%
Bacteroides timonensis	5	0.01%
Anaerostipes butyraticus	4	0.01%
Clostridium botulinum	4	0.01%
Ruminococcus albus	4	0.01%
unclassified Clostridia	4	0.01%
Eubacterium cellulosolvens	4	0.01%
unclassified Bacteroidaceae	4	0.01%
Ruminococcus lactaris	4	0.01%
Lactobacillus rogosae	4	0.01%
Desulfovibrio piger	4	0.01%
Enterorhabdus mucosicola	4	0.01%
unclassified Alloprevotella	4	0.01%
Ruminococcus gnavus	4	0.01%
Flavonifractor plautii	4	0.01%
Prevotella enoeca	4	0.01%
Campylobacter hyointestinalis	4	0.01%
Anaerocolumna xylanovorans	4	0.01%
Olsenella uli	4	0.01%
Bacteroides zoogleoformans	4	0.01%
Clostridium intestinale	4	0.01%
Bacteroides clarus	4	0.01%
Mitsuokella multacida	4	0.01%
Clostridium fusiformis	4	0.01%
Corynebacterium provencense	3	0.01%
Clostridium asparagiforme	3	0.01%
Asteroleplasma anaerobium	3	0.01%
Clostridium celerecrescens	3	0.01%

Lactobacillus secaliphilus	3	0.01%
Desulfotomaculum guttoideum	3	0.01%
unclassified Clostridiaceae	3	0.01%
Clostridium hylemonae	3	0.01%
Ruminococcus gauvreauii	3	0.01%
Robinsoniella peoriensis	3	0.01%
Parabacteroides merdae	3	0.01%
Desulfovibrio fairfieldensis	3	0.01%
Helicobacter canadensis	3	0.01%
unclassified Ruminococcus	3	0.01%
Bacteroides cellulosilyticus	3	0.01%
Anaerosporobacter mobilis	3	0.01%
Anaerocolumna cellulosilytica	3	0.01%
unclassified Paludibacter	3	0.01%
Clostridium clariflavum	3	0.01%
Clostridium straminisolvens	3	0.01%
Clostridium indolis	3	0.01%
Hespellia porcina	3	0.01%
Prevotella shahii	3	0.01%
Coprobacillus cateniformis	3	0.01%
Bacteroides caecigallinarum	3	0.01%
unclassified Roseburia	3	0.01%
Prevotella bryantii	3	0.01%
Mycoplasma sualvi	3	0.01%
Prevotella scopos	3	0.01%
Paeniclostridium sordellii	3	0.01%
Prevotella albensis	3	0.01%
unclassified Oscillibacter	3	0.01%
Lactobacillus amylolyticus	3	0.01%
Bifidobacterium choerinum	3	0.01%
Clostridium baratii	3	0.01%
Ruminococcus champanellensis	3	0.01%
Butyrivibrio crossotus	3	0.01%
Eubacterium dolichum	3	0.01%
Erysipelothrix rhusiopathiae	3	0.01%
Lactobacillus oris	3	0.01%
Sphaerochaeta coccoides	3	0.01%
Bacteroides heparinolyticus	3	0.01%
Prevotella melaninogenica	2	0.01%
Bacteroides caecicola	2	0.01%
unclassified Solobacterium	2	0.01%
Hungatella effluvii	2	0.01%
Bacteroides salanitronis	2	0.01%
Blautia schinkii	2	0.01%
Sutterella wadsworthensis	2	0.01%
Parasutterella secunda	2	0.01%
Anaeromassilibacillus senegalensis	2	0.01%
Collinsella intestinalis	2	0.01%
Clostridium moniliforme	2	0.01%
Lactobacillus agilis	2	0.01%

Allisonella histaminiformans	2	0.01%
unclassified Wautersiella	2	0.01%
Clostridium aerotolerans	2	0.01%
Sphaerochaeta pleomorpha	2	0.01%
Acetoanaerobium sticklandii	2	0.01%
Clostridium isatidis	2	0.01%
Prevotella micans	2	0.01%
Acetoanaerobium pronyense	2	0.01%
Acetanaerobacterium elongatum	2	0.01%
Defluviitalea raffinosedens	2	0.01%
Clostridium lactatifermentans	2	0.01%
Bulleidia extructa	2	0.01%
Parasutterella excrementihominis	2	0.01%
unclassified Veillonellaceae	2	0.01%
Collinsella stercoris	2	0.01%
Bacteroides uniformis	2	0.01%
unclassified Lactobacillaceae	2	0.01%
Clostridium colicanis	2	0.01%
Prevotella amnii	2	0.01%
Pseudoflavonifractor capillosus	2	0.01%
Holdemania massiliensis	2	0.01%
Lactobacillus sakei	2	0.01%
unclassified Rikenella	2	0.01%
Bifidobacterium longum	2	0.01%
Parabacteroides goldsteinii	2	0.01%
Prevotella saccharolytica	2	0.01%
Tepidibacter mesophilus	2	0.01%
Methylocystis echinoides	2	0.01%
Desulfovibrio desulfuricans	2	0.01%
Porphyromonas pasteri	2	0.01%
unclassified Faecalibacterium	2	0.01%
Eubacterium ventriosum	2	0.01%
Eubacterium siraeum	2	0.01%
Lactobacillus antri	2	0.01%
Clostridium hungatei	2	0.01%
Papillibacter cinnamivorans	2	0.01%
Bacteroides gallinaceum	2	0.01%
Prevotella marshii	2	0.01%
Lactobacillus gasseri	2	0.01%
Romboutsia lituseburensis	2	0.01%
Eisenbergiella massiliensis	2	0.01%
Ethanoligenens harbinense	2	0.01%
Caloramator fervidus	2	0.01%
Pseudomonas fluorescens	2	0.01%
Lactobacillus hominis	2	0.01%
Oscillibacter valericigenes	2	0.01%
Bacteroides graminisolvens	2	0.01%
Lactobacillus psittaci	2	0.01%
type II	2	0.01%
Casaltella massiliensis	2	0.01%

Eubacterium infirmum	2	0.01%
Oceanirhabdus sediminicola	2	0.01%
Mogibacterium vescum	1	0%
Mucispirillum schaedleri	1	0%
Propionispira raffinosivorans	1	0%
Natronincola histidinovorans	1	0%
unclassified Oscillospira	1	0%
beta proteobacterium	1	0%
Sutterella parvirubra	1	0%
Slackia exigua	1	0%
Clostridium sufflavum	1	0%
Lactobacillus salivarius	1	0%
Blautia hydrogenotrophica	1	0%
Clostridium sulfidigenes	1	0%
Lachnoanaerobaculum saburreum	1	0%
Leptothrix mobilis	1	0%
unclassified Holophaga	1	0%
Fibrobacter intestinalis	1	0%
Lactobacillus coleohominis	1	0%
Clostridium amygdalinum	1	0%
Helicobacter trogontum	1	0%
Bacteroides caccae	1	0%
unclassified Actinobacteria	1	0%
Alistipes putredinis	1	0%
unclassified Subdoligranulum	1	0%
unclassified Dialister	1	0%
Lactobacillus mucosae	1	0%
Terrimicrobium sacchariphilum	1	0%
Lactonifactor longoviformis	1	0%
unclassified Sutterella	1	0%
Pseudobutyrivibrio ruminis	1	0%
Bittarella massiliensis	1	0%
Selenomonas artemidis	1	0%
Acidaminobacter hydrogenoformans	1	0%
unclassified Sphingobium	1	0%
unclassified Chloracidobacterium	1	0%
unclassified Proteus	1	0%
Sporosarcina soli	1	0%
Prevotella corporis	1	0%
Lactobacillus tucceti	1	0%
Bacillus gaemokensis	1	0%
unclassified Gammaproteobacteria	1	0%
Dysgonomonas termitidis	1	0%
unclassified Lachnospira	1	0%
Shuttleworthia satelles	1	0%
Prevotella oulorum	1	0%
Campylobacter coli	1	0%
unclassified Porphyromonadaceae	1	0%
alpha proteobacterium	1	0%
unclassified Porphyromonas	1	0%

Thermotalea metallivorans	1	0%
unclassified Longilinea	1	0%
Desulfotomaculum tongense	1	0%
Pedosphaera parvula	1	0%
Conexibacter arvalis	1	0%
Clostridium aminobutyricum	1	0%
Clostridium colinum	1	0%
Caloranaerobacter azorensis	1	0%
unclassified Bacillus	1	0%
unclassified Enterococcus	1	0%
Clostridium perfringens	1	0%
Clostridium bolteae	1	0%
Bacillus niacini	1	0%
Blautia hansenii	1	0%
Pediococcus ethanolidurans	1	0%
Streptococcus azizii	1	0%
Prevotella timonensis	1	0%
Clostridium putrefaciens	1	0%
unclassified Papillibacter	1	0%
Clostridium favososporum	1	0%
Aeriscardovia aeriphila	1	0%
Clostridium longisporum	1	0%
Intestinimonas gabonensis	1	0%
Clostridium scindens	1	0%
Lachnoanaerobaculum umeaense	1	0%
Anaerotruncus colihominis	1	0%
Prevotella oryzae	1	0%
Prevotella fusca	1	0%
Aminicella lysinilytica	1	0%
unclassified Lactobacillus	1	0%
Bacteroides salyersiae	1	0%
Pseudarthrobacter sulfonivorans	1	0%
unclassified Thermomonas	1	0%
Clostridium bornimense	1	0%
Clostridium carnis	1	0%
Georgfuchsia toluolica	1	0%
Prevotella jejuni	1	0%
Clostridium josui	1	0%
Clostridium neopropionicum	1	0%
unclassified Betaproteobacteria	1	0%
Methanosphaera cuniculi	1	0%
Dehalogenimonas alkenigignens	1	0%
Bacteroides neonati	1	0%
Colletotrichum dracaenophilum	1	0%
Prevotella multisaccharivorax	1	0%
unclassified Oribacterium	1	0%
Catabacter hongkongensis	1	0%
Acetatifactor muris	1	0%
Lactobacillus suebicus	1	0%
Desulfosporosinus orientis	1	0%

Bacillus nealsonii	1	0%
unclassified Erysipelotrichia	1	0%
Slackia piriformis	1	0%
Helicobacter equorum	1	0%
Devosia ginsengisoli	1	0%
Clostridium glycyrrhizinilyticum	1	0%
Lactobacillus gallinarum	1	0%
Lactobacillus intestinalis	1	0%
Bifidobacterium breve	1	0%
Dialister propionicifaciens	1	0%
Christensenella minuta	1	0%
Gottschalkia acidurici	1	0%
Solirubrobacter phytolaccae	1	0%
Clostridium methoxybenzovorans	1	0%
mixed culture	1	0%
Lewinella nigricans	1	0%
Bacteroides ovatus	1	0%
Roseburia cecicola	1	0%
Tepidimicrobium xylanilyticum	1	0%
Clostridium taeniosporum	1	0%
Sporosarcina psychrophila	1	0%
unclassified Olsenella	1	0%
Alistipes massiliensis	1	0%
unclassified Acidobacterium	1	0%
Pediococcus parvulus	1	0%
unclassified Erysipelothrix	1	0%
Clostridium uliginosum	1	0%
Prevotella disiens	1	0%
Caloramator proteoclasticus	1	0%
Bacteroides paurosaccharolyticus	1	0%
Bacillus andreraoultii	1	0%
Bacteroides oleiciplenus	1	0%
Parvibacter caecicola	1	0%
Brevibacillus fulvus	1	0%
endosymbiont of	1	0%
Salinispora pacifica	1	0%
Bacteroides vulgatus	1	0%
metal-contaminated soil	1	0%
